# Supplementary material for: Aberrations in Notch-Hedgehog signalling reveal cancer stem cells harbouring conserved oncogenic properties associated with hypoxia and immunoevasion
Source: Br J Cancer. 2019 Sep 16;121(8):666–78. doi: 10.1038/s41416-019-0572-9 (PMC6889439; doi:10.1038/s41416-019-0572-9)
Supplement: Supplementary file 1 — Supplementary figures and tables [file 41416_2019_572_MOESM1_ESM.pdf]

Supplementary figure and table legends

Figure S1. Prognosis of each of the 13 signature genes in 20 cancer types determined using Cox regression analyses. Rows (Notch-Hedgehog driver genes) and columns (cancer types) were ordered using hierarchical clustering (Euclidean distance metric). Boxes in the lightest pink colour represent non-prognostic genes. Heatmap intensities represent hazard ratios of prognostic genes that were significant (P<0.05).

Figure S2. Expression distribution of the 13 signature genes in tumour and non-tumour samples of five cancers. Nonparametric Mann-Whitney-Wilcoxon tests were employed to determine whether there were significant differences in expression distributions. Asterisks represent significant P values: * < 0.05, ** < 0.005, ***<0.0005 and ****<0.00005. ns: non-significant.

Figure S3. Differentially expressed genes between Q4 and Q1 patients as determined by their 13-gene scores. (A) Venn diagram depicts a five-way comparison of DEGs (-1.5 > $\log_2$ fold-change > 1.5, P<0.01) identified from five cancer cohorts. **(B)** Volcano plots illustrate the distribution of DEGs (in pink). Non-significant genes were represented as grey dots. Genes implicated in other stem cell-related signalling modules were annotated and colour-coded.

Figure S4. Correlations between the Notch-Hedgehog signature and other CSC markers. Scatter plots depict the associations between 13-gene scores and nine CSC marker expression profiles in five cancer types. P values were determined by Spearman's rank-order correlation analyses.

Table S1. List of 72 Notch-Hedgehog pathway genes.

Table S2. List of the number of tumour and non-tumour samples obtained from TCGA along with abbreviation decodes.

Table S3. Univariate and multivariate Cox proportional hazards regression to determine the independence of the signature with other clinicopathological risk factors. Significant P values were highlighted in bold.

Table S4. Differentially expressed genes between Q4 and Q1 patient groups as determined by the signature in five cancer types.

Figure S1

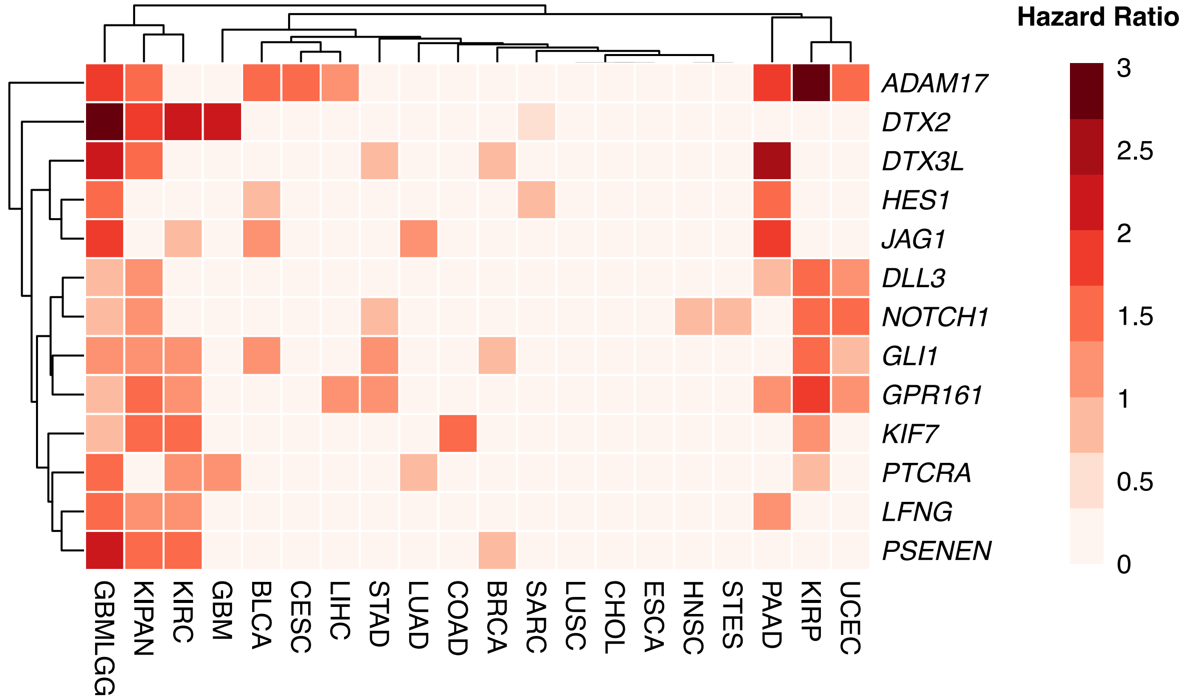

Hazard Ratio
3
2.5
2
1.5
1
0.5
0
ADAM17
DTX2
DTX3L
HES1
JAG1
DLL3
NOTCH1
GLI1
GPR161
KIF7
PTCRA
LFNG
PSENEN
GBMLGG
KIPAN
KIRC
GBM
BLCA
CESC
LIHC
STAD
LUAD
COAD
BRCA
SARC
LUSC
CHOL
ESCA
HNSC
STES
PAAD
KIRP
UCEC

Figure S2

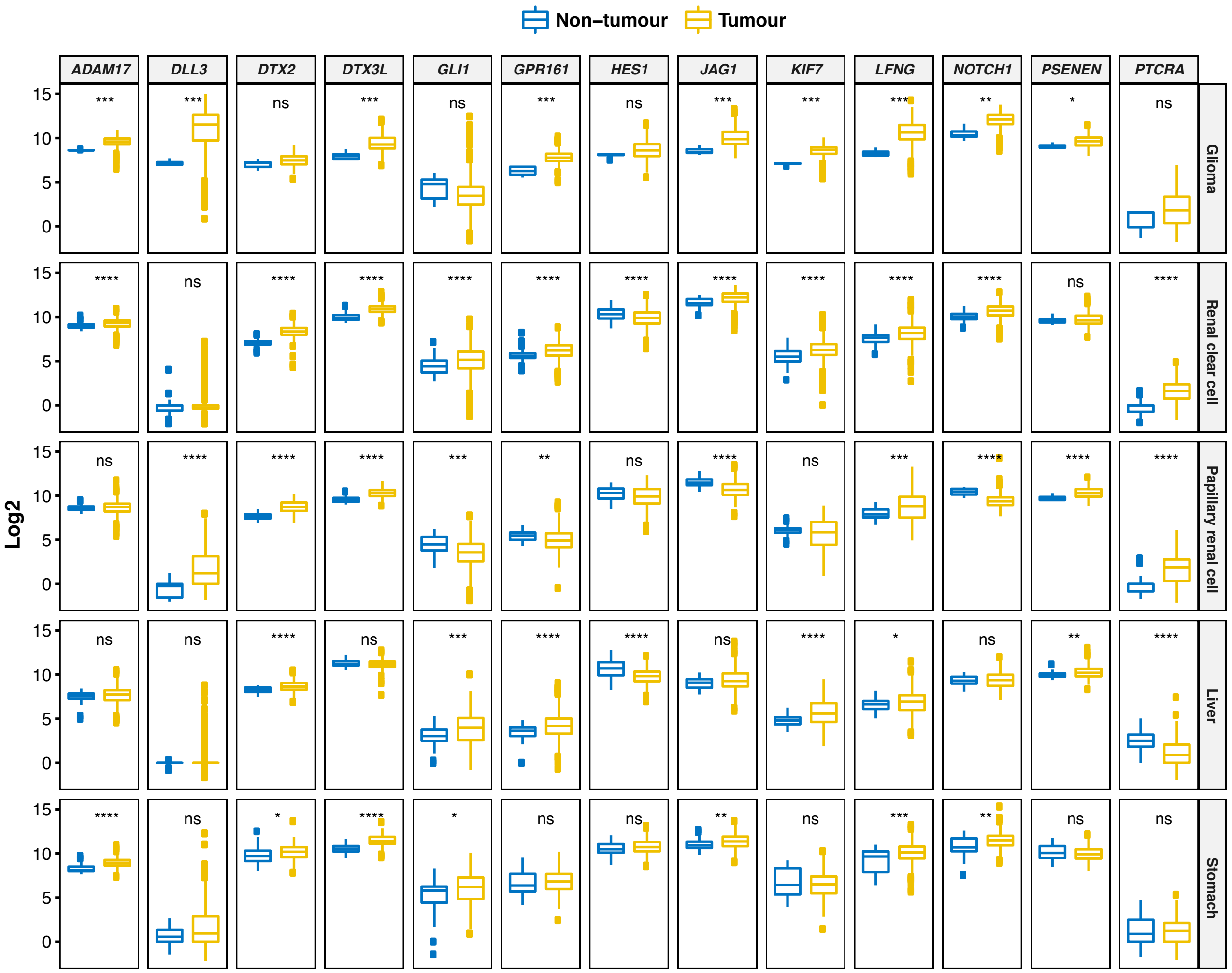
Non–tumour
Tumour
ADAM17
DLL3
DTX2
DTX3L
GLI1
GPR161
HES1
JAG1
KIF7
LFNG
NOTCH1
PSENEN
PTCRA
Glioma
Renal clear cell
Papillary renal cell
Liver
Stomach
Log2
0
5
10
15
*** *** ns *** ns *** ns *** *** *** ** * ns
**** ns **** **** **** **** **** **** **** **** **** ns ****
ns **** **** **** *** ** ns **** ns *** **** **** ****
ns ns **** ns *** **** **** ns **** * ns ** ****
**** ns * **** * ns ns ** ns *** ** ns ns

Figure S3

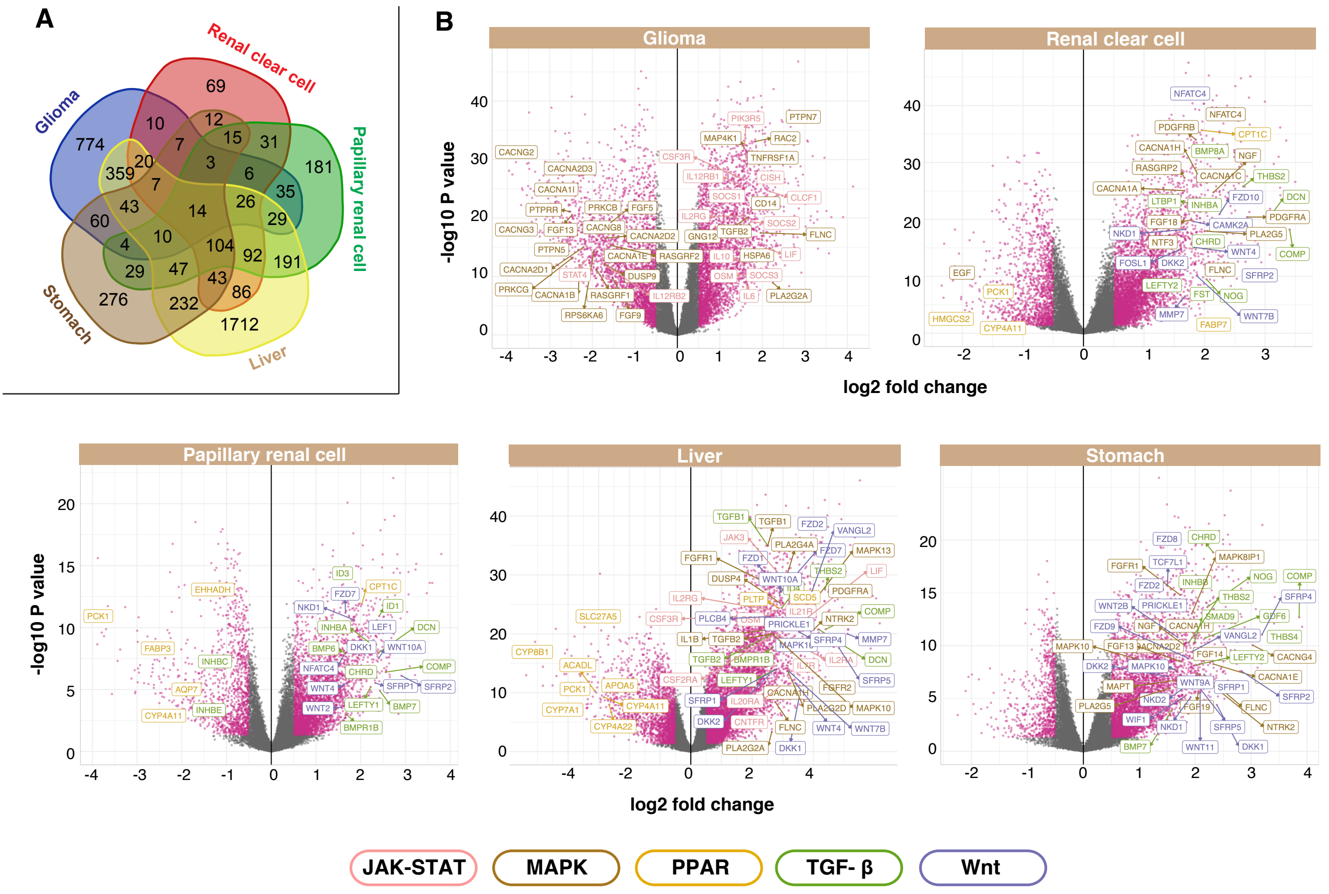

A
Glioma
Renal clear cell
Papillary renal cell
Stomach
Liver
774
69
181
276
1712
10
12
7
15
31
20
359
3
6
35
7
43
14
26
29
60
4
10
104
92
191
29
47
43
86
232
B
Glioma
Renal clear cell
Papillary renal cell
Liver
Stomach
-log10 P value
log2 fold change
JAK-STAT
MAPK
PPAR
TGF- β
Wnt

Figure S4

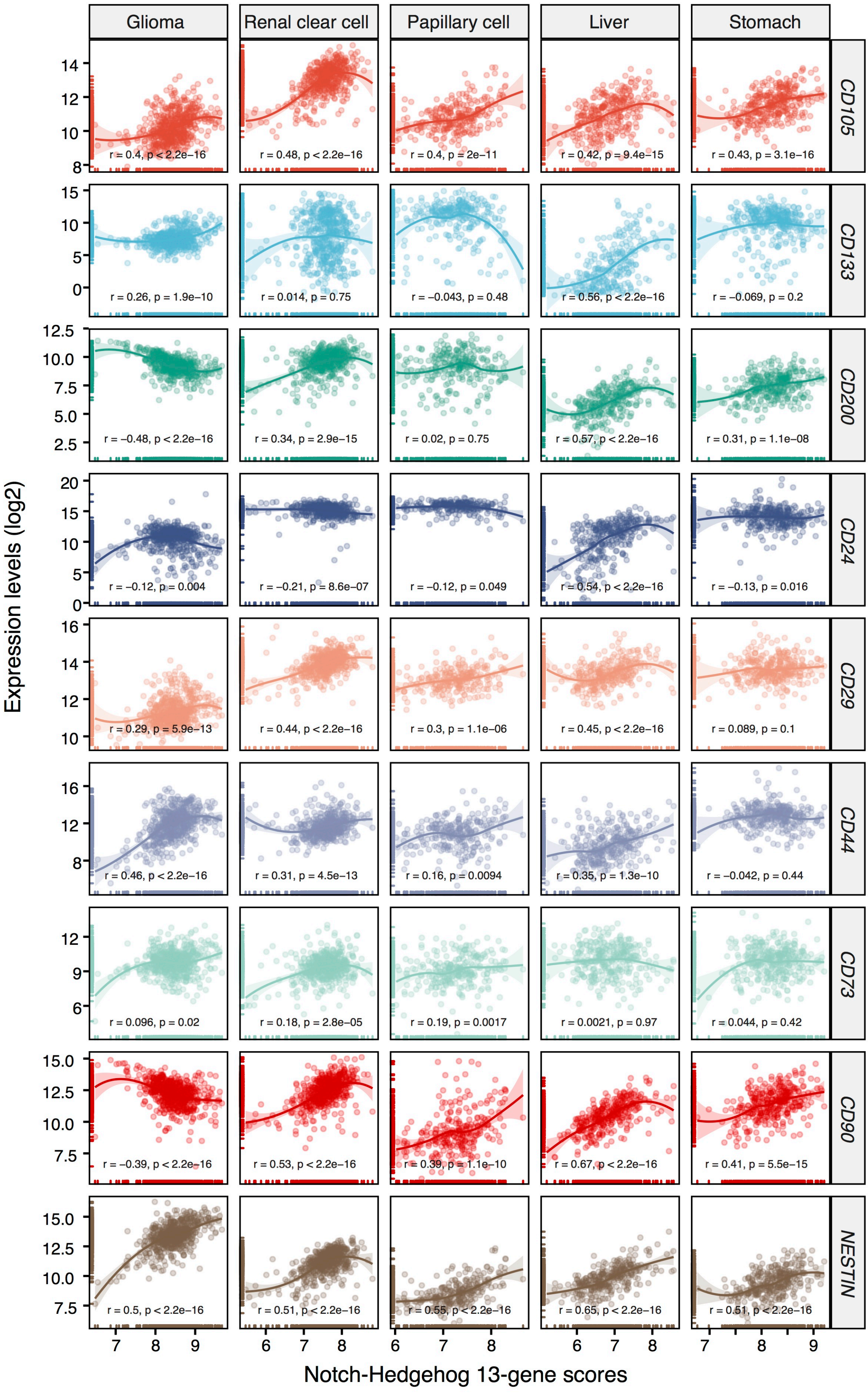

Glioma
Renal clear cell
Papillary cell
Liver
Stomach
CD105
CD133
CD200
CD24
CD29
CD44
CD73
CD90
NESTIN
Expression levels (log2)
Notch-Hedgehog 13-gene scores
r = 0.4, p < 2.2e−16
r = 0.48, p < 2.2e−16
r = 0.4, p = 2e−11
r = 0.42, p = 9.4e−15
r = 0.43, p = 3.1e−16
r = 0.26, p = 1.9e−10
r = 0.014, p = 0.75
r = −0.043, p = 0.48
r = 0.56, p < 2.2e−16
r = −0.069, p = 0.2
r = −0.48, p < 2.2e−16
r = 0.34, p = 2.9e−15
r = 0.02, p = 0.75
r = 0.57, p < 2.2e−16
r = 0.31, p = 1.1e−08
r = −0.12, p = 0.004
r = −0.21, p = 8.6e−07
r = −0.12, p = 0.049
r = 0.54, p < 2.2e−16
r = −0.13, p = 0.016
r = 0.29, p = 5.9e−13
r = 0.44, p < 2.2e−16
r = 0.3, p = 1.1e−06
r = 0.45, p < 2.2e−16
r = 0.089, p = 0.1
r = 0.46, p < 2.2e−16
r = 0.31, p = 4.5e−13
r = 0.16, p = 0.0094
r = 0.35, p = 1.3e−10
r = −0.042, p = 0.44
r = 0.096, p = 0.02
r = 0.18, p = 2.8e−05
r = 0.19, p = 0.0017
r = 0.0021, p = 0.97
r = 0.044, p = 0.42
r = −0.39, p < 2.2e−16
r = 0.53, p < 2.2e−16
r = 0.39, p = 1.1e−10
r = 0.67, p < 2.2e−16
r = 0.41, p = 5.5e−15
r = 0.5, p < 2.2e−16
r = 0.51, p < 2.2e−16
r = 0.55, p < 2.2e−16
r = 0.65, p < 2.2e−16
r = 0.51, p < 2.2e−16

Table S1. List of 72 Notch-Hedgehog pathway genes.

Entrez	Gene Symbol	Pathway	Description
408	ARRB1	Hedgehog	arrestin beta 1
409	ARRB2	Hedgehog	arrestin beta 2
596	BCL2	Hedgehog	BCL2, apoptosis regulator
91653	BOC	Hedgehog	BOC cell adhesion associated, oncogene regulated
50937	CDON	Hedgehog	cell adhesion associated, oncogene regulated
53944	CSNK1G1	Hedgehog	casein kinase 1 gamma 1
1455	CSNK1G2	Hedgehog	casein kinase 1 gamma 2
1456	CSNK1G3	Hedgehog	casein kinase 1 gamma 3
8454	CUL1	Hedgehog	cullin 1
8452	CUL3	Hedgehog	cullin 3
50846	DHH	Hedgehog	desert hedgehog
2121	EVC	Hedgehog	EvC ciliary complex subunit 1
132884	EVC2	Hedgehog	EvC ciliary complex subunit 2
2619	GAS1	Hedgehog	growth arrest specific 1
2735	GLI1	Hedgehog	GLI family zinc finger 1
2736	GLI2	Hedgehog	GLI family zinc finger 2
2737	GLI3	Hedgehog	GLI family zinc finger 3
23432	GPR161	Hedgehog	G protein-coupled receptor 161
156	GRK2	Hedgehog	G protein-coupled receptor kinase 2
157	GRK3	Hedgehog	G protein-coupled receptor kinase 3
64399	HHIP	Hedgehog	hedgehog interacting protein
3549	IHH	Hedgehog	indian hedgehog
11127	KIF3A	Hedgehog	kinesin family member 3A
374654	KIF7	Hedgehog	kinesin family member 7
4036	LRP2	Hedgehog	LDL receptor related protein 2
5727	PTCH1	Hedgehog	patched 1
8643	PTCH2	Hedgehog	patched 2
6469	SHH	Hedgehog	sonic hedgehog
6608	SMO	Hedgehog	smoothened, frizzled class receptor
57154	SMURF1	Hedgehog	SMAD specific E3 ubiquitin protein ligase 1
64750	SMURF2	Hedgehog	SMAD specific E3 ubiquitin protein ligase 2
8405	SPOP	Hedgehog	speckle type BTB/POZ protein
339745	SPOPL	Hedgehog	speckle type BTB/POZ protein like
51684	SUFU	Hedgehog	SUFU negative regulator of hedgehog signaling
6868	ADAM17	Notch	ADAM metallopeptidase domain 17
51107	APH1A	Notch	aph-1 homolog A, gamma-secretase subunit
83464	APH1B	Notch	aph-1 homolog B, gamma-secretase subunit
9541	CIR1	Notch	corepressor interacting with RBPJ, 1
28514	DLL1	Notch	delta like canonical Notch ligand 1
10683	DLL3	Notch	delta like canonical Notch ligand 3
54567	DLL4	Notch	delta like canonical Notch ligand 4
1840	DTX1	Notch	deltex E3 ubiquitin ligase 1
113878	DTX2	Notch	deltex E3 ubiquitin ligase 2
196403	DTX3	Notch	deltex E3 ubiquitin ligase 3
151636	DTX3L	Notch	deltex E3 ubiquitin ligase 3L
23220	DTX4	Notch	deltex E3 ubiquitin ligase 4
3065	HDAC1	Notch	histone deacetylase 1
3066	HDAC2	Notch	histone deacetylase 2
3280	HES1	Notch	hes family bHLH transcription factor 1
388585	HES5	Notch	hes family bHLH transcription factor 5
182	JAG1	Notch	jagged 1
3714	JAG2	Notch	jagged 2
3955	LFNG	Notch	LFNG O-fucosylpeptide 3-beta-N-acetylglucosaminyltransferase

9794	MAML1	Notch	mastermind like transcriptional coactivator 1
84441	MAML2	Notch	mastermind like transcriptional coactivator 2
55534	MAML3	Notch	mastermind like transcriptional coactivator 3
4242	MFNG	Notch	MFNG O-fucosylpeptide 3-beta-N-acetylglucosaminyltransferase
9612	NCOR2	Notch	nuclear receptor corepressor 2
23385	NCSTN	Notch	nicastrin
4851	NOTCH1	Notch	notch 1
4853	NOTCH2	Notch	notch 2
4854	NOTCH3	Notch	notch 3
4855	NOTCH4	Notch	notch 4
8650	NUMB	Notch	NUMB, endocytic adaptor protein
9253	NUMBL	Notch	NUMB like, endocytic adaptor protein
5664	PSEN2	Notch	presenilin 2
55851	PSENEN	Notch	presenilin enhancer, gamma-secretase subunit
171558	PTCRA	Notch	pre T cell antigen receptor alpha
3516	RBPJ	Notch	recombination signal binding protein for immunoglobulin kappa J region
11317	RBPJL	Notch	recombination signal binding protein for immunoglobulin kappa J region like
5986	RFNG	Notch	RFNG O-fucosylpeptide 3-beta-N-acetylglucosaminyltransferase
22938	SNW1	Notch	SNW domain containing 1

Table S2. Abbreviations and number of tumour and non-tumour samples in TCGA cancers.

Cohort (TCGA abbreviations)	Non-tumour #	Tumour #	Description
BLCA	19	408	Bladder Urothelial Carcinoma
BRCA	112	10939	Breast invasive carcinoma
CESC	3	304	Cervical squamous cell carcinoma and endocervical adenocarcinoma
CHOL	9	36	Cholangiocarcinoma
COAD	41	285	Colon adenocarcinoma
ESCA	11	184	Oesophageal carcinoma
GBM	5	153	Glioblastoma multiforme
GBMLGG	5	669	Glioma
HNSC	44	520	Head and Neck squamous cell carcinoma
KICH	25	66	Kidney Chromophobe
KIPAN	129	889	Pan-kidney cohort
KIRC	72	533	Kidney renal clear cell carcinoma
KIRP	32	290	Kidney renal papillary cell carcinoma
LIHC	50	371	Liver hepatocellular carcinoma
LUAD	59	515	Lung adenocarcinoma
LUSC	51	501	Lung squamous cell carcinoma
PAAD	4	178	Pancreatic adenocarcinoma
SARC	2	259	Sarcoma
STAD	35	415	Stomach adenocarcinoma
STES	46	599	Stomach and Oesophageal carcinoma
UCEC	11	370	Uterine Corpus Endometrial Carcinoma

Adapted from previous publications employing TCGA datasets (2, 22, 51).

Table S3. Univariate and multivariate Cox proportional hazards regression to determine the independence of the signature with other clinicopathological risk factors.

	Hazard Ratio (95% CI)	*P*-value
**All gliomas**	**Univariate**	
Signature (Q2 vs. Q1)	1.026 (0.630 - 1.672)	0.92
Signature (Q3 vs. Q1)	1.754 (1.106 - 2.783)	**0.017**
Signature (Q4 vs. Q1)	3.386 (2.209 - 5.188)	**2.16E-08**
**Astrocytoma**	**Univariate**	
Signature (Q2 vs. Q1)	1.068 (0.448 - 2.548)	0.88
Signature (Q3 vs. Q1)	1.487 (0.660 - 3.351)	0.34
Signature (Q4 vs. Q1)	2.535 (1.148 - 5.597)	**0.021**
**Oligoastrocytoma**	**Univariate**	
Signature (Q2 vs. Q1)	0.516 (0.110 - 2.414)	0.4
Signature (Q3 vs. Q1)	1.649 (0.429 - 6.339)	0.47
Signature (Q4 vs. Q1)	4.169 (1.329 - 13.076)	**0.014**
**Glioblastoma multiforme**	**Univariate**	
Signature (Q2 vs. Q1)	1.065 (0.477 - 2.381)	0.88
Signature (Q3 vs. Q1)	2.322 (1.043 - 5.167)	**0.039**
Signature (Q4 vs. Q1)	2.163 (1.092 - 4.713)	**0.042**

**Clear cell renal cell**	**Univariate**	
Signature (Q2 vs. Q1)	1.247 (0.787 - 1.975)	0.34
Signature (Q3 vs. Q1)	1.438 (0.896 - 2.308)	0.13
Signature (Q4 vs. Q1)	2.177 (1.406 - 3.371)	**0.00048**
TNM staging	1.87 (1.641 - 2.132)	**2.00E-16**
	**Multivariate**	
Signature (Q2 vs. Q1)	1.123 (0.708 - 1.782)	0.62
Signature (Q3 vs. Q1)	1.226 (0.763 - 1.970)	0.39
Signature (Q4 vs. Q1)	1.731 (1.114 - 2.690)	**0.014**
TNM staging	1.834 (1.607 - 2.093)	**2.00E-16**

**Papillary renal cell**	**Univariate**	
Signature (Q2 vs. Q1)	1.514 (0.427 - 5.368)	0.52
Signature (Q3 vs. Q1)	1.075 (0.269 - 4.298)	0.91
Signature (Q4 vs. Q1)	4.881 (1.600 - 14.886)	**0.0053**
TNM staging	2.710 (1.893 - 3.878)	**5.03E-08**
	**Multivariate**	
Signature (Q2 vs. Q1)	1.223 (0.343 - 4.366)	0.75
Signature (Q3 vs. Q1)	0.650 (0.154 - 2.739)	0.56
Signature (Q4 vs. Q1)	2.297 (1.072 - 7.280)	**0.042**
TNM staging	2.677 (1.765 - 4.060)	**3.60E-06**

**Liver**	**Univariate**	
Signature (Q2 vs. Q1)	1.994 (1.014 - 3.922)	**0.045**
Signature (Q3 vs. Q1)	1.414 (0.691 - 2.891)	0.34

Signature (Q4 vs. Q1)	2.627 (1.363 - 5.062)	**0.0039**
TNM staging	2.033 (1.595 - 2.592)	**9.96E-09**
	**Multivariate**	
Signature (Q2 vs. Q1)	1.659 (0.840 - 3.275)	0.14
Signature (Q3 vs. Q1)	1.048 (0.506 - 2.168)	0.9
Signature (Q4 vs. Q1)	2.146 (1.109 - 4.153)	**0.024**
TNM staging	2.012 (1.572 - 2.575)	**2.80E-08**

**Stomach**	**Univariate**	
Signature (Q2 vs. Q1)	0.906 (0.442 - 1.857)	0.79
Signature (Q3 vs. Q1)	2.118 (1.129 - 3.972)	**0.019**
Signature (Q4 vs. Q1)	2.217 (1.175 - 4.163)	**0.014**
TNM staging	1.372 (1.067 - 1.765)	**0.038**
	**Multivariate**	
Signature (Q2 vs. Q1)	0.929. (0.453 - 1.905)	0.84
Signature (Q3 vs. Q1)	2.012 (1.071 - 3.779)	**0.029**
Signature (Q4 vs. Q1)	2.161 (1.148 - 4.068)	**0.017**
TNM staging	1.309 (1.014 - 1.691)	**0.039**

Univariate values for TNM staging were in accordance with our previous reports utilizing TCGA datasets (2, 22, 51)

Table S4. Differentially expressed genes between Q4 and Q1 patient groups as determined by the signature in five cancer types.

Entrez_id	Log2 Fold change	P value	Cancer type
55530	-3.958574599	8.04E-29	Glioma
2566	-3.881000347	3.55E-28	Glioma
10369	-3.797579137	2.77E-30	Glioma
2902	-3.674479943	6.20E-23	Glioma
23040	-3.653499731	7.93E-26	Glioma
93426	-3.641664834	1.59E-25	Glioma
492	-3.633939479	1.73E-31	Glioma
3747	-3.539800947	1.25E-24	Glioma
9671	-3.535759223	5.04E-30	Glioma
23316	-3.51141395	3.43E-26	Glioma
51761	-3.49581953	3.82E-30	Glioma
3350	-3.488842624	8.58E-39	Glioma
9699	-3.483240773	3.92E-29	Glioma
3897	-3.454753831	1.00E-25	Glioma
2554	-3.423884998	4.35E-20	Glioma
121256	-3.418075299	3.63E-24	Glioma
2849	-3.391877022	9.84E-22	Glioma
205147	-3.379222424	2.57E-25	Glioma
11255	-3.3471798	1.21E-28	Glioma
10368	-3.321108255	1.79E-20	Glioma
10814	-3.319306981	2.73E-23	Glioma
84530	-3.318310329	1.42E-32	Glioma
9118	-3.307808134	1.30E-24	Glioma
6860	-3.30497325	2.44E-25	Glioma
55359	-3.269098304	2.33E-26	Glioma
5816	-3.249161004	1.24E-24	Glioma
1113	-3.247732847	7.25E-22	Glioma
4741	-3.244810562	2.88E-18	Glioma
3361	-3.243797252	3.43E-27	Glioma
6534	-3.236524021	1.49E-17	Glioma
440730	-3.221129479	1.36E-18	Glioma
57586	-3.183691084	8.26E-25	Glioma
7447	-3.173754025	3.11E-18	Glioma
343702	-3.147894749	5.29E-28	Glioma
29953	-3.141740568	1.37E-22	Glioma
57582	-3.138784539	3.52E-22	Glioma
7138	-3.111162371	2.29E-30	Glioma
2561	-3.109945912	1.64E-20	Glioma
389941	-3.106864402	1.60E-19	Glioma
4747	-3.07889045	3.79E-15	Glioma
6616	-3.073607923	1.34E-28	Glioma
2557	-3.066350575	5.71E-21	Glioma
440073	-3.05950583	6.77E-27	Glioma
63974	-3.051335942	2.21E-19	Glioma
27012	-3.049141187	1.65E-18	Glioma
1007	-3.048697877	2.82E-26	Glioma
23109	-3.038861357	4.66E-24	Glioma
6620	-3.021996986	3.60E-27	Glioma
26507	-3.019654163	1.12E-26	Glioma
9312	-3.006745059	3.90E-30	Glioma
57574	-3.000595434	2.94E-26	Glioma

6854	-2.999898549	1.94E-22	Glioma
6511	-2.996966925	2.30E-20	Glioma
9840	-2.991567568	7.46E-16	Glioma
50632	-2.991535668	5.59E-17	Glioma
114569	-2.99147504	1.69E-19	Glioma
25830	-2.986089636	2.32E-18	Glioma
57468	-2.973676295	8.95E-22	Glioma
1016	-2.962826903	3.77E-23	Glioma
338811	-2.961822508	1.34E-39	Glioma
348980	-2.953706	1.37E-18	Glioma
5650	-2.950891999	4.11E-23	Glioma
200407	-2.934150671	4.12E-18	Glioma
146713	-2.927227632	4.69E-20	Glioma
83482	-2.922520937	8.66E-21	Glioma
29993	-2.918623839	3.60E-17	Glioma
222008	-2.913155146	7.42E-19	Glioma
57495	-2.90455031	4.99E-20	Glioma
2558	-2.902579088	2.09E-16	Glioma
283455	-2.896435788	9.69E-23	Glioma
869	-2.887625739	4.42E-23	Glioma
56853	-2.880761507	4.65E-25	Glioma
59350	-2.876249677	1.81E-23	Glioma
246213	-2.87587491	4.61E-18	Glioma
441061	-2.869801816	1.37E-35	Glioma
9899	-2.866600971	4.09E-16	Glioma
40	-2.863202114	2.41E-24	Glioma
57624	-2.861083196	1.80E-29	Glioma
147381	-2.858909315	2.00E-23	Glioma
768096	-2.853751021	5.92E-30	Glioma
285780	-2.851035289	1.30E-21	Glioma
6262	-2.846823469	3.77E-16	Glioma
548596	-2.842084019	3.82E-25	Glioma
57030	-2.838682092	8.78E-13	Glioma
2562	-2.833968787	1.37E-23	Glioma
9758	-2.829039127	3.46E-21	Glioma
1128	-2.82189178	8.05E-23	Glioma
114788	-2.817458958	5.89E-16	Glioma
9892	-2.815804538	4.75E-22	Glioma
284424	-2.813070812	3.14E-25	Glioma
4168	-2.807437032	3.24E-26	Glioma
11197	-2.806684595	1.21E-12	Glioma
51046	-2.806609562	1.39E-19	Glioma
815	-2.804378429	2.46E-17	Glioma
55799	-2.803151294	1.62E-27	Glioma
114824	-2.800944985	8.13E-25	Glioma
158696	-2.799611841	9.81E-32	Glioma
5798	-2.791228426	1.92E-23	Glioma
613212	-2.788264407	1.75E-19	Glioma
440279	-2.784880204	8.36E-17	Glioma
8001	-2.780417243	3.87E-21	Glioma
26032	-2.77735275	2.69E-25	Glioma
253314	-2.774880952	4.17E-28	Glioma
84684	-2.758747932	2.57E-25	Glioma
5126	-2.75575774	4.51E-18	Glioma
5999	-2.754820852	8.51E-21	Glioma
887	-2.750153351	6.69E-19	Glioma
1159	-2.748958017	2.10E-26	Glioma
56341	-2.738347832	7.60E-22	Glioma

23217	-2.721649411	3.74E-21	Glioma
2572	-2.716153576	1.28E-16	Glioma
51617	-2.711386478	5.25E-22	Glioma
885	-2.711269472	1.63E-15	Glioma
256714	-2.704134952	8.74E-17	Glioma
388662	-2.700808269	5.65E-20	Glioma
57369	-2.700588572	6.25E-28	Glioma
154790	-2.699466735	4.24E-18	Glioma
115827	-2.696470204	7.24E-27	Glioma
58157	-2.694133102	4.76E-17	Glioma
2556	-2.693254027	9.35E-21	Glioma
3788	-2.690382314	3.06E-19	Glioma
4745	-2.687219567	2.15E-18	Glioma
758	-2.686960723	3.22E-16	Glioma
146206	-2.680211345	1.07E-30	Glioma
9615	-2.674905195	4.28E-15	Glioma
10740	-2.67134061	9.41E-29	Glioma
7781	-2.66841066	1.08E-13	Glioma
1114	-2.663834237	4.09E-28	Glioma
222865	-2.652628434	6.01E-19	Glioma
85352	-2.651478282	3.47E-19	Glioma
22999	-2.647287184	1.71E-25	Glioma
84539	-2.647014412	1.77E-19	Glioma
6857	-2.646914297	8.01E-19	Glioma
402381	-2.646010608	8.75E-19	Glioma
57084	-2.64072509	4.98E-17	Glioma
8911	-2.640466909	9.04E-24	Glioma
401145	-2.639418959	3.51E-35	Glioma
1258	-2.637450252	2.52E-19	Glioma
23426	-2.629451078	3.15E-25	Glioma
2914	-2.628643582	9.77E-24	Glioma
57709	-2.62779971	4.40E-26	Glioma
642968	-2.618227232	9.22E-19	Glioma
2834	-2.612547513	2.00E-12	Glioma
285696	-2.606848043	3.07E-32	Glioma
26038	-2.604500189	1.01E-16	Glioma
57172	-2.603893858	2.56E-14	Glioma
195814	-2.600973293	8.41E-21	Glioma
117154	-2.597346024	5.77E-17	Glioma
140679	-2.596651751	4.92E-15	Glioma
8437	-2.594784802	1.82E-14	Glioma
6751	-2.591525883	1.52E-15	Glioma
51412	-2.590609036	1.47E-17	Glioma
400120	-2.581080026	1.29E-16	Glioma
11075	-2.574244508	1.90E-14	Glioma
7139	-2.570693835	4.28E-17	Glioma
6853	-2.568695538	2.85E-31	Glioma
10888	-2.56768719	1.47E-20	Glioma
6545	-2.561175189	1.07E-25	Glioma
6753	-2.556796274	7.24E-23	Glioma
116443	-2.551065331	4.65E-23	Glioma
2845	-2.550862929	8.45E-17	Glioma
51764	-2.548384152	7.81E-22	Glioma
10716	-2.547006821	4.13E-15	Glioma
400591	-2.545280093	2.69E-35	Glioma
56934	-2.542387888	3.14E-16	Glioma
283985	-2.54221175	4.92E-20	Glioma
3777	-2.537822401	1.63E-26	Glioma

440508	-2.536600515	1.36E-25	Glioma
100189589	-2.535702868	4.13E-25	Glioma
7480	-2.529903597	5.66E-20	Glioma
130399	-2.52972373	1.19E-29	Glioma
57338	-2.529693852	2.31E-17	Glioma
54715	-2.52837838	2.16E-16	Glioma
23072	-2.526421111	1.30E-19	Glioma
132332	-2.524694707	2.11E-17	Glioma
389206	-2.522005214	5.97E-23	Glioma
151009	-2.519162254	6.33E-17	Glioma
5801	-2.5186245	8.10E-22	Glioma
242	-2.518384398	7.27E-34	Glioma
256536	-2.517966202	1.86E-19	Glioma
118427	-2.510918698	3.38E-16	Glioma
151835	-2.499059881	7.39E-20	Glioma
148641	-2.4983751	1.71E-25	Glioma
55800	-2.496750587	1.86E-26	Glioma
132204	-2.496358436	5.65E-14	Glioma
10804	-2.491850433	2.51E-10	Glioma
23732	-2.483706608	7.80E-24	Glioma
6770	-2.482962928	1.72E-41	Glioma
8941	-2.480919254	1.91E-26	Glioma
6543	-2.476834978	5.68E-17	Glioma
2258	-2.475752824	3.01E-21	Glioma
2915	-2.474801477	6.89E-21	Glioma
6863	-2.47444976	9.02E-14	Glioma
7432	-2.471355115	1.50E-17	Glioma
27165	-2.471217012	2.71E-26	Glioma
84867	-2.466018975	1.13E-15	Glioma
390616	-2.465454932	6.54E-21	Glioma
5582	-2.459616265	5.90E-14	Glioma
9796	-2.457407748	2.37E-23	Glioma
1812	-2.452169905	1.44E-20	Glioma
4884	-2.451398001	5.44E-18	Glioma
55040	-2.44862636	1.25E-20	Glioma
407738	-2.448579327	2.19E-17	Glioma
220164	-2.446000066	6.01E-27	Glioma
3739	-2.439731606	7.18E-24	Glioma
3760	-2.435736184	2.25E-19	Glioma
157627	-2.430157634	3.11E-19	Glioma
5063	-2.424525231	4.07E-21	Glioma
100033802	-2.417254694	2.93E-29	Glioma
4986	-2.417059662	7.44E-17	Glioma
9362	-2.415046919	3.20E-13	Glioma
2016	-2.412304198	6.36E-15	Glioma
9143	-2.411509159	1.88E-23	Glioma
57144	-2.409398271	2.14E-18	Glioma
9148	-2.404587241	1.12E-24	Glioma
3756	-2.404466033	1.75E-24	Glioma
6549	-2.40263738	1.57E-25	Glioma
144423	-2.401094493	3.58E-21	Glioma
64409	-2.398910132	8.78E-16	Glioma
594855	-2.395796338	9.74E-18	Glioma
1993	-2.395382028	1.33E-23	Glioma
2565	-2.394760934	8.96E-14	Glioma
285954	-2.394081169	8.79E-23	Glioma
3358	-2.390371595	5.50E-19	Glioma
80309	-2.388917169	3.64E-12	Glioma

2903	-2.386413356	8.43E-17	Glioma
2830	-2.378424522	3.48E-23	Glioma
55118	-2.357383799	8.85E-18	Glioma
398	-2.357188072	1.29E-20	Glioma
645974	-2.356336087	6.21E-18	Glioma
2153	-2.355312628	6.07E-12	Glioma
283392	-2.351275366	1.40E-26	Glioma
5662	-2.350255844	1.18E-26	Glioma
1428	-2.348529059	1.17E-11	Glioma
642597	-2.342789645	3.56E-30	Glioma
345079	-2.342710159	2.50E-16	Glioma
9478	-2.342591274	1.24E-18	Glioma
255022	-2.334448301	7.21E-20	Glioma
1135	-2.333287843	2.72E-15	Glioma
79722	-2.331592808	6.27E-26	Glioma
139189	-2.324911206	2.67E-13	Glioma
2334	-2.323069831	2.90E-15	Glioma
4900	-2.321191716	1.71E-14	Glioma
26047	-2.320129315	5.16E-21	Glioma
2774	-2.317136042	1.80E-21	Glioma
2904	-2.316133597	1.47E-18	Glioma
286002	-2.315771837	3.46E-12	Glioma
646405	-2.313254303	6.25E-28	Glioma
7092	-2.313128846	4.59E-19	Glioma
11001	-2.311572696	5.76E-22	Glioma
55753	-2.311555507	2.23E-21	Glioma
284656	-2.310916592	4.90E-22	Glioma
2674	-2.308191899	1.74E-13	Glioma
774	-2.307129028	1.14E-12	Glioma
57282	-2.306697111	2.70E-22	Glioma
442117	-2.306489398	1.61E-23	Glioma
814	-2.297493293	3.35E-20	Glioma
441151	-2.296440761	2.20E-25	Glioma
27132	-2.290302567	9.86E-17	Glioma
158038	-2.283991684	3.40E-17	Glioma
4761	-2.282335707	2.86E-12	Glioma
8499	-2.282154844	1.42E-22	Glioma
92558	-2.279615664	4.92E-28	Glioma
9607	-2.276295109	1.20E-12	Glioma
5950	-2.274582646	7.08E-17	Glioma
129049	-2.273794095	3.19E-25	Glioma
9912	-2.272267766	4.16E-27	Glioma
7087	-2.27185444	5.04E-15	Glioma
23057	-2.268556449	5.73E-26	Glioma
148753	-2.267471931	1.87E-19	Glioma
400745	-2.265167427	3.11E-18	Glioma
57718	-2.264916715	7.27E-15	Glioma
3208	-2.262246888	2.18E-14	Glioma
654429	-2.260116347	2.19E-11	Glioma
728215	-2.258697291	4.27E-24	Glioma
3354	-2.257455702	4.48E-20	Glioma
79012	-2.25619858	2.71E-18	Glioma
27445	-2.252745177	1.36E-19	Glioma
30819	-2.251378721	1.85E-23	Glioma
147746	-2.250959501	2.98E-16	Glioma
50944	-2.249256481	1.26E-19	Glioma
766	-2.243700104	1.89E-15	Glioma
3362	-2.24225941	4.93E-28	Glioma

399947	-2.240621401	2.72E-12	Glioma
654790	-2.240610721	4.12E-17	Glioma
3787	-2.237269895	6.61E-10	Glioma
3356	-2.233817464	9.03E-19	Glioma
22941	-2.233649398	2.70E-18	Glioma
6623	-2.229258134	7.76E-21	Glioma
7477	-2.228027245	1.51E-14	Glioma
56955	-2.227867496	4.98E-14	Glioma
2912	-2.224953723	1.50E-21	Glioma
64211	-2.220320552	3.70E-13	Glioma
55117	-2.215385114	5.48E-13	Glioma
5138	-2.214629455	4.90E-28	Glioma
440040	-2.213790362	7.47E-19	Glioma
768097	-2.211200287	1.53E-27	Glioma
5864	-2.208380439	6.36E-31	Glioma
781	-2.207876837	9.58E-16	Glioma
3195	-2.20687427	1.34E-09	Glioma
284521	-2.195231021	5.18E-17	Glioma
23281	-2.193600831	3.27E-19	Glioma
8448	-2.193179936	3.01E-18	Glioma
23349	-2.191773189	3.13E-16	Glioma
129684	-2.190442452	2.55E-15	Glioma
402117	-2.189932503	2.44E-18	Glioma
285093	-2.187022361	2.25E-13	Glioma
9515	-2.186511382	3.83E-20	Glioma
3765	-2.184352515	3.41E-24	Glioma
6457	-2.18418871	4.18E-17	Glioma
2563	-2.177463883	1.88E-16	Glioma
6833	-2.174791212	2.07E-15	Glioma
7881	-2.173864612	1.84E-38	Glioma
284578	-2.173306499	6.54E-19	Glioma
23504	-2.168409615	1.40E-16	Glioma
152330	-2.16719958	2.93E-23	Glioma
57818	-2.16641805	8.52E-17	Glioma
143098	-2.165839662	3.19E-24	Glioma
146760	-2.164436323	4.43E-18	Glioma
286133	-2.162170066	2.76E-16	Glioma
9228	-2.158371263	1.06E-12	Glioma
5522	-2.150101212	9.12E-17	Glioma
5081	-2.141737784	5.96E-16	Glioma
51440	-2.140434787	2.82E-18	Glioma
340529	-2.140405235	1.17E-19	Glioma
51361	-2.13670482	2.23E-15	Glioma
5173	-2.13518538	4.80E-09	Glioma
1308	-2.131058517	3.48E-24	Glioma
4842	-2.128951281	2.01E-15	Glioma
6326	-2.11536845	1.27E-33	Glioma
54511	-2.114684268	1.11E-15	Glioma
138046	-2.111854893	3.65E-19	Glioma
27255	-2.105736826	1.80E-15	Glioma
26033	-2.104669747	3.96E-25	Glioma
2785	-2.10188131	4.80E-14	Glioma
260293	-2.093641466	6.53E-22	Glioma
7224	-2.090336737	3.81E-18	Glioma
150538	-2.085105761	1.95E-21	Glioma
1816	-2.084707209	4.28E-18	Glioma
4744	-2.081577993	5.28E-17	Glioma
554235	-2.079428751	6.17E-27	Glioma

11189	-2.07937267	3.31E-19	Glioma
266722	-2.076437881	1.88E-13	Glioma
1620	-2.076061743	1.25E-17	Glioma
6775	-2.075620724	4.74E-14	Glioma
3822	-2.074245666	6.58E-08	Glioma
127833	-2.07415674	1.66E-13	Glioma
2917	-2.071746477	4.46E-13	Glioma
2555	-2.070816295	1.87E-11	Glioma
6456	-2.069363668	1.15E-16	Glioma
202559	-2.066782345	3.15E-14	Glioma
6327	-2.06628138	5.86E-24	Glioma
10361	-2.066093987	4.25E-14	Glioma
2742	-2.065730506	5.14E-13	Glioma
5746	-2.065526509	1.83E-17	Glioma
63982	-2.062133703	1.43E-15	Glioma
4674	-2.059591457	6.00E-31	Glioma
1780	-2.057853005	2.34E-22	Glioma
23105	-2.056708082	5.96E-13	Glioma
286	-2.056706008	3.06E-23	Glioma
157807	-2.056333607	9.03E-18	Glioma
1394	-2.054175312	2.81E-21	Glioma
53822	-2.053998152	4.90E-14	Glioma
9185	-2.047479097	1.12E-22	Glioma
163223	-2.045507478	1.48E-12	Glioma
3768	-2.042782879	6.98E-19	Glioma
6000	-2.038653934	2.43E-20	Glioma
150147	-2.037872648	1.10E-15	Glioma
50614	-2.037270952	3.31E-13	Glioma
121601	-2.03467379	1.93E-21	Glioma
389332	-2.031931012	2.97E-15	Glioma
90249	-2.031010999	2.67E-20	Glioma
161357	-2.030237103	6.57E-14	Glioma
148198	-2.021965566	3.61E-19	Glioma
80852	-2.019348259	2.15E-17	Glioma
80059	-2.017922067	1.29E-16	Glioma
256130	-2.017827257	3.69E-11	Glioma
57626	-2.017237632	5.01E-17	Glioma
27133	-2.013732174	2.47E-14	Glioma
100038246	-2.011612499	5.44E-14	Glioma
27319	-2.007394278	2.55E-13	Glioma
83873	-2.004504881	1.80E-20	Glioma
22895	-2.00370608	6.26E-12	Glioma
64901	-2.002411398	1.31E-13	Glioma
794	-1.999460921	1.27E-11	Glioma
386618	-1.998564212	8.66E-21	Glioma
64388	-1.997739543	8.95E-15	Glioma
5593	-1.997245943	6.47E-13	Glioma
83851	-1.993750537	1.37E-17	Glioma
401190	-1.992713735	3.19E-20	Glioma
23101	-1.991424296	1.49E-19	Glioma
57687	-1.9913026	1.91E-17	Glioma
57578	-1.990532933	4.12E-22	Glioma
2662	-1.987205687	3.32E-11	Glioma
288	-1.980917865	1.90E-23	Glioma
29091	-1.97571051	6.46E-27	Glioma
11122	-1.974090044	2.15E-12	Glioma
5579	-1.971765015	7.28E-21	Glioma
64405	-1.971585135	7.15E-15	Glioma

84870	-1.970027377	1.78E-14	Glioma
3736	-1.968623658	6.28E-13	Glioma
286499	-1.968139963	1.02E-16	Glioma
1961	-1.964480613	3.31E-12	Glioma
79698	-1.961848531	6.17E-15	Glioma
83546	-1.959446286	1.25E-16	Glioma
27328	-1.959165563	8.72E-11	Glioma
114757	-1.959065681	2.22E-25	Glioma
27330	-1.958980415	3.46E-15	Glioma
10739	-1.956310789	1.82E-17	Glioma
140767	-1.950615826	1.11E-20	Glioma
408263	-1.946556431	3.87E-14	Glioma
94160	-1.943279331	2.48E-14	Glioma
793	-1.941979674	2.24E-13	Glioma
151126	-1.940058972	2.32E-14	Glioma
148808	-1.939763498	5.39E-28	Glioma
79955	-1.935931931	1.64E-25	Glioma
83698	-1.935192751	7.44E-11	Glioma
203859	-1.934552712	9.74E-20	Glioma
114798	-1.934402607	1.58E-14	Glioma
90113	-1.933613693	3.09E-19	Glioma
79948	-1.929040402	1.99E-10	Glioma
10815	-1.927670248	5.91E-19	Glioma
1010	-1.927359168	2.16E-19	Glioma
57554	-1.927034821	3.21E-14	Glioma
729993	-1.926881244	2.54E-16	Glioma
6861	-1.923377262	2.19E-15	Glioma
401498	-1.920951677	7.71E-15	Glioma
6334	-1.917504467	1.43E-22	Glioma
10231	-1.916274179	7.62E-25	Glioma
152789	-1.915553735	2.23E-16	Glioma
340554	-1.913647863	3.56E-17	Glioma
9956	-1.913208952	6.83E-15	Glioma
5368	-1.912465711	5.14E-09	Glioma
339390	-1.911435187	7.28E-17	Glioma
8988	-1.911408576	1.60E-14	Glioma
57451	-1.907021511	1.06E-13	Glioma
5923	-1.904781123	6.16E-12	Glioma
956	-1.904779542	2.00E-10	Glioma
90134	-1.903524494	5.37E-12	Glioma
93429	-1.902555193	7.47E-12	Glioma
441666	-1.902177667	2.53E-13	Glioma
7143	-1.900866218	4.08E-09	Glioma
5649	-1.899011558	1.25E-11	Glioma
5949	-1.896749547	6.44E-17	Glioma
145581	-1.892171445	2.53E-11	Glioma
285220	-1.891466241	1.02E-14	Glioma
26468	-1.890378309	1.14E-15	Glioma
59283	-1.889671224	5.88E-18	Glioma
80737	-1.886464369	1.93E-14	Glioma
56479	-1.884797499	5.18E-15	Glioma
222662	-1.884122183	3.29E-19	Glioma
148014	-1.883851322	4.71E-17	Glioma
51725	-1.880019221	4.89E-15	Glioma
3798	-1.879994041	1.05E-19	Glioma
4293	-1.879410203	6.10E-17	Glioma
6622	-1.878098681	1.64E-22	Glioma
139065	-1.877813868	2.75E-12	Glioma

4488	-1.87596494	3.06E-13	Glioma
6750	-1.875945601	1.88E-09	Glioma
6866	-1.872277071	2.90E-09	Glioma
147372	-1.867152543	1.18E-18	Glioma
85300	-1.866351617	4.00E-12	Glioma
5318	-1.86320408	2.03E-16	Glioma
2844	-1.862241086	8.34E-19	Glioma
9369	-1.853825032	1.32E-17	Glioma
64881	-1.851580336	3.08E-14	Glioma
79369	-1.849294395	8.67E-16	Glioma
2046	-1.847796629	4.43E-16	Glioma
26470	-1.847123692	4.85E-25	Glioma
284485	-1.84637537	1.58E-22	Glioma
140689	-1.845447264	6.04E-08	Glioma
285596	-1.841856269	2.55E-14	Glioma
8174	-1.840691918	1.11E-17	Glioma
57611	-1.8398017	2.23E-13	Glioma
171177	-1.839725872	1.47E-18	Glioma
219539	-1.83805525	6.13E-26	Glioma
6812	-1.837080448	1.13E-33	Glioma
84502	-1.833924464	2.98E-16	Glioma
9177	-1.831760632	1.24E-15	Glioma
1310	-1.83143946	3.34E-18	Glioma
64137	-1.829789666	3.85E-18	Glioma
1129	-1.828913766	7.73E-18	Glioma
1747	-1.827491696	3.10E-21	Glioma
8927	-1.826715346	1.15E-22	Glioma
9317	-1.825449646	3.61E-14	Glioma
10900	-1.821243245	2.49E-26	Glioma
93377	-1.820510283	1.86E-05	Glioma
54039	-1.820045664	1.67E-20	Glioma
84856	-1.819626719	5.76E-11	Glioma
285175	-1.818947138	2.90E-30	Glioma
644596	-1.81827716	1.87E-25	Glioma
63908	-1.818225196	1.30E-23	Glioma
116369	-1.818117886	1.26E-16	Glioma
10842	-1.817452006	3.09E-16	Glioma
84940	-1.814188876	9.05E-16	Glioma
57528	-1.814147126	3.56E-24	Glioma
29114	-1.81356321	1.28E-19	Glioma
84302	-1.80965187	4.69E-20	Glioma
646113	-1.80653946	1.60E-11	Glioma
340745	-1.806479448	4.91E-18	Glioma
1392	-1.802198975	2.31E-12	Glioma
7306	-1.799538712	2.55E-15	Glioma
58512	-1.79888051	2.62E-17	Glioma
143379	-1.79660178	1.58E-18	Glioma
3775	-1.795774333	1.18E-14	Glioma
261729	-1.794895767	3.74E-21	Glioma
128611	-1.794490787	9.73E-14	Glioma
6252	-1.793587052	1.32E-24	Glioma
170261	-1.792158569	2.06E-13	Glioma
158228	-1.790360531	1.69E-13	Glioma
440435	-1.789021778	3.94E-17	Glioma
442319	-1.787256736	8.28E-13	Glioma
26289	-1.78634254	3.39E-10	Glioma
389197	-1.785175696	2.91E-14	Glioma
5733	-1.784952536	1.76E-11	Glioma

23025	-1.781699397	5.15E-20	Glioma
113220	-1.779727562	3.30E-15	Glioma
59344	-1.779595548	7.93E-20	Glioma
150379	-1.77788215	6.23E-17	Glioma
81033	-1.77602281	1.14E-20	Glioma
9348	-1.775854704	3.95E-09	Glioma
5646	-1.774857347	1.82E-15	Glioma
1137	-1.774011693	9.41E-12	Glioma
9108	-1.771642268	1.01E-29	Glioma
6710	-1.771074823	5.79E-18	Glioma
118738	-1.770682885	4.33E-12	Glioma
6014	-1.76790293	2.86E-10	Glioma
25818	-1.767307862	2.11E-17	Glioma
114805	-1.765766265	4.76E-10	Glioma
5737	-1.764945572	4.41E-13	Glioma
122402	-1.762956583	6.91E-17	Glioma
1132	-1.756489691	1.01E-19	Glioma
9340	-1.755916094	3.48E-15	Glioma
5067	-1.754837501	3.31E-08	Glioma
1006	-1.75373248	7.39E-11	Glioma
1149	-1.752135097	1.65E-14	Glioma
134829	-1.751553194	3.90E-09	Glioma
273	-1.749968129	6.17E-26	Glioma
170850	-1.749929127	7.30E-14	Glioma
51305	-1.748076658	1.10E-10	Glioma
478	-1.747034797	6.89E-17	Glioma
134111	-1.746188338	8.54E-18	Glioma
6869	-1.746159655	5.83E-11	Glioma
100133669	-1.746057414	4.46E-22	Glioma
374383	-1.743572727	1.25E-20	Glioma
2567	-1.74349736	2.23E-16	Glioma
6855	-1.743385783	6.82E-28	Glioma
3745	-1.741911869	4.10E-16	Glioma
283731	-1.736915679	1.66E-13	Glioma
9066	-1.736425333	1.80E-13	Glioma
11076	-1.736424343	1.74E-17	Glioma
56143	-1.735128563	1.60E-17	Glioma
9356	-1.73345694	1.32E-09	Glioma
534	-1.732254065	7.77E-29	Glioma
286411	-1.730572494	9.88E-17	Glioma
112755	-1.725405715	5.90E-21	Glioma
2039	-1.725358372	1.55E-20	Glioma
1852	-1.725049841	7.42E-13	Glioma
728603	-1.72085974	6.27E-15	Glioma
5274	-1.720187898	1.65E-16	Glioma
777	-1.7167385	1.77E-15	Glioma
146330	-1.714118163	2.78E-19	Glioma
5995	-1.713406001	1.69E-11	Glioma
105	-1.712350062	1.90E-11	Glioma
387890	-1.711490522	1.41E-12	Glioma
148930	-1.711376909	9.75E-13	Glioma
26051	-1.71026146	4.37E-18	Glioma
56924	-1.708514072	7.17E-17	Glioma
253982	-1.708337036	9.55E-27	Glioma
1141	-1.705693747	1.56E-18	Glioma
26222	-1.704436723	1.69E-19	Glioma
134548	-1.702544241	6.37E-15	Glioma
284111	-1.701252965	2.31E-07	Glioma

222183	-1.698983061	3.39E-17	Glioma
26095	-1.698864959	7.36E-13	Glioma
11141	-1.698323642	8.92E-17	Glioma
306	-1.696749475	4.57E-13	Glioma
257194	-1.695224658	4.55E-15	Glioma
2550	-1.689617191	1.06E-23	Glioma
9254	-1.686054163	2.81E-17	Glioma
83850	-1.685941579	3.42E-17	Glioma
7757	-1.68524019	1.63E-10	Glioma
342865	-1.684960017	1.59E-11	Glioma
81849	-1.684044281	2.91E-09	Glioma
2172	-1.681869063	9.39E-12	Glioma
168391	-1.67838974	8.04E-17	Glioma
29944	-1.677995075	1.72E-14	Glioma
10279	-1.676766203	4.17E-12	Glioma
282973	-1.671384385	3.66E-19	Glioma
51555	-1.66936521	6.03E-12	Glioma
85508	-1.667562143	1.78E-08	Glioma
2134	-1.666239267	5.17E-23	Glioma
10777	-1.66507633	4.54E-16	Glioma
10202	-1.664836137	6.52E-11	Glioma
8224	-1.664659008	1.14E-16	Glioma
25849	-1.664405996	1.26E-19	Glioma
56978	-1.663549772	9.25E-14	Glioma
139221	-1.663194758	1.88E-11	Glioma
3131	-1.663110287	1.65E-27	Glioma
57221	-1.661414405	2.57E-15	Glioma
8447	-1.661168637	4.15E-13	Glioma
145282	-1.659720363	1.36E-12	Glioma
257068	-1.658343711	3.15E-22	Glioma
1038	-1.657493726	1.42E-11	Glioma
378465	-1.656081403	1.50E-17	Glioma
4145	-1.653562079	3.44E-13	Glioma
4353	-1.652490139	7.00E-14	Glioma
340419	-1.650286149	2.50E-10	Glioma
54716	-1.649116638	6.46E-12	Glioma
54566	-1.649103556	8.52E-16	Glioma
401474	-1.64865805	1.71E-21	Glioma
4889	-1.648164843	3.45E-12	Glioma
259217	-1.645595703	9.75E-26	Glioma
340533	-1.644920698	1.60E-15	Glioma
60680	-1.642152788	6.45E-12	Glioma
5053	-1.639784027	6.30E-15	Glioma
56884	-1.636932825	1.04E-07	Glioma
79057	-1.636177802	3.22E-13	Glioma
54894	-1.635498282	8.09E-14	Glioma
125113	-1.63544273	3.06E-33	Glioma
8999	-1.635187795	1.41E-11	Glioma
100130238	-1.634689394	2.88E-13	Glioma
338651	-1.633181957	1.05E-12	Glioma
4072	-1.632791617	2.09E-17	Glioma
3595	-1.631839218	1.95E-12	Glioma
6547	-1.631607624	2.95E-13	Glioma
143425	-1.630411901	2.16E-13	Glioma
64579	-1.628089539	3.07E-09	Glioma
3036	-1.62808542	2.60E-11	Glioma
25769	-1.627855348	1.11E-10	Glioma
1996	-1.626704394	2.24E-11	Glioma

5961	-1.622502154	1.90E-12	Glioma
202134	-1.622034396	3.39E-13	Glioma
136227	-1.620637377	8.92E-10	Glioma
25791	-1.620001527	6.76E-11	Glioma
347745	-1.619794388	3.07E-21	Glioma
150726	-1.619332548	1.73E-27	Glioma
339398	-1.618050081	2.31E-16	Glioma
56899	-1.617305524	6.53E-18	Glioma
55966	-1.617253396	5.01E-17	Glioma
128434	-1.61707481	5.53E-12	Glioma
9645	-1.615687604	1.04E-15	Glioma
9331	-1.615153493	4.32E-29	Glioma
283514	-1.612694653	7.77E-12	Glioma
79762	-1.612314944	8.22E-17	Glioma
4160	-1.611363363	2.42E-14	Glioma
3767	-1.60971854	1.25E-15	Glioma
57644	-1.607732646	4.66E-15	Glioma
223117	-1.607407296	3.46E-08	Glioma
6792	-1.605163282	2.64E-10	Glioma
1543	-1.604468837	1.70E-19	Glioma
9495	-1.603581564	1.72E-20	Glioma
57692	-1.602416104	4.35E-24	Glioma
6247	-1.601501566	4.91E-13	Glioma
114800	-1.598982296	4.43E-15	Glioma
57156	-1.597340273	1.31E-27	Glioma
9353	-1.596456595	1.16E-10	Glioma
145773	-1.593975615	2.25E-20	Glioma
22891	-1.592974894	8.26E-30	Glioma
51127	-1.592716231	2.03E-18	Glioma
171019	-1.591444203	3.17E-12	Glioma
91851	-1.590274636	8.19E-12	Glioma
6505	-1.589958775	1.54E-24	Glioma
3290	-1.589785518	3.33E-11	Glioma
26052	-1.589448914	9.70E-22	Glioma
10396	-1.586988499	1.28E-20	Glioma
8514	-1.585861249	5.77E-19	Glioma
22987	-1.585163763	2.04E-11	Glioma
10242	-1.585066207	3.82E-19	Glioma
7277	-1.582353544	8.35E-16	Glioma
145837	-1.579806357	1.36E-19	Glioma
340578	-1.579549872	1.03E-11	Glioma
5136	-1.57930983	8.43E-14	Glioma
11249	-1.578888306	1.03E-09	Glioma
57662	-1.57823417	7.99E-10	Glioma
3360	-1.578057277	8.28E-12	Glioma
5924	-1.574462289	4.52E-16	Glioma
57497	-1.573129478	4.29E-11	Glioma
140733	-1.572887249	2.40E-16	Glioma
139728	-1.572597611	9.12E-17	Glioma
1759	-1.571219045	1.78E-11	Glioma
22874	-1.56706408	3.66E-26	Glioma
2250	-1.566014076	5.95E-20	Glioma
4185	-1.565593649	2.61E-14	Glioma
5122	-1.562902091	4.42E-10	Glioma
56675	-1.562778117	3.64E-13	Glioma
2051	-1.562438668	4.25E-10	Glioma
23285	-1.561845883	2.04E-26	Glioma
6804	-1.560606799	9.22E-15	Glioma

10409	-1.559306192	6.16E-22	Glioma
3084	-1.558685275	4.45E-09	Glioma
54550	-1.556188198	5.79E-17	Glioma
2322	-1.555129614	5.56E-14	Glioma
51286	-1.553643867	1.14E-32	Glioma
768206	-1.551902737	1.86E-23	Glioma
55079	-1.551879078	8.10E-10	Glioma
92211	-1.551207781	3.75E-15	Glioma
10439	-1.548164459	3.87E-17	Glioma
56660	-1.547587064	2.29E-12	Glioma
10021	-1.546917423	4.03E-11	Glioma
4311	-1.544101531	1.57E-11	Glioma
165257	-1.543538536	4.93E-09	Glioma
154197	-1.542052536	2.01E-13	Glioma
255928	-1.540941402	4.07E-16	Glioma
100188954	-1.540495899	7.23E-10	Glioma
1123	-1.538508735	6.53E-21	Glioma
3746	-1.538028233	6.10E-19	Glioma
57562	-1.536827878	6.82E-22	Glioma
2835	-1.534454763	9.12E-09	Glioma
100131897	-1.534402391	4.40E-10	Glioma
8526	-1.532102634	1.96E-18	Glioma
23017	-1.531305268	1.67E-27	Glioma
84618	-1.530788634	3.96E-11	Glioma
6695	-1.530518015	3.66E-18	Glioma
140886	-1.53046187	1.44E-10	Glioma
80725	-1.52912833	5.15E-16	Glioma
2254	-1.525638113	1.92E-10	Glioma
3872	-1.521590607	2.26E-07	Glioma
114134	-1.520414352	1.55E-25	Glioma
57476	-1.520226073	1.94E-23	Glioma
57628	-1.51962388	1.67E-10	Glioma
85411	-1.518937466	4.91E-27	Glioma
139599	-1.51892194	8.22E-09	Glioma
53942	-1.518906801	1.30E-08	Glioma
112609	-1.518808828	3.22E-09	Glioma
4978	-1.518291129	1.64E-12	Glioma
84440	-1.516708301	4.11E-20	Glioma
401647	-1.516102378	9.01E-18	Glioma
60495	-1.516065494	7.93E-06	Glioma
134701	-1.51581609	2.32E-16	Glioma
84830	-1.515558283	2.38E-11	Glioma
349667	-1.513998804	4.14E-17	Glioma
59335	-1.513513637	1.09E-17	Glioma
7433	-1.511797461	3.72E-15	Glioma
83394	-1.511606329	1.61E-14	Glioma
340542	-1.511455114	9.83E-12	Glioma
57524	-1.511178723	1.13E-18	Glioma
26287	-1.507585101	1.35E-12	Glioma
5581	-1.507475183	1.40E-29	Glioma
78997	-1.50463065	9.77E-15	Glioma
6712	-1.503926421	3.15E-18	Glioma
1395	-1.503752984	2.79E-14	Glioma
65989	-1.503121658	3.80E-16	Glioma
2028	1.500282226	2.75E-15	Glioma
5457	1.50100239	2.12E-07	Glioma
5888	1.501080101	4.73E-18	Glioma
8673	1.501118217	9.65E-27	Glioma

54436	1.50129414	5.99E-32	Glioma
283897	1.501720693	2.45E-25	Glioma
1278	1.501772142	2.21E-15	Glioma
55388	1.503809724	1.87E-10	Glioma
221091	1.503957102	2.74E-10	Glioma
147912	1.504265863	1.29E-31	Glioma
10451	1.508844627	3.95E-08	Glioma
1441	1.50991602	1.84E-27	Glioma
80122	1.510807017	2.12E-07	Glioma
7132	1.51092217	1.65E-30	Glioma
245806	1.512052573	3.57E-11	Glioma
3099	1.512890741	1.24E-24	Glioma
4288	1.514484177	1.74E-10	Glioma
79801	1.51792727	1.02E-20	Glioma
684	1.519486209	6.71E-22	Glioma
64926	1.51964653	8.11E-29	Glioma
56956	1.520277697	1.88E-07	Glioma
401124	1.520695393	1.96E-11	Glioma
6672	1.522309822	1.69E-29	Glioma
51279	1.523994393	3.03E-21	Glioma
54809	1.525675973	7.95E-25	Glioma
286204	1.526398947	4.09E-13	Glioma
5788	1.526511572	4.91E-24	Glioma
10261	1.52726294	8.76E-29	Glioma
112597	1.527442452	3.18E-11	Glioma
55603	1.527913472	1.65E-31	Glioma
3690	1.528427517	6.53E-16	Glioma
9047	1.528496832	2.53E-16	Glioma
115761	1.528577549	1.14E-28	Glioma
83690	1.528786401	1.04E-28	Glioma
83540	1.529696873	1.23E-16	Glioma
11173	1.53255371	5.69E-19	Glioma
915	1.533550589	6.97E-12	Glioma
221883	1.534748145	5.07E-11	Glioma
2787	1.535010801	4.48E-33	Glioma
1063	1.536030866	3.59E-15	Glioma
945	1.536728684	8.85E-26	Glioma
50619	1.537414192	3.12E-32	Glioma
10981	1.538066653	1.80E-25	Glioma
57082	1.538089883	2.19E-10	Glioma
128344	1.538554575	2.60E-16	Glioma
710	1.539202406	1.71E-17	Glioma
55365	1.539236786	9.04E-17	Glioma
5031	1.540018096	1.23E-20	Glioma
3486	1.540240516	1.19E-11	Glioma
259307	1.540802394	1.67E-29	Glioma
3232	1.542196852	5.41E-07	Glioma
1674	1.543074105	1.58E-05	Glioma
1230	1.543552701	3.95E-25	Glioma
11339	1.544430938	2.53E-18	Glioma
7083	1.544470323	4.88E-18	Glioma
84166	1.544498281	8.20E-38	Glioma
7852	1.545445652	1.62E-27	Glioma
10870	1.546145321	3.80E-23	Glioma
942	1.546427065	1.00E-25	Glioma
55789	1.547145998	3.19E-17	Glioma
3209	1.547167738	5.98E-09	Glioma
339768	1.547409873	2.24E-11	Glioma

157570	1.549200932	1.16E-12	Glioma
11314	1.549451982	3.21E-29	Glioma
3586	1.549516378	5.24E-13	Glioma
924	1.549692931	2.10E-20	Glioma
3594	1.55295152	4.18E-26	Glioma
3702	1.55315265	7.43E-12	Glioma
963	1.553335852	6.52E-29	Glioma
1520	1.553540007	7.96E-28	Glioma
5347	1.553659545	7.74E-26	Glioma
9111	1.554086835	2.98E-32	Glioma
3001	1.554502652	8.42E-11	Glioma
83938	1.554711668	4.87E-22	Glioma
3561	1.556011791	4.39E-21	Glioma
25937	1.557707114	2.34E-22	Glioma
284759	1.557956864	2.09E-26	Glioma
51296	1.558634407	6.22E-37	Glioma
50507	1.558702838	5.75E-12	Glioma
5008	1.558722332	3.24E-11	Glioma
2669	1.558798052	9.60E-21	Glioma
283	1.559155287	1.32E-21	Glioma
864	1.55954473	3.28E-27	Glioma
128153	1.559931907	3.27E-14	Glioma
3162	1.559959598	4.01E-23	Glioma
2825	1.559974682	1.89E-10	Glioma
11035	1.560495988	2.33E-33	Glioma
10561	1.561091037	1.53E-30	Glioma
80183	1.561326168	7.84E-20	Glioma
841	1.561380376	6.10E-34	Glioma
140	1.561746959	2.42E-25	Glioma
10663	1.562949763	3.30E-12	Glioma
64092	1.563471669	1.37E-25	Glioma
6518	1.563735466	2.67E-21	Glioma
348013	1.563865362	2.92E-22	Glioma
79168	1.564146965	7.81E-17	Glioma
6280	1.565014699	9.86E-10	Glioma
5224	1.566593264	2.24E-18	Glioma
51338	1.567424274	1.89E-20	Glioma
64170	1.567859108	1.72E-30	Glioma
22797	1.569117896	1.08E-29	Glioma
80008	1.572168473	4.09E-23	Glioma
28959	1.572548466	5.41E-19	Glioma
7409	1.573787444	3.65E-29	Glioma
11184	1.574208439	1.19E-32	Glioma
1806	1.574708023	3.90E-19	Glioma
5724	1.575013412	8.84E-22	Glioma
706	1.575321599	1.18E-28	Glioma
128346	1.576127104	9.54E-26	Glioma
9156	1.577887858	4.55E-13	Glioma
57121	1.578623875	1.35E-26	Glioma
2305	1.578680219	1.27E-14	Glioma
4600	1.578868797	2.31E-16	Glioma
1050	1.57998817	2.48E-30	Glioma
4069	1.580021599	1.18E-12	Glioma
3127	1.581623376	6.16E-13	Glioma
10954	1.582502634	4.70E-28	Glioma
10677	1.58277392	3.64E-20	Glioma
23533	1.582923039	3.71E-33	Glioma
100130776	1.582964657	6.05E-13	Glioma

11185	1.586218875	6.59E-12	Glioma
8701	1.587088617	9.63E-10	Glioma
57705	1.588526914	7.85E-28	Glioma
3059	1.588647364	1.14E-31	Glioma
347454	1.589419651	7.21E-26	Glioma
3833	1.590597078	4.83E-14	Glioma
341640	1.592036034	1.58E-11	Glioma
1535	1.592952309	5.78E-35	Glioma
1292	1.593267192	1.22E-13	Glioma
5820	1.593388526	8.61E-16	Glioma
122618	1.59409727	5.43E-20	Glioma
80342	1.59453989	2.78E-24	Glioma
362	1.594655085	4.74E-06	Glioma
6347	1.59467534	4.09E-11	Glioma
3620	1.596357557	1.20E-10	Glioma
29015	1.598301158	4.40E-21	Glioma
3569	1.599342878	1.28E-09	Glioma
8463	1.599829266	1.13E-26	Glioma
4542	1.602797725	3.54E-37	Glioma
94240	1.602940665	1.77E-20	Glioma
6693	1.603281262	2.34E-34	Glioma
22798	1.603836618	2.86E-23	Glioma
701	1.604467761	7.38E-12	Glioma
5359	1.609015644	2.07E-27	Glioma
6004	1.609340473	1.26E-17	Glioma
3426	1.610045937	5.86E-16	Glioma
146850	1.611314206	1.56E-30	Glioma
89857	1.611386513	2.72E-32	Glioma
3071	1.612048329	2.39E-32	Glioma
8091	1.612109712	7.85E-10	Glioma
27071	1.612232752	3.42E-23	Glioma
7351	1.612756696	1.05E-31	Glioma
1945	1.612865094	4.77E-30	Glioma
10457	1.614799469	6.74E-15	Glioma
51203	1.615109549	8.99E-15	Glioma
720	1.615116713	1.15E-18	Glioma
83903	1.61696042	1.02E-17	Glioma
55509	1.618023726	3.79E-20	Glioma
9398	1.61848998	4.35E-24	Glioma
84467	1.619149395	9.10E-10	Glioma
29108	1.619933389	2.28E-31	Glioma
712	1.620469699	1.31E-26	Glioma
8832	1.620978411	6.28E-24	Glioma
303	1.621995724	4.68E-16	Glioma
84962	1.622030214	3.40E-33	Glioma
27350	1.622853378	4.57E-26	Glioma
695	1.623076546	4.95E-32	Glioma
10855	1.623657736	9.70E-21	Glioma
7805	1.624432349	5.81E-32	Glioma
9227	1.625102459	1.89E-11	Glioma
162517	1.625354011	1.12E-13	Glioma
7462	1.625708624	4.94E-31	Glioma
84034	1.625848211	2.19E-17	Glioma
100124700	1.626559927	3.22E-11	Glioma
6510	1.627347074	1.60E-29	Glioma
654816	1.627625029	1.02E-23	Glioma
9636	1.628097216	5.39E-17	Glioma
5148	1.62903354	1.22E-24	Glioma

283592	1.630444964	4.98E-13	Glioma
2637	1.630919096	8.50E-11	Glioma
55214	1.631390934	5.64E-23	Glioma
121506	1.632362863	1.77E-16	Glioma
430	1.632809784	9.77E-25	Glioma
4478	1.633151468	6.35E-27	Glioma
7305	1.633314961	7.04E-30	Glioma
3965	1.633888542	1.27E-36	Glioma
84617	1.635926065	6.54E-30	Glioma
1594	1.637488848	4.63E-15	Glioma
3665	1.637961654	3.87E-32	Glioma
968	1.638212629	4.29E-33	Glioma
7130	1.638568296	1.47E-09	Glioma
80310	1.640291275	6.57E-11	Glioma
3108	1.64183813	1.45E-27	Glioma
10232	1.64302144	3.72E-09	Glioma
116496	1.644378212	9.54E-24	Glioma
83706	1.64634724	7.93E-41	Glioma
389799	1.646370043	1.08E-10	Glioma
3687	1.64732105	1.24E-30	Glioma
79682	1.648972346	2.35E-18	Glioma
30817	1.649438048	7.89E-29	Glioma
79819	1.649950172	1.51E-21	Glioma
64105	1.650951904	7.53E-14	Glioma
64581	1.651876227	2.63E-22	Glioma
2331	1.652698147	2.74E-09	Glioma
54518	1.654656616	1.89E-28	Glioma
340205	1.655420895	7.10E-22	Glioma
8876	1.655958697	6.96E-19	Glioma
54504	1.658353264	6.45E-25	Glioma
55911	1.658698741	3.20E-41	Glioma
11024	1.65898275	1.23E-21	Glioma
4046	1.660189946	4.73E-16	Glioma
84419	1.660604489	2.81E-12	Glioma
79998	1.66259325	1.96E-20	Glioma
55970	1.663068286	7.12E-21	Glioma
1062	1.663604019	6.17E-19	Glioma
4938	1.664245911	4.35E-19	Glioma
54558	1.664580552	2.71E-17	Glioma
23682	1.664765531	4.60E-22	Glioma
4828	1.664814404	6.01E-17	Glioma
51311	1.667839906	9.18E-18	Glioma
165631	1.669479451	3.33E-22	Glioma
25903	1.670826184	1.54E-24	Glioma
944	1.673325179	2.34E-23	Glioma
29774	1.673826493	1.03E-20	Glioma
7042	1.674658179	1.00E-16	Glioma
1536	1.674671596	7.43E-28	Glioma
9595	1.675812817	1.14E-26	Glioma
8651	1.678086812	6.51E-23	Glioma
56944	1.678537334	3.29E-36	Glioma
201799	1.67937908	2.78E-26	Glioma
8778	1.683257848	3.18E-32	Glioma
25790	1.683500068	1.26E-10	Glioma
81031	1.686554087	8.92E-20	Glioma
890	1.691806576	8.96E-21	Glioma
23237	1.692846397	1.40E-19	Glioma
9052	1.693764956	1.93E-10	Glioma

150468	1.693795883	4.31E-13	Glioma
4017	1.69514252	6.19E-25	Glioma
3310	1.695364793	6.94E-16	Glioma
54507	1.697115033	3.46E-29	Glioma
219855	1.698922991	1.74E-33	Glioma
414236	1.699024485	2.00E-21	Glioma
79841	1.699993229	4.52E-16	Glioma
3606	1.702487805	2.62E-28	Glioma
60489	1.703879089	2.74E-27	Glioma
126393	1.704972062	1.08E-16	Glioma
2191	1.705844784	1.16E-13	Glioma
929	1.705874504	5.67E-25	Glioma
154664	1.706600103	8.79E-13	Glioma
94031	1.709264455	1.78E-14	Glioma
9582	1.70935807	1.83E-16	Glioma
338773	1.71204199	9.67E-22	Glioma
56911	1.714870191	2.50E-16	Glioma
440738	1.715753779	2.50E-10	Glioma
84868	1.71579677	1.15E-32	Glioma
84002	1.716246187	1.90E-26	Glioma
81622	1.717073386	2.63E-38	Glioma
347735	1.717859818	6.79E-19	Glioma
3113	1.718847902	9.40E-19	Glioma
714	1.719601192	8.59E-32	Glioma
9768	1.720382999	1.18E-13	Glioma
240	1.7209302	7.11E-29	Glioma
3399	1.721539684	4.65E-29	Glioma
9333	1.725761382	1.13E-12	Glioma
24137	1.725774968	1.52E-16	Glioma
400823	1.727342647	7.64E-18	Glioma
100130613	1.728176857	4.11E-14	Glioma
8835	1.729388716	4.55E-20	Glioma
6688	1.732833512	1.50E-37	Glioma
7004	1.733421185	1.82E-22	Glioma
10537	1.734041831	4.44E-14	Glioma
728	1.735709496	6.01E-22	Glioma
9021	1.736955785	1.77E-12	Glioma
84790	1.738068122	1.59E-20	Glioma
9056	1.738588051	1.18E-36	Glioma
79887	1.740993814	3.97E-22	Glioma
54440	1.741489562	7.42E-34	Glioma
374393	1.741965936	2.93E-13	Glioma
916	1.745276817	9.61E-14	Glioma
2213	1.747245652	1.11E-09	Glioma
951	1.747568055	7.73E-31	Glioma
64098	1.749003515	1.65E-38	Glioma
284403	1.749378618	1.45E-22	Glioma
399	1.749410581	1.65E-25	Glioma
55	1.749678246	2.50E-15	Glioma
9077	1.750121953	3.55E-16	Glioma
6769	1.754172502	1.75E-07	Glioma
2000	1.754195077	1.41E-36	Glioma
3903	1.754733322	3.38E-32	Glioma
3123	1.757856238	7.30E-19	Glioma
5329	1.757990242	1.51E-24	Glioma
343099	1.758423837	9.28E-38	Glioma
5836	1.758968624	1.66E-33	Glioma
7035	1.759341553	8.81E-19	Glioma

3115	1.759641524	1.83E-20	Glioma
3101	1.764681063	1.50E-21	Glioma
7136	1.766696868	1.51E-21	Glioma
90332	1.767241919	3.07E-13	Glioma
93349	1.768204378	6.56E-37	Glioma
6037	1.769166973	2.12E-17	Glioma
100128927	1.770030748	2.81E-36	Glioma
100049587	1.770071055	1.40E-21	Glioma
126364	1.770472134	1.19E-31	Glioma
128239	1.770993865	5.00E-17	Glioma
220134	1.771589372	5.23E-17	Glioma
144455	1.77291907	1.27E-13	Glioma
6288	1.773656541	1.14E-07	Glioma
965	1.774820959	3.77E-26	Glioma
1903	1.774953536	1.09E-30	Glioma
55872	1.775119043	1.88E-11	Glioma
10234	1.776688	3.99E-26	Glioma
3234	1.778142128	2.69E-08	Glioma
6281	1.778224388	6.19E-19	Glioma
6398	1.779520791	7.20E-19	Glioma
1154	1.780925843	8.12E-27	Glioma
285386	1.781160547	1.13E-24	Glioma
9928	1.784767365	2.85E-17	Glioma
4267	1.786178959	1.76E-23	Glioma
348174	1.786179618	5.43E-24	Glioma
2209	1.787691512	8.75E-24	Glioma
51225	1.789812227	2.49E-41	Glioma
391059	1.790458652	7.43E-19	Glioma
51512	1.791431482	2.37E-17	Glioma
4064	1.791967594	8.72E-34	Glioma
645367	1.792495128	9.30E-15	Glioma
10673	1.793843183	8.74E-16	Glioma
699	1.794298857	1.48E-15	Glioma
150771	1.794965148	2.57E-22	Glioma
1104	1.796715975	7.23E-17	Glioma
713	1.797914006	1.21E-30	Glioma
11326	1.798135101	1.14E-24	Glioma
9940	1.798616541	1.25E-18	Glioma
56892	1.799663456	2.30E-23	Glioma
4982	1.799968316	2.01E-12	Glioma
169792	1.801159494	4.09E-30	Glioma
1842	1.803259978	1.23E-23	Glioma
64866	1.805016724	4.77E-27	Glioma
5133	1.805076617	1.62E-20	Glioma
837	1.806354367	1.56E-29	Glioma
1261	1.806790836	8.67E-13	Glioma
434	1.809635166	5.76E-20	Glioma
8438	1.809777125	5.95E-26	Glioma
2700	1.809961974	6.72E-12	Glioma
8638	1.810275566	1.16E-15	Glioma
4316	1.811013663	1.54E-09	Glioma
5251	1.812414765	1.59E-17	Glioma
79924	1.812801187	1.52E-20	Glioma
3557	1.81397468	1.82E-13	Glioma
10871	1.814981421	2.88E-30	Glioma
10859	1.815328223	1.61E-31	Glioma
133923	1.815938228	8.60E-19	Glioma
29842	1.816252672	4.86E-12	Glioma

11027	1.820799666	1.03E-30	Glioma
160365	1.822989477	4.95E-21	Glioma
56833	1.823923073	1.32E-25	Glioma
3109	1.824612056	9.15E-29	Glioma
55504	1.825340525	8.89E-32	Glioma
158158	1.825685948	1.58E-13	Glioma
170689	1.826065081	1.07E-15	Glioma
6273	1.827302806	1.82E-21	Glioma
644139	1.830739308	2.14E-12	Glioma
219285	1.830951636	4.52E-32	Glioma
401551	1.831142251	3.55E-10	Glioma
11006	1.832569804	3.51E-34	Glioma
2635	1.833890698	7.14E-20	Glioma
5880	1.834592778	7.93E-34	Glioma
285834	1.834926773	5.05E-15	Glioma
2212	1.835099986	1.35E-30	Glioma
1192	1.836531023	3.54E-28	Glioma
3205	1.839127408	2.52E-11	Glioma
7272	1.839835256	3.63E-14	Glioma
57010	1.840641903	2.37E-28	Glioma
1052	1.842022069	1.87E-27	Glioma
27074	1.842186513	1.66E-20	Glioma
51191	1.842328719	3.55E-25	Glioma
9123	1.845129541	1.36E-35	Glioma
358	1.848045994	8.43E-12	Glioma
9674	1.848380173	9.95E-26	Glioma
7045	1.848992729	4.00E-19	Glioma
147841	1.849831978	1.60E-14	Glioma
2207	1.850225126	4.35E-33	Glioma
2625	1.851163537	1.29E-18	Glioma
1295	1.851792362	4.62E-10	Glioma
54502	1.85349649	8.75E-33	Glioma
302	1.855860048	1.52E-22	Glioma
64005	1.858371426	9.66E-21	Glioma
3225	1.858782439	2.91E-13	Glioma
9493	1.862039084	1.54E-17	Glioma
1296	1.862195043	5.03E-25	Glioma
136288	1.866954615	6.00E-09	Glioma
137835	1.869410097	1.72E-18	Glioma
914	1.871078873	2.93E-14	Glioma
972	1.873084506	2.25E-26	Glioma
160364	1.873787315	3.61E-17	Glioma
146722	1.874516469	8.33E-29	Glioma
400043	1.875236292	1.38E-26	Glioma
348378	1.877158786	1.06E-28	Glioma
871	1.878171516	1.31E-30	Glioma
63898	1.879485687	3.75E-14	Glioma
221476	1.880539209	7.26E-12	Glioma
54360	1.881083668	1.23E-24	Glioma
84106	1.88340707	4.16E-32	Glioma
1588	1.884820905	1.49E-20	Glioma
2297	1.885694671	3.59E-23	Glioma
26191	1.8881242	2.94E-19	Glioma
493869	1.88874864	1.01E-13	Glioma
84229	1.888894149	8.34E-15	Glioma
5742	1.89273948	2.54E-31	Glioma
284021	1.893126094	1.59E-30	Glioma
55247	1.893913865	1.96E-14	Glioma

3955	1.899363283	5.36E-39	Glioma
5355	1.900771625	4.79E-23	Glioma
2151	1.900821959	2.30E-14	Glioma
3689	1.901155532	2.01E-38	Glioma
3311	1.901509877	3.59E-17	Glioma
400759	1.901643085	3.99E-16	Glioma
3381	1.902246452	1.29E-07	Glioma
286530	1.903395104	6.12E-23	Glioma
717	1.912287275	4.97E-29	Glioma
55143	1.912593074	1.55E-23	Glioma
1288	1.91389447	1.65E-16	Glioma
8324	1.915571226	3.22E-25	Glioma
2150	1.918346103	2.13E-22	Glioma
822	1.921511367	8.70E-30	Glioma
55584	1.924996801	2.75E-10	Glioma
145258	1.925094237	6.74E-19	Glioma
9518	1.926139008	3.13E-11	Glioma
834	1.926630381	1.31E-35	Glioma
579	1.92665692	4.21E-17	Glioma
346389	1.927955364	8.19E-19	Glioma
7097	1.933927843	2.48E-31	Glioma
1284	1.934895402	6.37E-22	Glioma
79961	1.935337165	4.03E-31	Glioma
6121	1.938168076	3.64E-16	Glioma
115265	1.94399013	2.06E-08	Glioma
367	1.945871279	2.06E-22	Glioma
55013	1.94666355	1.27E-22	Glioma
283431	1.948631284	1.37E-19	Glioma
3118	1.951332384	1.16E-11	Glioma
8942	1.952142598	2.79E-22	Glioma
151648	1.953404872	5.34E-16	Glioma
2318	1.957339267	3.48E-18	Glioma
113730	1.957580359	2.20E-25	Glioma
3122	1.959453605	1.88E-21	Glioma
3111	1.961395707	9.44E-23	Glioma
7050	1.96385433	1.25E-41	Glioma
715	1.964701575	1.13E-25	Glioma
11025	1.968219699	2.12E-27	Glioma
6373	1.968664061	4.57E-12	Glioma
27036	1.968788914	2.17E-27	Glioma
26499	1.977274468	4.24E-16	Glioma
56245	1.980847481	2.10E-16	Glioma
9103	1.981525102	1.17E-17	Glioma
55355	1.983365438	3.93E-17	Glioma
5996	1.984808915	8.47E-20	Glioma
83596	1.987673026	1.16E-39	Glioma
4973	1.991904437	1.02E-22	Glioma
55635	1.994904224	5.97E-17	Glioma
5104	1.99536371	3.21E-10	Glioma
8190	1.996614612	1.33E-17	Glioma
64151	1.997861993	4.39E-16	Glioma
654817	1.998037129	2.95E-23	Glioma
9902	2.001363475	9.61E-36	Glioma
54209	2.001499771	2.39E-31	Glioma
81030	2.006681196	4.30E-20	Glioma
2203	2.010335186	3.79E-35	Glioma
55843	2.020327727	9.10E-28	Glioma
9700	2.023077173	8.06E-18	Glioma

55723	2.023453477	7.90E-22	Glioma
100233209	2.023741108	2.53E-23	Glioma
995	2.025675422	1.64E-18	Glioma
3117	2.028516257	3.02E-15	Glioma
10964	2.031359604	6.41E-25	Glioma
4998	2.031784969	2.63E-26	Glioma
388512	2.033124878	6.31E-16	Glioma
8038	2.034513361	1.31E-17	Glioma
2012	2.038486631	4.48E-27	Glioma
10788	2.040879747	1.54E-22	Glioma
8475	2.041831999	3.86E-16	Glioma
157313	2.045662059	4.45E-20	Glioma
81930	2.048899388	1.92E-21	Glioma
653361	2.049253636	2.30E-29	Glioma
7076	2.053975186	5.86E-15	Glioma
163702	2.056139892	1.21E-33	Glioma
9388	2.057226899	6.32E-26	Glioma
388325	2.058728424	3.70E-35	Glioma
960	2.061403063	5.47E-25	Glioma
6282	2.067285066	6.83E-34	Glioma
3223	2.067844223	8.07E-14	Glioma
2357	2.068288516	1.89E-24	Glioma
861	2.071635371	3.41E-33	Glioma
3683	2.072519394	2.80E-36	Glioma
80835	2.078669583	1.09E-25	Glioma
8061	2.080237174	5.67E-20	Glioma
4837	2.080527602	2.31E-12	Glioma
2302	2.082563978	4.02E-22	Glioma
3976	2.082670449	1.47E-15	Glioma
3120	2.093826679	2.72E-15	Glioma
3037	2.095604864	2.69E-22	Glioma
259266	2.095705627	2.27E-18	Glioma
332	2.096213046	5.28E-16	Glioma
10538	2.10079515	1.17E-25	Glioma
5077	2.101858888	4.37E-14	Glioma
3691	2.101905603	1.57E-24	Glioma
132884	2.104338314	5.82E-17	Glioma
3371	2.104672211	3.42E-26	Glioma
1134	2.106293298	3.29E-12	Glioma
54478	2.106928963	8.82E-17	Glioma
2214	2.107389561	5.38E-28	Glioma
55165	2.115555394	2.84E-20	Glioma
304	2.116725183	8.88E-22	Glioma
3236	2.11831593	2.70E-11	Glioma
7153	2.11842515	7.49E-14	Glioma
64231	2.119137649	9.62E-25	Glioma
6275	2.120866797	2.74E-18	Glioma
114614	2.121817475	1.95E-25	Glioma
2634	2.125821312	1.08E-28	Glioma
3199	2.126996651	1.06E-12	Glioma
3237	2.130460954	4.70E-13	Glioma
344	2.133557589	6.27E-28	Glioma
962	2.136553385	1.71E-19	Glioma
718	2.137045702	1.23E-28	Glioma
53831	2.138794178	6.76E-26	Glioma
3239	2.144541935	5.87E-12	Glioma
4283	2.144628539	2.48E-14	Glioma
597	2.151004873	3.66E-22	Glioma

241	2.152224708	6.16E-28	Glioma
283120	2.152641174	3.19E-08	Glioma
8318	2.155852302	3.17E-15	Glioma
6241	2.160964085	1.47E-15	Glioma
202309	2.162105202	4.26E-23	Glioma
165	2.170314908	6.41E-20	Glioma
5054	2.173844533	1.64E-17	Glioma
23529	2.174589968	2.86E-25	Glioma
27180	2.178826349	6.33E-37	Glioma
10644	2.179837251	2.86E-15	Glioma
6768	2.180142719	1.65E-29	Glioma
647946	2.181355336	1.49E-15	Glioma
79733	2.18806182	1.09E-23	Glioma
5806	2.197796252	3.03E-20	Glioma
1356	2.205159071	2.24E-14	Glioma
90050	2.208634223	3.87E-16	Glioma
1234	2.209592937	8.54E-29	Glioma
340267	2.217798485	5.81E-18	Glioma
136332	2.223926832	6.55E-27	Glioma
3242	2.224194918	1.63E-13	Glioma
1282	2.227830256	1.15E-21	Glioma
114769	2.230338469	2.25E-28	Glioma
1870	2.233965707	2.34E-23	Glioma
2735	2.234236965	1.89E-22	Glioma
57817	2.236514202	8.85E-16	Glioma
9133	2.241917157	8.13E-18	Glioma
5320	2.246698238	1.20E-09	Glioma
55240	2.251844206	1.84E-25	Glioma
115273	2.252127349	4.67E-31	Glioma
64843	2.254249426	2.41E-20	Glioma
10403	2.257364611	1.30E-19	Glioma
79054	2.26000713	1.26E-12	Glioma
4323	2.261767209	8.76E-43	Glioma
57664	2.2686239	1.06E-31	Glioma
2359	2.270335998	2.42E-21	Glioma
1281	2.271310627	4.35E-15	Glioma
991	2.277157923	2.65E-24	Glioma
9833	2.285379537	7.62E-17	Glioma
4481	2.285884212	1.69E-31	Glioma
969	2.300517938	5.43E-26	Glioma
4261	2.301045059	1.05E-30	Glioma
9332	2.311226961	6.91E-16	Glioma
2633	2.311887902	1.01E-30	Glioma
169044	2.31466441	5.13E-13	Glioma
11065	2.317545762	9.07E-18	Glioma
301	2.320828922	2.77E-19	Glioma
3207	2.320832552	4.62E-16	Glioma
79730	2.321007324	2.34E-23	Glioma
11004	2.324453475	1.52E-27	Glioma
3235	2.32509021	9.23E-13	Glioma
6424	2.325210511	1.69E-24	Glioma
5265	2.326114439	2.05E-31	Glioma
196410	2.330415365	2.23E-16	Glioma
4237	2.335417983	2.27E-18	Glioma
6696	2.341857849	1.84E-27	Glioma
2014	2.342567095	6.16E-16	Glioma
4608	2.345296701	4.18E-19	Glioma
10024	2.3500485	3.00E-20	Glioma

283971	2.351223808	8.92E-25	Glioma
9464	2.369533018	4.81E-16	Glioma
1475	2.371007724	2.58E-20	Glioma
9620	2.377333087	1.30E-24	Glioma
115362	2.378944218	6.11E-23	Glioma
5328	2.37956028	5.79E-28	Glioma
10112	2.411511048	2.04E-18	Glioma
5307	2.417971987	5.06E-14	Glioma
1058	2.426229795	1.03E-22	Glioma
9212	2.426362283	3.18E-19	Glioma
54210	2.428853998	4.44E-16	Glioma
4318	2.437365846	3.59E-11	Glioma
7431	2.452646331	1.73E-37	Glioma
6036	2.465805181	1.49E-24	Glioma
10437	2.474167132	3.60E-37	Glioma
85477	2.477791093	2.10E-28	Glioma
79258	2.479362477	7.53E-20	Glioma
10568	2.486273525	1.23E-20	Glioma
80380	2.487216156	9.61E-30	Glioma
27286	2.489807943	6.16E-20	Glioma
27197	2.502439898	1.19E-22	Glioma
3485	2.503392295	3.12E-19	Glioma
2015	2.506151348	6.76E-22	Glioma
1277	2.507263217	6.77E-18	Glioma
2020	2.524928657	2.24E-27	Glioma
352909	2.529568141	8.11E-27	Glioma
116039	2.530046543	3.22E-20	Glioma
8477	2.531735354	2.28E-33	Glioma
2019	2.532827148	1.26E-12	Glioma
27022	2.560523651	1.05E-24	Glioma
3200	2.599882427	1.33E-14	Glioma
118932	2.609152219	1.17E-26	Glioma
497190	2.616463541	1.70E-31	Glioma
10631	2.629277416	4.42E-09	Glioma
1890	2.633061175	2.28E-38	Glioma
6556	2.636929031	3.33E-39	Glioma
10643	2.648334099	5.54E-24	Glioma
51330	2.656608117	1.78E-29	Glioma
5778	2.664417776	2.57E-36	Glioma
407977	2.664742295	3.48E-19	Glioma
3214	2.725492891	7.44E-18	Glioma
1482	2.729510787	1.92E-17	Glioma
3212	2.736277548	2.46E-20	Glioma
1800	2.738045802	3.28E-16	Glioma
7412	2.740519964	3.51E-33	Glioma
90187	2.75165844	9.18E-16	Glioma
146802	2.752289995	1.27E-19	Glioma
9787	2.752385352	3.93E-21	Glioma
3226	2.770221282	1.12E-14	Glioma
4605	2.790292102	1.68E-20	Glioma
3198	2.791878006	1.62E-27	Glioma
3202	2.82712452	2.00E-17	Glioma
3221	2.835828491	6.27E-27	Glioma
3201	2.839339278	1.61E-15	Glioma
171558	2.842211803	1.64E-47	Glioma
3217	2.845172686	2.79E-26	Glioma
12	2.856969823	2.31E-22	Glioma
90853	2.858603486	2.50E-20	Glioma

10265	2.879786231	1.19E-24	Glioma
51316	2.898221091	1.43E-29	Glioma
1116	2.916222552	2.25E-11	Glioma
6274	2.953643777	3.78E-28	Glioma
761	3.005046176	3.81E-23	Glioma
23601	3.01935829	2.98E-19	Glioma
8857	3.054090748	5.63E-34	Glioma
7044	3.06138992	4.77E-24	Glioma
80832	3.070693248	8.51E-34	Glioma
23440	3.13114835	2.44E-20	Glioma
1117	3.141881509	4.77E-21	Glioma
256236	3.183006312	2.58E-31	Glioma
8714	3.211291865	7.11E-20	Glioma
3627	3.29376085	7.74E-22	Glioma
63950	3.293842687	1.90E-20	Glioma
4223	3.308167633	1.76E-15	Glioma
10630	3.461233613	4.85E-31	Glioma
3213	3.508454742	6.23E-22	Glioma
4057	3.565386322	1.09E-14	Glioma
3206	3.622270869	7.15E-21	Glioma
3204	3.897983815	7.10E-21	Glioma
6474	4.133045599	4.23E-26	Glioma
81557	3.496120228	2.51E-27	Clear cell renal cell
1311	3.403953707	5.90E-19	Clear cell renal cell
728239	3.359186273	4.68E-25	Clear cell renal cell
4237	3.34794056	1.37E-34	Clear cell renal cell
164284	3.311828664	1.05E-25	Clear cell renal cell
1301	3.272224018	4.22E-18	Clear cell renal cell
1294	3.250896578	5.62E-25	Clear cell renal cell
127435	3.247847689	7.12E-37	Clear cell renal cell
4239	3.245908597	2.72E-26	Clear cell renal cell
6366	3.235510412	1.40E-14	Clear cell renal cell
3671	3.221883211	9.87E-32	Clear cell renal cell
56265	3.14128362	1.79E-30	Clear cell renal cell
4060	3.12172967	4.06E-22	Clear cell renal cell
1634	3.087111523	2.19E-21	Clear cell renal cell
144347	3.080528788	1.38E-22	Clear cell renal cell
1674	3.075753842	1.55E-14	Clear cell renal cell
1277	2.994748108	2.04E-41	Clear cell renal cell
26585	2.975256084	1.78E-18	Clear cell renal cell
6423	2.933822434	3.41E-13	Clear cell renal cell
6363	2.878601753	1.91E-16	Clear cell renal cell
8532	2.836015637	5.10E-17	Clear cell renal cell
5740	2.826850436	4.96E-30	Clear cell renal cell
79883	2.810356804	2.21E-24	Clear cell renal cell
51050	2.773707108	1.43E-20	Clear cell renal cell
11117	2.75593166	7.63E-46	Clear cell renal cell
2191	2.744008295	2.21E-37	Clear cell renal cell
3481	2.738760416	4.87E-22	Clear cell renal cell
1289	2.732498051	4.00E-42	Clear cell renal cell
29774	2.703899238	4.07E-35	Clear cell renal cell
5156	2.702188062	3.82E-21	Clear cell renal cell
25789	2.700454682	2.59E-28	Clear cell renal cell
4969	2.674758623	2.80E-13	Clear cell renal cell
5549	2.661461407	1.77E-26	Clear cell renal cell
51450	2.659744646	1.71E-32	Clear cell renal cell
2735	2.659637895	2.67E-38	Clear cell renal cell
7058	2.636318697	1.61E-26	Clear cell renal cell

1305	2.626079743	4.02E-35	Clear cell renal cell
8745	2.620558491	1.64E-24	Clear cell renal cell
730	2.60528339	1.68E-16	Clear cell renal cell
150	2.60435727	1.77E-30	Clear cell renal cell
2307	2.600452199	1.64E-45	Clear cell renal cell
284297	2.597970843	2.33E-34	Clear cell renal cell
65012	2.597802787	3.15E-31	Clear cell renal cell
84624	2.585766583	1.08E-24	Clear cell renal cell
126393	2.56920921	5.64E-30	Clear cell renal cell
2949	2.564180636	4.59E-27	Clear cell renal cell
63876	2.554461661	4.87E-38	Clear cell renal cell
8839	2.550212979	2.53E-13	Clear cell renal cell
1300	2.543471155	2.14E-16	Clear cell renal cell
22801	2.540577312	4.39E-36	Clear cell renal cell
3880	2.531096078	2.53E-10	Clear cell renal cell
3569	2.508745776	6.49E-15	Clear cell renal cell
4188	2.50733373	3.84E-44	Clear cell renal cell
8038	2.504669607	2.77E-22	Clear cell renal cell
1307	2.50427313	2.07E-32	Clear cell renal cell
9096	2.503275572	4.06E-26	Clear cell renal cell
148741	2.491605026	2.73E-40	Clear cell renal cell
338773	2.491477447	1.65E-32	Clear cell renal cell
10536	2.490399584	1.11E-31	Clear cell renal cell
8510	2.487404876	6.81E-33	Clear cell renal cell
10290	2.48096998	1.24E-26	Clear cell renal cell
11341	2.468620575	3.55E-18	Clear cell renal cell
338707	2.45060514	4.68E-12	Clear cell renal cell
27286	2.445235054	8.28E-22	Clear cell renal cell
92745	2.442900333	8.00E-15	Clear cell renal cell
23627	2.435563385	1.18E-16	Clear cell renal cell
389336	2.434129656	4.83E-09	Clear cell renal cell
389558	2.428456546	4.60E-23	Clear cell renal cell
4062	2.428167312	1.70E-20	Clear cell renal cell
116535	2.41009165	2.26E-25	Clear cell renal cell
64220	2.409468132	6.27E-19	Clear cell renal cell
55384	2.401282179	7.62E-21	Clear cell renal cell
81493	2.398715449	1.17E-20	Clear cell renal cell
2784	2.393044317	1.98E-25	Clear cell renal cell
1278	2.391062909	5.90E-39	Clear cell renal cell
7781	2.383397417	1.75E-27	Clear cell renal cell
5317	2.382555646	4.31E-13	Clear cell renal cell
10344	2.381501801	2.14E-24	Clear cell renal cell
57419	2.374010665	1.87E-24	Clear cell renal cell
1293	2.373802729	6.25E-34	Clear cell renal cell
2736	2.371602906	1.03E-40	Clear cell renal cell
90993	2.369294005	3.31E-18	Clear cell renal cell
6664	2.353842062	7.79E-19	Clear cell renal cell
5099	2.342050267	9.89E-25	Clear cell renal cell
728264	2.338072297	1.02E-26	Clear cell renal cell
9244	2.323822608	7.24E-16	Clear cell renal cell
8076	2.321065112	2.97E-13	Clear cell renal cell
131578	2.314433213	1.89E-12	Clear cell renal cell
1734	2.310321743	1.24E-20	Clear cell renal cell
1281	2.309690247	1.44E-37	Clear cell renal cell
7373	2.30950641	6.36E-26	Clear cell renal cell
80332	2.304248336	5.42E-16	Clear cell renal cell
1805	2.302943809	2.19E-14	Clear cell renal cell
222865	2.299455493	1.60E-10	Clear cell renal cell

56917	2.290795365	7.29E-40	Clear cell renal cell
1382	2.281054014	3.83E-14	Clear cell renal cell
89795	2.255815884	4.78E-25	Clear cell renal cell
6999	2.255306167	3.07E-14	Clear cell renal cell
7140	2.251753474	4.34E-15	Clear cell renal cell
2583	2.249494621	4.19E-20	Clear cell renal cell
1264	2.247804235	1.04E-25	Clear cell renal cell
28954	2.247729506	5.51E-36	Clear cell renal cell
7291	2.246334798	2.12E-31	Clear cell renal cell
11211	2.245833127	1.24E-21	Clear cell renal cell
8643	2.245676934	3.44E-36	Clear cell renal cell
55220	2.244593623	1.65E-25	Clear cell renal cell
8534	2.236206062	1.83E-31	Clear cell renal cell
2006	2.231027402	5.19E-24	Clear cell renal cell
259232	2.227417408	1.86E-14	Clear cell renal cell
3037	2.225805207	1.08E-22	Clear cell renal cell
4920	2.225493328	1.02E-13	Clear cell renal cell
11075	2.222760966	4.82E-13	Clear cell renal cell
90853	2.217791762	1.52E-22	Clear cell renal cell
114898	2.211963703	6.12E-34	Clear cell renal cell
2019	2.204982773	1.89E-20	Clear cell renal cell
1745	2.20368527	1.50E-27	Clear cell renal cell
5118	2.19810574	1.75E-42	Clear cell renal cell
2687	2.198067056	2.31E-40	Clear cell renal cell
25884	2.194619804	4.67E-12	Clear cell renal cell
3872	2.191459125	1.14E-12	Clear cell renal cell
4016	2.191426846	1.11E-25	Clear cell renal cell
8091	2.19082386	1.11E-12	Clear cell renal cell
1292	2.19081089	6.54E-40	Clear cell renal cell
57611	2.190392927	1.55E-26	Clear cell renal cell
5396	2.187761035	1.21E-24	Clear cell renal cell
148398	2.186006802	4.94E-15	Clear cell renal cell
4916	2.179984967	4.70E-23	Clear cell renal cell
57722	2.173727431	2.16E-12	Clear cell renal cell
9358	2.167856976	2.38E-19	Clear cell renal cell
125	2.158789059	5.39E-10	Clear cell renal cell
5348	2.153970786	7.40E-12	Clear cell renal cell
140766	2.147756665	3.98E-19	Clear cell renal cell
4803	2.145518946	1.76E-25	Clear cell renal cell
4318	2.143422943	2.70E-13	Clear cell renal cell
1001	2.142513372	1.44E-17	Clear cell renal cell
7169	2.135794381	1.17E-41	Clear cell renal cell
57595	2.134954759	6.48E-35	Clear cell renal cell
85449	2.133522817	6.87E-25	Clear cell renal cell
6876	2.127170278	1.41E-34	Clear cell renal cell
389136	2.126915001	1.01E-20	Clear cell renal cell
10267	2.12506367	4.74E-15	Clear cell renal cell
10631	2.124484324	5.06E-21	Clear cell renal cell
54829	2.123751241	8.90E-28	Clear cell renal cell
5739	2.122297158	2.06E-40	Clear cell renal cell
64094	2.121033231	7.73E-24	Clear cell renal cell
7051	2.119584613	5.27E-16	Clear cell renal cell
389125	2.118194088	1.54E-23	Clear cell renal cell
6356	2.117065652	4.55E-16	Clear cell renal cell
147906	2.114747384	2.72E-38	Clear cell renal cell
7018	2.113501116	1.30E-07	Clear cell renal cell
1749	2.10681088	5.72E-28	Clear cell renal cell
165	2.106049373	1.58E-20	Clear cell renal cell

6615	2.101285098	8.77E-39	Clear cell renal cell
3787	2.098453935	4.78E-08	Clear cell renal cell
90249	2.09690563	3.89E-19	Clear cell renal cell
91977	2.096768082	4.94E-30	Clear cell renal cell
65997	2.092096876	1.90E-15	Clear cell renal cell
94274	2.088163814	8.16E-29	Clear cell renal cell
8908	2.087918545	3.52E-10	Clear cell renal cell
3237	2.087915254	2.57E-24	Clear cell renal cell
8425	2.08641441	5.30E-32	Clear cell renal cell
24141	2.082913417	2.89E-13	Clear cell renal cell
222663	2.077342657	1.87E-23	Clear cell renal cell
27190	2.07639055	1.07E-27	Clear cell renal cell
5143	2.075375551	4.56E-20	Clear cell renal cell
54751	2.069394383	5.32E-46	Clear cell renal cell
633	2.069070719	8.28E-33	Clear cell renal cell
10149	2.066143794	3.69E-13	Clear cell renal cell
79148	2.060511157	1.27E-32	Clear cell renal cell
140689	2.059150825	3.03E-14	Clear cell renal cell
57689	2.0585075	6.19E-15	Clear cell renal cell
10391	2.053656969	7.32E-26	Clear cell renal cell
7857	2.0493441	4.07E-15	Clear cell renal cell
2662	2.04890851	1.04E-18	Clear cell renal cell
3357	2.045485941	2.47E-21	Clear cell renal cell
5999	2.045430618	1.88E-30	Clear cell renal cell
58189	2.035680243	4.08E-27	Clear cell renal cell
9241	2.031523108	3.26E-10	Clear cell renal cell
9902	2.030002	1.97E-35	Clear cell renal cell
9022	2.027257815	6.63E-22	Clear cell renal cell
4629	2.024406291	3.53E-17	Clear cell renal cell
6447	2.021939511	1.77E-12	Clear cell renal cell
340075	2.021578861	7.88E-12	Clear cell renal cell
148113	2.021277627	7.52E-13	Clear cell renal cell
2568	2.012725968	3.16E-12	Clear cell renal cell
3624	2.0058448	5.36E-25	Clear cell renal cell
5322	1.999690414	1.10E-18	Clear cell renal cell
2056	1.995626828	3.12E-06	Clear cell renal cell
2192	1.991658295	1.38E-23	Clear cell renal cell
51200	1.990927505	6.57E-08	Clear cell renal cell
89822	1.988490732	8.54E-13	Clear cell renal cell
7732	1.988105336	9.59E-28	Clear cell renal cell
4776	1.987463574	4.75E-41	Clear cell renal cell
23284	1.987007138	2.93E-20	Clear cell renal cell
70	1.986430318	2.10E-09	Clear cell renal cell
57124	1.983600039	1.29E-32	Clear cell renal cell
26002	1.977201055	1.38E-15	Clear cell renal cell
55190	1.975873057	5.53E-23	Clear cell renal cell
83468	1.97425593	3.35E-34	Clear cell renal cell
4958	1.973801762	1.08E-13	Clear cell renal cell
1272	1.973581117	1.47E-10	Clear cell renal cell
3290	1.968330126	1.33E-14	Clear cell renal cell
4053	1.968257118	9.69E-29	Clear cell renal cell
374654	1.964114166	6.35E-38	Clear cell renal cell
151887	1.963060612	5.64E-23	Clear cell renal cell
4313	1.961011706	1.32E-32	Clear cell renal cell
50863	1.956701213	3.10E-13	Clear cell renal cell
219348	1.95581447	1.59E-18	Clear cell renal cell
126129	1.955282753	2.10E-36	Clear cell renal cell
57333	1.953293733	7.42E-35	Clear cell renal cell

731220	1.95103717	5.46E-19	Clear cell renal cell
84446	1.949142215	3.74E-25	Clear cell renal cell
3485	1.945661382	1.40E-22	Clear cell renal cell
2318	1.94257597	5.26E-13	Clear cell renal cell
23114	1.94143333	1.07E-26	Clear cell renal cell
353500	1.938749377	7.24E-31	Clear cell renal cell
2331	1.937512711	1.16E-14	Clear cell renal cell
91179	1.937438648	7.78E-32	Clear cell renal cell
1893	1.935731114	1.33E-35	Clear cell renal cell
25907	1.934886802	4.57E-22	Clear cell renal cell
54039	1.932050411	3.14E-09	Clear cell renal cell
50509	1.931208555	7.04E-27	Clear cell renal cell
2173	1.928840581	0.000474467	Clear cell renal cell
283208	1.927514061	3.82E-20	Clear cell renal cell
79987	1.926421432	5.90E-21	Clear cell renal cell
5021	1.925897525	3.18E-22	Clear cell renal cell
196500	1.922588623	1.28E-18	Clear cell renal cell
23768	1.922086003	1.32E-24	Clear cell renal cell
81792	1.921869231	5.38E-17	Clear cell renal cell
25903	1.92095006	5.30E-36	Clear cell renal cell
10278	1.920510681	2.17E-23	Clear cell renal cell
163782	1.918922339	6.16E-21	Clear cell renal cell
6590	1.917732371	4.35E-05	Clear cell renal cell
388125	1.915517456	1.99E-16	Clear cell renal cell
4854	1.911552508	9.83E-34	Clear cell renal cell
3090	1.911524065	5.93E-40	Clear cell renal cell
79776	1.910697525	2.00E-16	Clear cell renal cell
145820	1.907045576	4.36E-22	Clear cell renal cell
65989	1.903681505	1.75E-13	Clear cell renal cell
51332	1.90264807	5.04E-17	Clear cell renal cell
91851	1.899975437	7.91E-06	Clear cell renal cell
10319	1.898049132	3.12E-14	Clear cell renal cell
10882	1.896131853	1.43E-07	Clear cell renal cell
3381	1.894549463	3.09E-09	Clear cell renal cell
145864	1.893048128	6.87E-33	Clear cell renal cell
401115	1.890730313	9.22E-15	Clear cell renal cell
2295	1.890537846	1.26E-23	Clear cell renal cell
8912	1.882269969	1.03E-28	Clear cell renal cell
7045	1.879372911	5.19E-11	Clear cell renal cell
57194	1.879237359	1.63E-26	Clear cell renal cell
81285	1.878775023	2.71E-21	Clear cell renal cell
2912	1.876779462	2.70E-27	Clear cell renal cell
85366	1.875633209	1.64E-24	Clear cell renal cell
9890	1.875138404	1.50E-18	Clear cell renal cell
7477	1.871291979	8.17E-11	Clear cell renal cell
6320	1.870354575	6.29E-37	Clear cell renal cell
84628	1.868486943	8.24E-21	Clear cell renal cell
9945	1.868381641	2.00E-11	Clear cell renal cell
84894	1.867681402	2.60E-28	Clear cell renal cell
57125	1.867618908	3.46E-34	Clear cell renal cell
10516	1.866665994	1.98E-21	Clear cell renal cell
1303	1.866187102	3.91E-31	Clear cell renal cell
23213	1.865576196	6.54E-28	Clear cell renal cell
51339	1.864874975	6.71E-27	Clear cell renal cell
2596	1.864341667	2.16E-18	Clear cell renal cell
8786	1.862481893	7.11E-17	Clear cell renal cell
3371	1.859549013	1.72E-18	Clear cell renal cell
284069	1.856496455	1.11E-24	Clear cell renal cell

6553	1.854845368	8.72E-30	Clear cell renal cell
5159	1.85429682	9.81E-35	Clear cell renal cell
135398	1.853886817	2.47E-13	Clear cell renal cell
3908	1.852724668	1.88E-15	Clear cell renal cell
388585	1.851314487	3.95E-17	Clear cell renal cell
1290	1.851191428	1.94E-30	Clear cell renal cell
4326	1.849329912	8.03E-13	Clear cell renal cell
6288	1.848202776	0.000833077	Clear cell renal cell
92304	1.847744142	1.34E-19	Clear cell renal cell
5837	1.84645645	3.38E-23	Clear cell renal cell
100130958	1.845738532	2.85E-14	Clear cell renal cell
115557	1.844091338	4.34E-40	Clear cell renal cell
72	1.843209016	8.22E-15	Clear cell renal cell
114905	1.843191962	4.16E-19	Clear cell renal cell
85016	1.843132757	5.19E-13	Clear cell renal cell
387763	1.840658303	1.29E-26	Clear cell renal cell
6444	1.838826426	6.66E-13	Clear cell renal cell
57214	1.838025343	5.51E-13	Clear cell renal cell
5228	1.835867205	5.82E-13	Clear cell renal cell
57863	1.835573598	4.30E-08	Clear cell renal cell
176	1.834001148	9.26E-13	Clear cell renal cell
56963	1.832432265	4.34E-28	Clear cell renal cell
7148	1.829863792	5.79E-22	Clear cell renal cell
5365	1.827674043	1.69E-14	Clear cell renal cell
8728	1.82660633	2.55E-34	Clear cell renal cell
91683	1.82632724	9.25E-12	Clear cell renal cell
54361	1.82386877	7.83E-16	Clear cell renal cell
117581	1.821710374	1.54E-23	Clear cell renal cell
55698	1.821281555	4.96E-34	Clear cell renal cell
815	1.815802234	2.24E-20	Clear cell renal cell
25878	1.815510628	5.02E-19	Clear cell renal cell
114897	1.813922685	2.77E-22	Clear cell renal cell
775	1.813784428	8.15E-27	Clear cell renal cell
54328	1.811207577	1.64E-17	Clear cell renal cell
145258	1.81041422	1.05E-24	Clear cell renal cell
29798	1.810226776	6.34E-23	Clear cell renal cell
169044	1.809896934	5.77E-08	Clear cell renal cell
83643	1.808621892	1.89E-26	Clear cell renal cell
93649	1.808170279	3.25E-13	Clear cell renal cell
143503	1.804678483	1.57E-24	Clear cell renal cell
85352	1.80281929	1.62E-10	Clear cell renal cell
59	1.80063879	3.07E-30	Clear cell renal cell
81794	1.800353911	9.86E-20	Clear cell renal cell
7041	1.800341454	4.10E-41	Clear cell renal cell
2843	1.799455745	3.82E-18	Clear cell renal cell
26508	1.796399744	8.14E-29	Clear cell renal cell
4325	1.79469412	9.23E-17	Clear cell renal cell
7138	1.791948743	1.49E-06	Clear cell renal cell
2563	1.79156184	1.47E-15	Clear cell renal cell
24	1.790505857	1.72E-20	Clear cell renal cell
6357	1.790101489	7.01E-13	Clear cell renal cell
53637	1.789329847	6.97E-30	Clear cell renal cell
57348	1.786610064	4.80E-15	Clear cell renal cell
80863	1.784231472	1.70E-36	Clear cell renal cell
2042	1.783639902	3.59E-18	Clear cell renal cell
8817	1.782621146	1.25E-20	Clear cell renal cell
199800	1.782532657	1.27E-27	Clear cell renal cell
2152	1.781585039	2.55E-16	Clear cell renal cell

55107	1.780652436	2.10E-27	Clear cell renal cell
4355	1.77989377	1.85E-16	Clear cell renal cell
145270	1.779478766	4.49E-05	Clear cell renal cell
80307	1.778990288	7.32E-10	Clear cell renal cell
10468	1.778781769	1.78E-09	Clear cell renal cell
27123	1.778400436	9.87E-13	Clear cell renal cell
8406	1.777826199	7.04E-21	Clear cell renal cell
84251	1.777303842	2.11E-18	Clear cell renal cell
3750	1.775812388	1.08E-34	Clear cell renal cell
55806	1.771417348	6.76E-14	Clear cell renal cell
4256	1.768916733	1.21E-29	Clear cell renal cell
84940	1.767658404	2.87E-11	Clear cell renal cell
2826	1.762091969	5.13E-25	Clear cell renal cell
8877	1.760533771	2.13E-24	Clear cell renal cell
25960	1.758762989	3.05E-35	Clear cell renal cell
80128	1.758391533	2.92E-17	Clear cell renal cell
163071	1.756462034	3.94E-09	Clear cell renal cell
7076	1.755469972	4.66E-29	Clear cell renal cell
3706	1.753942026	7.50E-10	Clear cell renal cell
4747	1.753318276	2.75E-06	Clear cell renal cell
6442	1.752419666	1.26E-15	Clear cell renal cell
26108	1.751822443	2.34E-17	Clear cell renal cell
7223	1.750890184	7.38E-23	Clear cell renal cell
6586	1.749953441	9.87E-23	Clear cell renal cell
728392	1.746276684	2.74E-33	Clear cell renal cell
57596	1.744862257	1.18E-21	Clear cell renal cell
768	1.743296889	1.86E-05	Clear cell renal cell
162461	1.741686283	7.86E-07	Clear cell renal cell
25802	1.741444097	3.67E-22	Clear cell renal cell
94031	1.741050934	7.80E-24	Clear cell renal cell
11174	1.738560581	1.32E-27	Clear cell renal cell
8755	1.737456539	3.52E-08	Clear cell renal cell
2906	1.735843954	1.04E-24	Clear cell renal cell
8646	1.73359088	1.81E-17	Clear cell renal cell
9260	1.732809398	2.94E-48	Clear cell renal cell
1750	1.731315058	1.56E-22	Clear cell renal cell
25806	1.729086883	1.10E-17	Clear cell renal cell
54102	1.728277216	1.00E-06	Clear cell renal cell
51308	1.724838281	2.64E-12	Clear cell renal cell
4052	1.723450515	1.33E-23	Clear cell renal cell
150763	1.723084724	2.23E-18	Clear cell renal cell
57030	1.721027323	3.70E-20	Clear cell renal cell
51237	1.720293321	2.36E-08	Clear cell renal cell
283120	1.717465336	1.20E-11	Clear cell renal cell
10186	1.71698187	7.25E-35	Clear cell renal cell
5010	1.715344586	2.25E-18	Clear cell renal cell
79827	1.713528277	2.36E-07	Clear cell renal cell
2300	1.713225224	1.63E-18	Clear cell renal cell
4320	1.713127507	1.65E-31	Clear cell renal cell
2199	1.712494727	2.37E-19	Clear cell renal cell
54345	1.712131367	1.47E-25	Clear cell renal cell
4635	1.710018027	8.18E-23	Clear cell renal cell
51334	1.709991164	5.24E-26	Clear cell renal cell
115908	1.708973737	1.15E-12	Clear cell renal cell
1809	1.708209473	8.59E-27	Clear cell renal cell
339453	1.706895243	2.94E-23	Clear cell renal cell
54587	1.705719123	1.46E-13	Clear cell renal cell
8506	1.702117743	3.67E-29	Clear cell renal cell

5662	1.700331579	1.57E-21	Clear cell renal cell
9610	1.699956987	1.68E-24	Clear cell renal cell
84929	1.699232682	7.64E-07	Clear cell renal cell
124602	1.697437937	6.47E-20	Clear cell renal cell
163933	1.697344155	1.07E-15	Clear cell renal cell
9127	1.695556461	7.38E-18	Clear cell renal cell
5730	1.693281536	2.04E-09	Clear cell renal cell
2890	1.6901132	6.52E-10	Clear cell renal cell
5744	1.689403662	6.71E-06	Clear cell renal cell
2275	1.68923898	1.88E-46	Clear cell renal cell
643	1.687810414	4.76E-17	Clear cell renal cell
85407	1.687129008	5.60E-19	Clear cell renal cell
6943	1.686663596	3.47E-17	Clear cell renal cell
1949	1.685876926	6.79E-23	Clear cell renal cell
59352	1.685181405	4.22E-21	Clear cell renal cell
8784	1.680092465	2.21E-17	Clear cell renal cell
84958	1.678994553	4.17E-17	Clear cell renal cell
23416	1.678584329	6.23E-13	Clear cell renal cell
286333	1.678557298	1.67E-21	Clear cell renal cell
1464	1.678265476	8.64E-22	Clear cell renal cell
4316	1.676515928	3.15E-07	Clear cell renal cell
6939	1.676407777	4.60E-19	Clear cell renal cell
51673	1.676095075	4.99E-23	Clear cell renal cell
3866	1.675866493	7.38E-09	Clear cell renal cell
167681	1.674459183	1.19E-14	Clear cell renal cell
1036	1.672873578	6.80E-12	Clear cell renal cell
6853	1.672006438	2.36E-17	Clear cell renal cell
388610	1.671995793	3.52E-10	Clear cell renal cell
54551	1.671241068	8.53E-13	Clear cell renal cell
881	1.669124237	1.75E-21	Clear cell renal cell
11187	1.668571066	8.21E-06	Clear cell renal cell
57165	1.668497046	9.85E-18	Clear cell renal cell
10235	1.668063757	5.13E-28	Clear cell renal cell
11227	1.667452036	7.68E-07	Clear cell renal cell
168667	1.667159711	7.93E-16	Clear cell renal cell
57699	1.66590205	6.17E-24	Clear cell renal cell
773	1.665842394	8.99E-26	Clear cell renal cell
64838	1.665754937	2.78E-15	Clear cell renal cell
4440	1.663168864	3.62E-16	Clear cell renal cell
441478	1.662709363	1.57E-27	Clear cell renal cell
3955	1.662492444	8.09E-32	Clear cell renal cell
4017	1.661331375	5.86E-21	Clear cell renal cell
50615	1.660614269	1.80E-19	Clear cell renal cell
7293	1.6589677	7.79E-21	Clear cell renal cell
4900	1.658535828	1.85E-26	Clear cell renal cell
6262	1.655499344	5.24E-08	Clear cell renal cell
80119	1.65495428	1.47E-20	Clear cell renal cell
146206	1.654446555	2.52E-13	Clear cell renal cell
339483	1.652642708	1.21E-17	Clear cell renal cell
51285	1.652304585	3.76E-29	Clear cell renal cell
84206	1.6517018	6.44E-39	Clear cell renal cell
119	1.650583082	5.19E-11	Clear cell renal cell
116039	1.649220679	3.27E-14	Clear cell renal cell
139728	1.64891068	7.45E-05	Clear cell renal cell
25790	1.648858326	1.67E-23	Clear cell renal cell
4050	1.648123585	8.63E-17	Clear cell renal cell
53833	1.648076648	9.27E-06	Clear cell renal cell
283248	1.64434	3.08E-10	Clear cell renal cell

80864	1.644203136	4.95E-18	Clear cell renal cell
171024	1.643816796	2.34E-19	Clear cell renal cell
2627	1.642641689	2.29E-25	Clear cell renal cell
9645	1.642433786	1.05E-31	Clear cell renal cell
11178	1.641519237	1.93E-25	Clear cell renal cell
9834	1.641253009	4.17E-11	Clear cell renal cell
9796	1.64005926	4.64E-19	Clear cell renal cell
3783	1.639991622	2.32E-18	Clear cell renal cell
3142	1.639699367	4.14E-27	Clear cell renal cell
4908	1.638004317	1.23E-16	Clear cell renal cell
100124700	1.633723724	3.46E-20	Clear cell renal cell
5522	1.633410834	5.48E-07	Clear cell renal cell
23017	1.632361511	5.27E-15	Clear cell renal cell
56967	1.629884152	7.45E-13	Clear cell renal cell
7122	1.62848108	3.56E-20	Clear cell renal cell
54413	1.627793549	2.31E-29	Clear cell renal cell
6624	1.62730386	4.34E-37	Clear cell renal cell
169611	1.623471146	5.30E-20	Clear cell renal cell
2894	1.623319852	3.20E-13	Clear cell renal cell
5414	1.622642597	1.57E-32	Clear cell renal cell
9507	1.621255592	7.56E-18	Clear cell renal cell
27319	1.618662667	9.85E-18	Clear cell renal cell
4885	1.617252506	1.35E-06	Clear cell renal cell
7044	1.616488196	5.60E-11	Clear cell renal cell
3779	1.615624845	3.04E-25	Clear cell renal cell
29943	1.615404202	2.18E-06	Clear cell renal cell
6512	1.615167256	4.23E-09	Clear cell renal cell
9509	1.614597095	3.43E-19	Clear cell renal cell
4222	1.614232628	3.77E-15	Clear cell renal cell
8620	1.614163824	1.00E-17	Clear cell renal cell
26548	1.613235619	3.52E-21	Clear cell renal cell
100130776	1.613222291	3.30E-23	Clear cell renal cell
10052	1.612421868	7.40E-25	Clear cell renal cell
5979	1.61229045	2.00E-15	Clear cell renal cell
221687	1.611895094	5.63E-11	Clear cell renal cell
11173	1.611693067	2.95E-28	Clear cell renal cell
8061	1.611117395	1.54E-15	Clear cell renal cell
1396	1.609697245	1.56E-16	Clear cell renal cell
57172	1.609520051	8.09E-16	Clear cell renal cell
4889	1.60871072	5.94E-14	Clear cell renal cell
170685	1.60779069	1.51E-15	Clear cell renal cell
91608	1.607610639	1.68E-22	Clear cell renal cell
221476	1.605855749	2.08E-07	Clear cell renal cell
390205	1.605127366	3.05E-16	Clear cell renal cell
2328	1.604850968	1.05E-13	Clear cell renal cell
2786	1.604733035	5.70E-09	Clear cell renal cell
1909	1.60431264	2.63E-26	Clear cell renal cell
3976	1.604086103	2.38E-12	Clear cell renal cell
23025	1.60353953	4.02E-14	Clear cell renal cell
728215	1.601785822	7.32E-18	Clear cell renal cell
199786	1.601619544	5.16E-16	Clear cell renal cell
7262	1.600490931	1.88E-22	Clear cell renal cell
53354	-1.507614191	3.59E-22	Clear cell renal cell
5284	-1.507951471	0.000222788	Clear cell renal cell
27004	-1.513468219	1.22E-05	Clear cell renal cell
5313	-1.529387549	2.08E-05	Clear cell renal cell
202374	-1.531504081	1.80E-07	Clear cell renal cell
117153	-1.533968681	1.01E-09	Clear cell renal cell

1644	-1.541567691	0.00011935	Clear cell renal cell
7368	-1.553742339	3.73E-15	Clear cell renal cell
51458	-1.57402388	0.001147829	Clear cell renal cell
1579	-1.578284154	0.002021876	Clear cell renal cell
57393	-1.587616505	8.43E-08	Clear cell renal cell
1551	-1.596756257	2.72E-09	Clear cell renal cell
84803	-1.603324071	3.83E-15	Clear cell renal cell
345079	-1.610251954	1.68E-13	Clear cell renal cell
149466	-1.620430421	9.39E-09	Clear cell renal cell
5105	-1.630050839	3.55E-06	Clear cell renal cell
10891	-1.63307948	6.12E-12	Clear cell renal cell
115019	-1.637215985	4.71E-08	Clear cell renal cell
28999	-1.644131264	8.48E-17	Clear cell renal cell
4888	-1.645375854	1.16E-08	Clear cell renal cell
2638	-1.651051123	0.000149005	Clear cell renal cell
7113	-1.659242378	2.07E-05	Clear cell renal cell
116085	-1.661152697	0.000353606	Clear cell renal cell
29953	-1.689478884	1.85E-06	Clear cell renal cell
55753	-1.708056719	1.16E-10	Clear cell renal cell
151126	-1.715186878	4.11E-08	Clear cell renal cell
55089	-1.72091629	9.47E-07	Clear cell renal cell
9058	-1.721086991	1.96E-08	Clear cell renal cell
1950	-1.781691183	1.58E-09	Clear cell renal cell
134285	-1.814107814	1.93E-18	Clear cell renal cell
153328	-1.827394397	2.64E-10	Clear cell renal cell
51179	-1.828578733	3.18E-06	Clear cell renal cell
10840	-1.83442358	2.94E-10	Clear cell renal cell
9615	-1.83806396	6.14E-09	Clear cell renal cell
3158	-1.840233223	4.87E-05	Clear cell renal cell
80763	-1.872303893	3.83E-09	Clear cell renal cell
5618	-1.880621455	4.51E-17	Clear cell renal cell
9356	-1.881449221	0.000167137	Clear cell renal cell
5053	-1.927198795	1.43E-05	Clear cell renal cell
59272	-1.931554809	4.61E-06	Clear cell renal cell
387700	-1.940899955	5.94E-12	Clear cell renal cell
160728	-1.956313517	8.24E-05	Clear cell renal cell
2538	-2.017801622	4.25E-07	Clear cell renal cell
6523	-2.020814061	9.93E-07	Clear cell renal cell
2104	-2.054655395	1.01E-14	Clear cell renal cell
256764	-2.122602036	6.06E-19	Clear cell renal cell
643236	-2.126938622	1.72E-12	Clear cell renal cell
1586	-2.154980488	5.43E-10	Clear cell renal cell
10117	-2.300050938	1.82E-16	Clear cell renal cell
6561	-2.310601406	5.72E-07	Clear cell renal cell
134288	-2.340140444	4.13E-09	Clear cell renal cell
6561	-3.888291328	2.23E-09	Papillary renal cell
22977	-3.877815312	2.48E-16	Papillary renal cell
51179	-3.790340244	2.53E-10	Papillary renal cell
10864	-3.738890694	4.96E-10	Papillary renal cell
563	-3.693611765	1.34E-10	Papillary renal cell
5105	-3.654537749	1.86E-12	Papillary renal cell
9356	-3.561505934	2.32E-05	Papillary renal cell
160728	-3.436273945	4.88E-08	Papillary renal cell
3242	-3.378359221	5.93E-09	Papillary renal cell
554235	-3.26431113	2.25E-12	Papillary renal cell
146802	-3.12668156	1.30E-06	Papillary renal cell
64902	-3.05286749	2.80E-07	Papillary renal cell
5053	-3.045116035	2.02E-05	Papillary renal cell

118471	-3.038157604	1.50E-05	Papillary renal cell
2538	-2.998071896	1.80E-10	Papillary renal cell
6582	-2.987284264	2.63E-06	Papillary renal cell
8029	-2.986755308	1.43E-09	Papillary renal cell
6514	-2.979195891	2.03E-07	Papillary renal cell
2819	-2.96436673	1.48E-11	Papillary renal cell
5313	-2.959058989	4.35E-07	Papillary renal cell
55586	-2.93209631	1.07E-07	Papillary renal cell
10841	-2.843804714	3.09E-06	Papillary renal cell
3549	-2.766729462	5.22E-10	Papillary renal cell
9027	-2.744625136	3.61E-06	Papillary renal cell
9058	-2.685840405	8.58E-07	Papillary renal cell
257629	-2.6586048	4.02E-07	Papillary renal cell
11181	-2.650123679	1.91E-07	Papillary renal cell
316	-2.647413835	9.35E-08	Papillary renal cell
388595	-2.626471753	7.34E-06	Papillary renal cell
229	-2.624708093	4.24E-06	Papillary renal cell
64850	-2.61044758	3.98E-08	Papillary renal cell
401262	-2.607041155	1.37E-08	Papillary renal cell
10249	-2.589613438	2.14E-05	Papillary renal cell
79814	-2.589569975	8.06E-10	Papillary renal cell
54716	-2.552405162	3.92E-08	Papillary renal cell
339221	-2.515441302	8.86E-07	Papillary renal cell
8424	-2.505740404	5.17E-08	Papillary renal cell
116085	-2.504062014	0.000125153	Papillary renal cell
1610	-2.496642617	1.40E-06	Papillary renal cell
643763	-2.484897738	1.95E-05	Papillary renal cell
7780	-2.458414544	2.64E-06	Papillary renal cell
11148	-2.451520294	1.89E-05	Papillary renal cell
2326	-2.436381818	3.73E-05	Papillary renal cell
290	-2.407385095	1.96E-07	Papillary renal cell
115019	-2.404691483	6.88E-07	Papillary renal cell
283375	-2.396507596	4.24E-05	Papillary renal cell
134288	-2.372731618	2.91E-08	Papillary renal cell
1586	-2.359558753	9.08E-06	Papillary renal cell
2875	-2.321919226	1.60E-10	Papillary renal cell
55244	-2.303092276	9.02E-07	Papillary renal cell
9376	-2.280142595	7.67E-05	Papillary renal cell
220441	-2.278520973	2.79E-10	Papillary renal cell
11136	-2.270564593	8.95E-06	Papillary renal cell
171389	-2.263241286	2.62E-07	Papillary renal cell
18	-2.262101315	3.41E-15	Papillary renal cell
2170	-2.250328145	7.02E-10	Papillary renal cell
246181	-2.22486698	5.40E-12	Papillary renal cell
80157	-2.213469524	0.00010714	Papillary renal cell
4634	-2.206736633	6.61E-06	Papillary renal cell
197257	-2.204196389	1.77E-17	Papillary renal cell
10840	-2.190802597	7.58E-08	Papillary renal cell
389434	-2.181699923	1.12E-07	Papillary renal cell
54546	-2.177636717	7.70E-05	Papillary renal cell
3172	-2.177538573	8.17E-06	Papillary renal cell
2628	-2.175785551	3.43E-08	Papillary renal cell
9390	-2.15167507	1.87E-05	Papillary renal cell
64849	-2.131261987	0.000504106	Papillary renal cell
1579	-2.125803352	0.000207133	Papillary renal cell
57733	-2.093761088	0.000232638	Papillary renal cell
51268	-2.0904576	2.62E-07	Papillary renal cell
5745	-2.086148248	9.73E-07	Papillary renal cell

159963	-2.081812011	0.000182763	Papillary renal cell
284422	-2.077320755	9.82E-06	Papillary renal cell
92292	-2.072596926	7.57E-07	Papillary renal cell
4329	-2.067321155	5.24E-14	Papillary renal cell
83758	-2.057306988	7.88E-07	Papillary renal cell
28999	-2.055286966	3.86E-13	Papillary renal cell
84647	-2.047776959	5.86E-06	Papillary renal cell
9104	-2.011299094	5.75E-09	Papillary renal cell
125206	-1.994516625	0.000104332	Papillary renal cell
55867	-1.988618663	0.000121325	Papillary renal cell
9615	-1.978911681	0.000361958	Papillary renal cell
4311	-1.963263353	6.40E-05	Papillary renal cell
4648	-1.946453869	1.96E-05	Papillary renal cell
81693	-1.938957016	0.000392455	Papillary renal cell
1733	-1.938683353	0.000573344	Papillary renal cell
8659	-1.937866409	3.71E-14	Papillary renal cell
53841	-1.936012122	0.002641168	Papillary renal cell
5121	-1.935315114	0.001371582	Papillary renal cell
2028	-1.92788001	1.64E-06	Papillary renal cell
64922	-1.918405549	6.61E-08	Papillary renal cell
25924	-1.914985677	3.52E-06	Papillary renal cell
3249	-1.911521486	5.74E-06	Papillary renal cell
392465	-1.911418494	1.60E-06	Papillary renal cell
134285	-1.910340239	5.10E-07	Papillary renal cell
9099	-1.904864633	1.90E-09	Papillary renal cell
5284	-1.898402618	3.92E-05	Papillary renal cell
5167	-1.871273245	9.82E-10	Papillary renal cell
55540	-1.870601728	2.79E-08	Papillary renal cell
123264	-1.864534348	6.57E-05	Papillary renal cell
10786	-1.857689976	0.002191554	Papillary renal cell
613212	-1.854049208	3.18E-06	Papillary renal cell
342979	-1.841002402	8.18E-08	Papillary renal cell
1644	-1.839833292	0.000457216	Papillary renal cell
27233	-1.830515559	1.17E-05	Papillary renal cell
83729	-1.821080907	7.26E-05	Papillary renal cell
6359	-1.81893988	0.000306046	Papillary renal cell
348249	-1.815804789	2.84E-05	Papillary renal cell
346606	-1.814522865	0.00012549	Papillary renal cell
83715	-1.807945366	5.02E-06	Papillary renal cell
728939	-1.800023062	2.45E-05	Papillary renal cell
27329	-1.795003448	8.67E-05	Papillary renal cell
23072	-1.788654387	0.000333244	Papillary renal cell
220963	-1.786812183	0.000249604	Papillary renal cell
1807	-1.781455823	0.003150204	Papillary renal cell
55753	-1.77334169	8.81E-08	Papillary renal cell
401250	-1.767137777	0.000334255	Papillary renal cell
56159	-1.76150767	5.88E-05	Papillary renal cell
358	-1.761206628	4.96E-05	Papillary renal cell
115584	-1.742716881	0.000223361	Papillary renal cell
153328	-1.737231119	1.46E-05	Papillary renal cell
100170765	-1.734612585	8.81E-06	Papillary renal cell
135892	-1.733976653	4.26E-05	Papillary renal cell
9071	-1.731643451	1.68E-06	Papillary renal cell
146456	-1.728631695	9.56E-05	Papillary renal cell
3795	-1.725399675	2.64E-06	Papillary renal cell
388387	-1.716267677	0.00089611	Papillary renal cell
4036	-1.706747004	0.00352176	Papillary renal cell
8972	-1.70615248	0.00300608	Papillary renal cell

8110	-1.702189451	4.41E-09	Papillary renal cell
57452	-1.699175074	1.18E-05	Papillary renal cell
38	-1.696405994	3.06E-19	Papillary renal cell
9077	-1.695399578	3.79E-07	Papillary renal cell
79746	-1.688658126	5.20E-06	Papillary renal cell
2939	-1.679146761	0.002392007	Papillary renal cell
51733	-1.664033662	1.31E-05	Papillary renal cell
9154	-1.657708237	0.000676666	Papillary renal cell
368	-1.634040201	1.34E-05	Papillary renal cell
248	-1.632868108	0.001533028	Papillary renal cell
148709	-1.627061029	4.90E-07	Papillary renal cell
152078	-1.626531137	0.000938205	Papillary renal cell
54866	-1.619073921	0.000338626	Papillary renal cell
5003	-1.617394	4.80E-07	Papillary renal cell
388323	-1.61716152	0.000245975	Papillary renal cell
83876	-1.613830483	5.06E-07	Papillary renal cell
84215	-1.612617416	7.89E-05	Papillary renal cell
364	-1.605766489	1.41E-06	Papillary renal cell
1962	-1.593700298	5.85E-13	Papillary renal cell
6540	-1.591033255	0.001165886	Papillary renal cell
130	-1.590039383	0.000979443	Papillary renal cell
123876	-1.588949172	0.006395004	Papillary renal cell
119467	-1.583028907	0.00257521	Papillary renal cell
66002	-1.580928523	0.000753958	Papillary renal cell
58510	-1.579908182	0.008639125	Papillary renal cell
653190	-1.569164894	0.000141449	Papillary renal cell
55349	-1.568885533	1.20E-11	Papillary renal cell
3957	-1.56678735	0.001860984	Papillary renal cell
151295	-1.564855514	3.15E-06	Papillary renal cell
10803	-1.559283406	9.91E-07	Papillary renal cell
189	-1.558866175	1.56E-05	Papillary renal cell
8671	-1.555423208	4.77E-07	Papillary renal cell
91703	-1.54953725	0.000622218	Papillary renal cell
10788	-1.549029895	4.16E-08	Papillary renal cell
10117	-1.546926604	6.90E-08	Papillary renal cell
1152	-1.545752275	7.78E-09	Papillary renal cell
84968	-1.537595828	0.000357187	Papillary renal cell
3626	-1.537497895	3.47E-07	Papillary renal cell
170392	-1.529695506	1.09E-05	Papillary renal cell
4051	-1.526110972	0.004465704	Papillary renal cell
79919	-1.52013875	0.001504311	Papillary renal cell
6569	-1.519662019	0.002073833	Papillary renal cell
53345	-1.519155626	9.48E-05	Papillary renal cell
63924	-1.518020077	1.07E-05	Papillary renal cell
5340	-1.510542097	0.001951196	Papillary renal cell
130013	-1.503146359	0.000137128	Papillary renal cell
283422	1.501238017	0.001912044	Papillary renal cell
56967	1.501383468	1.85E-05	Papillary renal cell
9985	1.502628918	8.54E-06	Papillary renal cell
83540	1.502751673	9.10E-07	Papillary renal cell
6857	1.504119246	0.000105887	Papillary renal cell
5947	1.504523478	5.79E-10	Papillary renal cell
7025	1.505056668	4.95E-06	Papillary renal cell
64221	1.510872143	5.42E-07	Papillary renal cell
144455	1.511118578	4.30E-07	Papillary renal cell
64881	1.511191705	0.002937139	Papillary renal cell
4692	1.511620454	5.32E-06	Papillary renal cell
4192	1.514233066	1.79E-06	Papillary renal cell

6660	1.51487949	3.22E-05	Papillary renal cell
3742	1.517145958	6.81E-09	Papillary renal cell
130132	1.517179389	5.16E-07	Papillary renal cell
2562	1.517323067	0.005460699	Papillary renal cell
130574	1.517912426	8.28E-06	Papillary renal cell
11065	1.519380756	6.98E-07	Papillary renal cell
5837	1.519582813	2.16E-08	Papillary renal cell
92304	1.521242254	1.76E-05	Papillary renal cell
6493	1.521347529	5.06E-09	Papillary renal cell
2906	1.523134906	1.34E-08	Papillary renal cell
112885	1.523285918	3.13E-05	Papillary renal cell
50509	1.523337455	1.07E-05	Papillary renal cell
90853	1.525905781	0.000116181	Papillary renal cell
9212	1.525918624	1.31E-07	Papillary renal cell
90161	1.527530538	0.001478405	Papillary renal cell
286133	1.527999634	5.51E-05	Papillary renal cell
84258	1.528891415	0.000178778	Papillary renal cell
55509	1.529809163	5.63E-12	Papillary renal cell
26084	1.531243316	7.17E-09	Papillary renal cell
114041	1.531256353	1.49E-05	Papillary renal cell
126393	1.531979111	1.14E-05	Papillary renal cell
253738	1.53326255	7.79E-06	Papillary renal cell
114784	1.534031903	1.34E-06	Papillary renal cell
3782	1.53526861	1.00E-05	Papillary renal cell
55806	1.536400612	2.07E-05	Papillary renal cell
5143	1.53779702	5.85E-09	Papillary renal cell
63982	1.538644787	5.00E-06	Papillary renal cell
58189	1.539400102	5.40E-07	Papillary renal cell
10536	1.539782122	4.93E-07	Papillary renal cell
6515	1.540954843	6.99E-08	Papillary renal cell
23650	1.542042522	0.000816505	Papillary renal cell
2019	1.542953131	4.16E-05	Papillary renal cell
55355	1.544101789	1.12E-07	Papillary renal cell
4921	1.545381321	0.0001015	Papillary renal cell
125	1.545766278	0.001805114	Papillary renal cell
7033	1.547943071	8.49E-05	Papillary renal cell
57469	1.548031027	9.36E-09	Papillary renal cell
3280	1.548111575	2.52E-12	Papillary renal cell
283208	1.549100683	1.80E-05	Papillary renal cell
219539	1.549690415	8.11E-09	Papillary renal cell
5136	1.55038523	0.004082424	Papillary renal cell
1298	1.550608292	8.05E-06	Papillary renal cell
8745	1.552040695	4.10E-05	Papillary renal cell
94274	1.553060522	1.69E-08	Papillary renal cell
10900	1.553249147	3.26E-05	Papillary renal cell
146909	1.556421258	1.77E-08	Papillary renal cell
79827	1.556777197	2.66E-05	Papillary renal cell
10578	1.557459596	1.11E-06	Papillary renal cell
9890	1.558174843	2.80E-06	Papillary renal cell
79368	1.559689488	5.66E-07	Papillary renal cell
340547	1.561145093	1.22E-05	Papillary renal cell
55022	1.561596677	1.27E-09	Papillary renal cell
56917	1.562498663	3.53E-05	Papillary renal cell
54739	1.562908026	1.58E-08	Papillary renal cell
79616	1.563093232	0.00023272	Papillary renal cell
5266	1.563518416	0.001653414	Papillary renal cell
7472	1.564569216	0.000275168	Papillary renal cell
5153	1.569107099	4.85E-07	Papillary renal cell

3800	1.569482176	3.22E-08	Papillary renal cell
10267	1.569511383	5.81E-06	Papillary renal cell
930	1.569747382	1.34E-05	Papillary renal cell
23127	1.570224512	6.89E-07	Papillary renal cell
23149	1.570315268	8.26E-08	Papillary renal cell
25893	1.573407257	2.40E-05	Papillary renal cell
23025	1.575636772	1.93E-05	Papillary renal cell
29774	1.575736196	2.83E-10	Papillary renal cell
27319	1.575764292	3.45E-07	Papillary renal cell
59	1.577387627	4.78E-11	Papillary renal cell
2615	1.577694767	3.75E-14	Papillary renal cell
1909	1.578407765	1.86E-08	Papillary renal cell
2070	1.579393435	4.35E-05	Papillary renal cell
4010	1.579463564	5.92E-05	Papillary renal cell
6915	1.580615074	2.41E-10	Papillary renal cell
150365	1.582556405	1.50E-09	Papillary renal cell
5054	1.584092235	5.97E-06	Papillary renal cell
8111	1.584766506	1.18E-12	Papillary renal cell
767811	1.585902056	7.86E-10	Papillary renal cell
2583	1.586483837	0.000174207	Papillary renal cell
2020	1.587267676	0.000331621	Papillary renal cell
187	1.587838797	3.25E-05	Papillary renal cell
4804	1.587962566	6.67E-07	Papillary renal cell
23017	1.588385296	1.37E-07	Papillary renal cell
1490	1.590938581	3.23E-10	Papillary renal cell
1290	1.591181058	4.44E-09	Papillary renal cell
6091	1.592907894	8.27E-07	Papillary renal cell
10256	1.594030705	0.003104756	Papillary renal cell
1469	1.595256376	9.09E-05	Papillary renal cell
151354	1.595461641	5.46E-09	Papillary renal cell
5744	1.595490508	7.28E-07	Papillary renal cell
91851	1.596067205	0.004746849	Papillary renal cell
645369	1.596120265	1.36E-06	Papillary renal cell
23627	1.596394456	5.08E-07	Papillary renal cell
140886	1.596440687	5.44E-06	Papillary renal cell
79987	1.596763844	7.13E-05	Papillary renal cell
10551	1.597425235	0.006252475	Papillary renal cell
6405	1.598072903	2.23E-10	Papillary renal cell
81607	1.600282326	0.000566786	Papillary renal cell
145270	1.600627052	5.88E-05	Papillary renal cell
2977	1.601431892	7.22E-07	Papillary renal cell
79625	1.602346899	0.000118913	Papillary renal cell
283316	1.603360634	1.03E-05	Papillary renal cell
57822	1.604421514	0.000220796	Papillary renal cell
284	1.605728782	4.37E-06	Papillary renal cell
8507	1.607052445	3.18E-14	Papillary renal cell
5327	1.607589969	8.15E-13	Papillary renal cell
56978	1.610141305	1.59E-08	Papillary renal cell
57030	1.611973768	1.04E-09	Papillary renal cell
389840	1.611984886	0.00022543	Papillary renal cell
27063	1.612189861	4.14E-05	Papillary renal cell
56849	1.612794475	3.62E-08	Papillary renal cell
8510	1.613817567	3.91E-10	Papillary renal cell
3090	1.615455684	1.15E-13	Papillary renal cell
5577	1.617297786	1.90E-09	Papillary renal cell
118738	1.61826664	6.97E-09	Papillary renal cell
79734	1.618421196	3.22E-11	Papillary renal cell
6999	1.618674046	9.74E-06	Papillary renal cell

861	1.61895652	1.33E-10	Papillary renal cell
4776	1.619683333	1.84E-08	Papillary renal cell
8497	1.619820175	1.56E-06	Papillary renal cell
6586	1.620048934	7.81E-08	Papillary renal cell
131034	1.62031979	0.00339134	Papillary renal cell
222962	1.620797854	5.00E-06	Papillary renal cell
203447	1.62084285	0.003103163	Papillary renal cell
286046	1.621681849	3.33E-06	Papillary renal cell
80307	1.624528237	0.000222787	Papillary renal cell
57338	1.624944274	6.40E-07	Papillary renal cell
4605	1.625977727	6.41E-08	Papillary renal cell
57333	1.626210838	8.78E-16	Papillary renal cell
150	1.626711314	7.86E-08	Papillary renal cell
196740	1.627288438	7.10E-09	Papillary renal cell
9118	1.627391805	0.000116403	Papillary renal cell
8572	1.632471358	8.12E-06	Papillary renal cell
1307	1.633882749	1.16E-09	Papillary renal cell
60468	1.635194798	1.54E-07	Papillary renal cell
120114	1.635802648	0.000179338	Papillary renal cell
29881	1.635894518	0.002043944	Papillary renal cell
4886	1.636785866	1.91E-09	Papillary renal cell
1903	1.638467627	1.02E-09	Papillary renal cell
26047	1.63907782	9.57E-08	Papillary renal cell
140597	1.639916634	4.34E-05	Papillary renal cell
7053	1.640121465	1.24E-05	Papillary renal cell
4070	1.641211053	0.008387596	Papillary renal cell
246213	1.642046689	5.55E-06	Papillary renal cell
6474	1.645302102	9.01E-07	Papillary renal cell
10335	1.645707892	1.08E-08	Papillary renal cell
114898	1.646367128	1.23E-09	Papillary renal cell
51316	1.646923876	4.55E-08	Papillary renal cell
114757	1.647957936	4.11E-11	Papillary renal cell
148170	1.648318003	1.29E-08	Papillary renal cell
171024	1.649632151	4.37E-08	Papillary renal cell
56154	1.64975951	1.87E-05	Papillary renal cell
81578	1.65205942	1.47E-05	Papillary renal cell
2066	1.652896591	0.001980746	Papillary renal cell
4240	1.655220627	9.86E-13	Papillary renal cell
70	1.655553557	9.88E-05	Papillary renal cell
7020	1.656037486	0.00190222	Papillary renal cell
5655	1.656805963	2.91E-05	Papillary renal cell
1960	1.656845823	7.91E-07	Papillary renal cell
85016	1.657168744	9.87E-06	Papillary renal cell
84929	1.657706121	0.00017419	Papillary renal cell
8324	1.657732531	6.42E-12	Papillary renal cell
83468	1.657928745	2.20E-08	Papillary renal cell
89792	1.65926984	0.000986917	Papillary renal cell
387695	1.659706285	0.001216483	Papillary renal cell
970	1.660557199	0.00184585	Papillary renal cell
654	1.661326664	1.21E-08	Papillary renal cell
116535	1.66290088	1.80E-06	Papillary renal cell
57419	1.662985078	1.71E-07	Papillary renal cell
2687	1.664062906	1.04E-08	Papillary renal cell
253827	1.665442951	5.67E-11	Papillary renal cell
4854	1.665535073	2.38E-07	Papillary renal cell
25878	1.66772663	2.10E-05	Papillary renal cell
56896	1.669162718	0.000123278	Papillary renal cell
259266	1.669896457	1.07E-06	Papillary renal cell

420	1.67057721	1.46E-06	Papillary renal cell
84417	1.671015334	0.000109346	Papillary renal cell
931	1.671373946	3.39E-05	Papillary renal cell
782	1.67185786	1.40E-13	Papillary renal cell
23462	1.672234012	7.93E-10	Papillary renal cell
63910	1.673553476	1.76E-09	Papillary renal cell
3229	1.673566763	1.86E-05	Papillary renal cell
54898	1.67383331	5.13E-07	Papillary renal cell
11095	1.673891718	3.34E-11	Papillary renal cell
113278	1.674328008	0.000734251	Papillary renal cell
3910	1.674606739	1.66E-09	Papillary renal cell
25790	1.675759018	8.25E-07	Papillary renal cell
2303	1.677404409	2.28E-08	Papillary renal cell
57636	1.678394956	4.50E-12	Papillary renal cell
7070	1.678784076	1.05E-08	Papillary renal cell
6442	1.681622353	5.42E-07	Papillary renal cell
51339	1.684344584	3.15E-11	Papillary renal cell
283298	1.68532343	9.68E-08	Papillary renal cell
658	1.68879191	0.001077271	Papillary renal cell
6489	1.689225197	4.04E-08	Papillary renal cell
5350	1.690400987	3.11E-06	Papillary renal cell
57616	1.690639772	9.86E-14	Papillary renal cell
952	1.691270504	9.65E-07	Papillary renal cell
66000	1.6913003	1.53E-07	Papillary renal cell
54361	1.693096721	3.09E-06	Papillary renal cell
8787	1.693878275	3.05E-05	Papillary renal cell
8501	1.693956727	1.99E-07	Papillary renal cell
7041	1.694756767	1.69E-10	Papillary renal cell
744	1.696223715	3.29E-06	Papillary renal cell
54551	1.698868203	5.87E-08	Papillary renal cell
8515	1.70066786	3.82E-10	Papillary renal cell
254228	1.701164761	2.14E-09	Papillary renal cell
164832	1.701367031	2.55E-06	Papillary renal cell
90246	1.701780165	4.94E-06	Papillary renal cell
2275	1.703061319	8.18E-21	Papillary renal cell
1879	1.70354399	1.22E-09	Papillary renal cell
273	1.705306282	5.16E-07	Papillary renal cell
83879	1.705851478	3.14E-08	Papillary renal cell
26575	1.706681151	4.80E-08	Papillary renal cell
9001	1.706945788	6.71E-08	Papillary renal cell
201633	1.707473769	1.67E-07	Papillary renal cell
23114	1.708366694	1.03E-09	Papillary renal cell
337876	1.70888887	1.56E-10	Papillary renal cell
644353	1.709376306	8.29E-13	Papillary renal cell
26298	1.711908075	0.000915372	Papillary renal cell
4884	1.712523677	8.48E-07	Papillary renal cell
153572	1.713483829	2.69E-05	Papillary renal cell
653145	1.715433481	1.57E-05	Papillary renal cell
10076	1.716901554	1.31E-05	Papillary renal cell
8519	1.719351361	2.05E-12	Papillary renal cell
441094	1.719508607	7.55E-09	Papillary renal cell
2302	1.722636752	0.000253946	Papillary renal cell
25999	1.723162228	1.80E-07	Papillary renal cell
1602	1.72588797	9.25E-05	Papillary renal cell
4062	1.726144794	3.53E-08	Papillary renal cell
8482	1.727258166	1.22E-12	Papillary renal cell
100188953	1.728589852	1.84E-10	Papillary renal cell
3290	1.728775276	2.12E-07	Papillary renal cell

347902	1.729648393	2.44E-07	Papillary renal cell
171177	1.730028997	4.56E-05	Papillary renal cell
93649	1.731910795	1.12E-07	Papillary renal cell
64399	1.733021499	2.56E-06	Papillary renal cell
5739	1.734855338	1.10E-11	Papillary renal cell
5754	1.735486496	2.14E-08	Papillary renal cell
956	1.735880517	2.44E-06	Papillary renal cell
643	1.737239995	1.38E-07	Papillary renal cell
7850	1.737790866	5.27E-06	Papillary renal cell
2719	1.739868172	1.63E-06	Papillary renal cell
126567	1.740243998	9.53E-08	Papillary renal cell
81794	1.740442716	1.29E-11	Papillary renal cell
1287	1.740554159	2.21E-06	Papillary renal cell
1306	1.742795392	4.71E-09	Papillary renal cell
28954	1.744052093	3.70E-11	Papillary renal cell
2048	1.745331957	1.52E-10	Papillary renal cell
2049	1.747727632	9.46E-09	Papillary renal cell
51148	1.75353839	3.66E-12	Papillary renal cell
2304	1.755142622	1.98E-05	Papillary renal cell
126668	1.760209083	8.41E-07	Papillary renal cell
145820	1.764131216	2.90E-09	Papillary renal cell
9452	1.764758594	1.02E-08	Papillary renal cell
11173	1.765047701	1.16E-14	Papillary renal cell
7223	1.765216987	1.58E-10	Papillary renal cell
4969	1.767228587	0.000114828	Papillary renal cell
1404	1.76836559	2.25E-06	Papillary renal cell
8854	1.774472389	0.001149026	Papillary renal cell
312	1.775318618	0.004397317	Papillary renal cell
2202	1.784977497	2.80E-05	Papillary renal cell
5159	1.785590112	2.30E-11	Papillary renal cell
2199	1.78620625	1.40E-09	Papillary renal cell
219348	1.788365556	3.90E-07	Papillary renal cell
909	1.788378807	6.50E-07	Papillary renal cell
340075	1.790279025	3.53E-05	Papillary renal cell
64220	1.790805789	0.000204922	Papillary renal cell
54511	1.792863991	8.06E-06	Papillary renal cell
147495	1.795681536	4.86E-08	Papillary renal cell
4916	1.796421314	7.80E-06	Papillary renal cell
730	1.797328549	0.000809412	Papillary renal cell
221421	1.797573149	2.55E-07	Papillary renal cell
2707	1.802167962	4.58E-05	Papillary renal cell
80128	1.804205846	2.42E-10	Papillary renal cell
79816	1.804605619	2.03E-06	Papillary renal cell
8534	1.806960069	2.93E-11	Papillary renal cell
84433	1.807741885	6.13E-11	Papillary renal cell
79883	1.808706075	2.99E-12	Papillary renal cell
161291	1.809479967	0.000325879	Papillary renal cell
1346	1.809561039	8.77E-05	Papillary renal cell
54510	1.811753895	2.07E-08	Papillary renal cell
3897	1.811766229	0.004229498	Papillary renal cell
775	1.812739322	1.26E-10	Papillary renal cell
3662	1.815095044	8.59E-08	Papillary renal cell
80117	1.817505326	7.88E-06	Papillary renal cell
6545	1.818795906	5.35E-05	Papillary renal cell
1300	1.81989495	1.01E-05	Papillary renal cell
167681	1.821032865	1.64E-07	Papillary renal cell
4248	1.821414714	4.52E-08	Papillary renal cell
285973	1.821535795	9.77E-10	Papillary renal cell

4629	1.822137003	1.47E-06	Papillary renal cell
284656	1.823792379	0.000105262	Papillary renal cell
22943	1.827462251	1.53E-08	Papillary renal cell
222663	1.82811663	1.35E-07	Papillary renal cell
8642	1.8330367	2.46E-18	Papillary renal cell
4239	1.8339803	5.74E-06	Papillary renal cell
25960	1.834709146	2.72E-12	Papillary renal cell
2894	1.837342725	6.70E-05	Papillary renal cell
127534	1.840326983	0.000156951	Papillary renal cell
51676	1.841412724	1.65E-11	Papillary renal cell
6591	1.842460052	2.91E-11	Papillary renal cell
2307	1.842590059	1.41E-11	Papillary renal cell
2523	1.842685334	2.49E-11	Papillary renal cell
57689	1.843427	3.12E-06	Papillary renal cell
80119	1.844592331	1.33E-11	Papillary renal cell
6615	1.845106013	4.96E-11	Papillary renal cell
3674	1.846734926	1.51E-08	Papillary renal cell
2243	1.849312239	0.000440068	Papillary renal cell
5133	1.852466479	2.13E-09	Papillary renal cell
4053	1.852617881	1.77E-09	Papillary renal cell
147906	1.854108359	4.45E-13	Papillary renal cell
3755	1.854439994	4.00E-06	Papillary renal cell
169611	1.85444028	5.40E-13	Papillary renal cell
3902	1.85453619	9.91E-10	Papillary renal cell
85407	1.855791372	2.43E-11	Papillary renal cell
1734	1.85750423	2.21E-09	Papillary renal cell
285382	1.858659617	4.14E-12	Papillary renal cell
116328	1.860158172	1.22E-07	Papillary renal cell
151887	1.865130904	5.40E-08	Papillary renal cell
2625	1.865752268	0.000116541	Papillary renal cell
25802	1.867191194	4.59E-08	Papillary renal cell
57124	1.873843644	1.29E-11	Papillary renal cell
7169	1.875060176	2.11E-14	Papillary renal cell
255743	1.879303355	8.02E-05	Papillary renal cell
338773	1.880031275	2.00E-10	Papillary renal cell
26002	1.882905486	1.96E-09	Papillary renal cell
80332	1.885302275	5.89E-07	Papillary renal cell
145864	1.885325267	4.17E-13	Papillary renal cell
25984	1.88862965	0.000198612	Papillary renal cell
222008	1.888826276	0.004812272	Papillary renal cell
2042	1.889187198	3.35E-05	Papillary renal cell
9843	1.889887684	5.76E-11	Papillary renal cell
3699	1.89021701	1.99E-06	Papillary renal cell
25903	1.890357847	1.54E-12	Papillary renal cell
5365	1.890751725	2.78E-06	Papillary renal cell
1015	1.891250833	0.000267717	Papillary renal cell
4325	1.8923286	1.38E-08	Papillary renal cell
7291	1.893299889	1.69E-10	Papillary renal cell
3569	1.893339963	0.000108657	Papillary renal cell
9806	1.89645739	2.91E-12	Papillary renal cell
57699	1.896788661	4.37E-08	Papillary renal cell
8091	1.900187889	3.74E-05	Papillary renal cell
3399	1.900498495	1.06E-15	Papillary renal cell
25907	1.906354323	9.23E-12	Papillary renal cell
5743	1.906378669	1.36E-07	Papillary renal cell
3955	1.911203479	1.02E-10	Papillary renal cell
140628	1.913135549	5.38E-08	Papillary renal cell
10123	1.913379739	1.55E-10	Papillary renal cell

441631	1.913988161	2.43E-08	Papillary renal cell
2784	1.914959137	2.12E-10	Papillary renal cell
1846	1.916694133	1.61E-06	Papillary renal cell
6876	1.917098016	1.49E-10	Papillary renal cell
26579	1.919827208	1.06E-07	Papillary renal cell
1288	1.92305321	1.50E-05	Papillary renal cell
1809	1.923600513	1.41E-10	Papillary renal cell
6263	1.925930403	7.24E-05	Papillary renal cell
8646	1.928844407	5.34E-08	Papillary renal cell
114905	1.92902607	7.21E-08	Papillary renal cell
4889	1.930668106	2.98E-08	Papillary renal cell
3485	1.934760693	1.77E-12	Papillary renal cell
84935	1.935715918	2.88E-09	Papillary renal cell
5730	1.938517777	0.00079124	Papillary renal cell
51334	1.941824909	5.41E-10	Papillary renal cell
9358	1.943570111	6.31E-07	Papillary renal cell
6331	1.944410731	2.77E-08	Papillary renal cell
84525	1.94462908	4.40E-12	Papillary renal cell
10290	1.945852203	5.24E-09	Papillary renal cell
2192	1.947563869	2.09E-09	Papillary renal cell
90381	1.949047125	1.20E-15	Papillary renal cell
65012	1.950028756	1.82E-10	Papillary renal cell
8728	1.952824883	1.32E-12	Papillary renal cell
64093	1.952915694	2.98E-05	Papillary renal cell
2949	1.959893321	1.67E-08	Papillary renal cell
50489	1.961330456	1.16E-06	Papillary renal cell
79974	1.961721215	5.66E-11	Papillary renal cell
57795	1.96214431	3.92E-07	Papillary renal cell
81792	1.963595926	1.66E-08	Papillary renal cell
2568	1.965410618	1.76E-06	Papillary renal cell
53820	1.965553235	3.71E-08	Papillary renal cell
50615	1.965860017	5.60E-10	Papillary renal cell
5653	1.966086618	4.40E-05	Papillary renal cell
23284	1.966761954	3.85E-07	Papillary renal cell
3237	1.96909694	1.58E-05	Papillary renal cell
28514	1.970036498	6.33E-09	Papillary renal cell
55190	1.973637129	5.96E-10	Papillary renal cell
170685	1.973654749	2.97E-10	Papillary renal cell
6495	1.973824153	2.33E-07	Papillary renal cell
2735	1.974619402	1.16E-12	Papillary renal cell
93145	1.974702014	2.55E-07	Papillary renal cell
56649	1.975129064	0.001246006	Papillary renal cell
55384	1.982303936	9.83E-10	Papillary renal cell
4099	1.983409702	1.06E-06	Papillary renal cell
80757	1.984554942	9.50E-10	Papillary renal cell
72	1.98474553	3.15E-05	Papillary renal cell
54829	1.985607443	1.94E-09	Papillary renal cell
79776	1.985872582	4.22E-05	Papillary renal cell
387763	1.988465931	1.55E-10	Papillary renal cell
7104	1.991071675	0.000101753	Papillary renal cell
2118	1.999401067	1.63E-06	Papillary renal cell
10846	1.999734647	9.66E-06	Papillary renal cell
27286	2.000570921	1.61E-06	Papillary renal cell
2564	2.001611658	1.31E-07	Papillary renal cell
5241	2.003979141	3.97E-07	Papillary renal cell
7293	2.004057663	1.85E-12	Papillary renal cell
83700	2.005120205	1.73E-15	Papillary renal cell
85352	2.00751278	4.15E-07	Papillary renal cell

5549	2.010346903	1.77E-08	Papillary renal cell
608	2.010517162	2.43E-07	Papillary renal cell
1301	2.01272053	1.70E-06	Papillary renal cell
1805	2.013924612	2.61E-09	Papillary renal cell
144347	2.014319599	1.09E-07	Papillary renal cell
1356	2.016034255	9.87E-05	Papillary renal cell
80243	2.017686216	3.15E-06	Papillary renal cell
1946	2.02035552	1.23E-08	Papillary renal cell
54039	2.020983506	7.69E-07	Papillary renal cell
4256	2.023283643	8.75E-10	Papillary renal cell
5046	2.023569683	1.25E-10	Papillary renal cell
115908	2.023923386	1.14E-09	Papillary renal cell
126129	2.024455594	1.28E-12	Papillary renal cell
1002	2.026636549	0.000288542	Papillary renal cell
259232	2.028978723	2.39E-07	Papillary renal cell
799	2.03082764	3.93E-10	Papillary renal cell
3547	2.037209334	5.62E-06	Papillary renal cell
387856	2.038587595	7.45E-14	Papillary renal cell
338761	2.040673735	3.79E-05	Papillary renal cell
27147	2.042008554	5.58E-09	Papillary renal cell
57214	2.042523018	7.55E-07	Papillary renal cell
2786	2.044766185	6.07E-07	Papillary renal cell
96610	2.044941535	1.16E-06	Papillary renal cell
728392	2.045661417	1.99E-10	Papillary renal cell
23432	2.04719243	2.31E-17	Papillary renal cell
55359	2.047517713	9.57E-07	Papillary renal cell
154664	2.049394451	5.93E-05	Papillary renal cell
24	2.04954896	1.87E-09	Papillary renal cell
3075	2.050272492	4.08E-08	Papillary renal cell
55118	2.05032267	0.000699389	Papillary renal cell
349136	2.052668191	3.24E-10	Papillary renal cell
7225	2.054008682	1.90E-10	Papillary renal cell
1294	2.062096995	5.52E-07	Papillary renal cell
11117	2.062343934	9.54E-12	Papillary renal cell
23057	2.065458567	1.27E-08	Papillary renal cell
1462	2.067163234	2.53E-08	Papillary renal cell
57111	2.067666568	0.004089642	Papillary renal cell
4143	2.069415591	0.000110147	Papillary renal cell
146206	2.069998245	5.41E-11	Papillary renal cell
389136	2.073605748	4.93E-07	Papillary renal cell
90993	2.075075637	3.22E-05	Papillary renal cell
94031	2.081007966	1.10E-13	Papillary renal cell
8516	2.08231457	1.30E-06	Papillary renal cell
22801	2.087345617	3.79E-09	Papillary renal cell
5317	2.087698883	1.41E-07	Papillary renal cell
652995	2.088371167	0.00011523	Papillary renal cell
230	2.088831772	8.85E-09	Papillary renal cell
9834	2.089067973	1.61E-08	Papillary renal cell
114899	2.097633691	1.42E-07	Papillary renal cell
6853	2.101027564	1.06E-10	Papillary renal cell
10350	2.101913149	1.59E-07	Papillary renal cell
1305	2.103170982	3.62E-10	Papillary renal cell
51176	2.111344643	4.25E-11	Papillary renal cell
221393	2.116237141	1.28E-07	Papillary renal cell
3741	2.120134763	1.63E-09	Papillary renal cell
2152	2.122054776	6.20E-13	Papillary renal cell
10439	2.127061776	2.22E-06	Papillary renal cell
9892	2.129828377	1.68E-06	Papillary renal cell

55107	2.131816869	1.05E-11	Papillary renal cell
9900	2.138558724	1.17E-10	Papillary renal cell
148741	2.14455499	2.65E-11	Papillary renal cell
2775	2.151338607	5.28E-10	Papillary renal cell
147381	2.153121317	1.28E-07	Papillary renal cell
6943	2.153565872	6.04E-08	Papillary renal cell
1470	2.160538384	2.48E-08	Papillary renal cell
65268	2.160746884	3.07E-05	Papillary renal cell
91319	2.161558424	1.72E-12	Papillary renal cell
2006	2.173577798	3.48E-11	Papillary renal cell
1295	2.176062673	2.66E-10	Papillary renal cell
3624	2.177818449	1.46E-09	Papillary renal cell
284069	2.179313363	3.97E-12	Papillary renal cell
2563	2.180302746	7.20E-10	Papillary renal cell
1674	2.184275899	3.03E-05	Papillary renal cell
728264	2.187230766	9.02E-12	Papillary renal cell
79413	2.189390245	6.11E-07	Papillary renal cell
64699	2.189751678	1.02E-06	Papillary renal cell
4684	2.190480799	1.21E-07	Papillary renal cell
63895	2.190933693	1.38E-08	Papillary renal cell
6363	2.194649482	1.82E-05	Papillary renal cell
11096	2.195132812	3.29E-09	Papillary renal cell
8701	2.198796849	1.58E-07	Papillary renal cell
645784	2.207705984	1.72E-08	Papillary renal cell
3872	2.21062612	0.000282629	Papillary renal cell
10563	2.213059636	1.16E-05	Papillary renal cell
84870	2.220954551	1.53E-08	Papillary renal cell
3357	2.222410827	5.04E-10	Papillary renal cell
10637	2.223306658	6.16E-06	Papillary renal cell
84628	2.223391623	7.00E-13	Papillary renal cell
441869	2.227268893	9.23E-09	Papillary renal cell
27443	2.239224622	2.97E-07	Papillary renal cell
116154	2.240019135	1.58E-05	Papillary renal cell
1278	2.244839965	2.10E-12	Papillary renal cell
51308	2.248390455	1.37E-10	Papillary renal cell
1114	2.25096533	3.72E-08	Papillary renal cell
92745	2.252408368	1.52E-08	Papillary renal cell
4440	2.260256157	3.19E-11	Papillary renal cell
2297	2.269418009	6.33E-08	Papillary renal cell
57595	2.271534494	3.24E-16	Papillary renal cell
196500	2.278626445	4.01E-12	Papillary renal cell
90113	2.28215817	9.99E-10	Papillary renal cell
94030	2.285391791	2.89E-12	Papillary renal cell
4885	2.289295273	2.53E-09	Papillary renal cell
83416	2.290167521	1.75E-07	Papillary renal cell
10570	2.291005127	3.85E-08	Papillary renal cell
9383	2.292282361	0.000123966	Papillary renal cell
1264	2.306352719	3.21E-09	Papillary renal cell
24141	2.312839261	1.85E-06	Papillary renal cell
8038	2.317721206	1.15E-12	Papillary renal cell
23416	2.320093199	5.59E-07	Papillary renal cell
6422	2.322449137	2.92E-06	Papillary renal cell
10371	2.330672427	4.41E-10	Papillary renal cell
57593	2.33594512	8.94E-11	Papillary renal cell
256472	2.337849663	4.85E-10	Papillary renal cell
3481	2.338802262	2.64E-07	Papillary renal cell
55698	2.34038585	4.83E-10	Papillary renal cell
65997	2.344854557	3.13E-11	Papillary renal cell

127435	2.345692671	4.33E-13	Papillary renal cell
5396	2.349109993	2.35E-09	Papillary renal cell
128553	2.35242422	3.93E-11	Papillary renal cell
57502	2.35296065	4.55E-08	Papillary renal cell
6447	2.356251482	1.11E-06	Papillary renal cell
10683	2.361884786	2.53E-11	Papillary renal cell
165215	2.362117254	6.09E-10	Papillary renal cell
116039	2.364859045	1.40E-08	Papillary renal cell
10242	2.372244793	8.59E-09	Papillary renal cell
9096	2.374596552	2.17E-08	Papillary renal cell
1001	2.374826462	3.70E-07	Papillary renal cell
5099	2.380431503	5.44E-08	Papillary renal cell
8839	2.385577803	5.85E-09	Papillary renal cell
1634	2.396675376	1.11E-08	Papillary renal cell
2244	2.397547108	0.000535688	Papillary renal cell
80326	2.401866253	2.99E-08	Papillary renal cell
50863	2.408026707	3.03E-10	Papillary renal cell
3397	2.413030643	1.18E-11	Papillary renal cell
338707	2.415141464	2.13E-06	Papillary renal cell
6366	2.417485506	4.98E-05	Papillary renal cell
5450	2.42342069	4.80E-07	Papillary renal cell
655	2.428168162	1.26E-05	Papillary renal cell
55076	2.434334673	5.42E-13	Papillary renal cell
1268	2.43578821	7.72E-09	Papillary renal cell
1277	2.439297768	3.33E-14	Papillary renal cell
633	2.441851898	1.42E-18	Papillary renal cell
63876	2.449806136	1.31E-13	Papillary renal cell
8406	2.453242105	1.00E-09	Papillary renal cell
176	2.457125834	4.14E-08	Papillary renal cell
84620	2.470855626	3.41E-07	Papillary renal cell
1292	2.477430391	1.40E-15	Papillary renal cell
1281	2.484084139	1.59E-12	Papillary renal cell
165	2.484518623	3.65E-09	Papillary renal cell
221883	2.48681798	1.27E-09	Papillary renal cell
2300	2.490380717	1.23E-16	Papillary renal cell
6785	2.499479592	2.84E-10	Papillary renal cell
1293	2.501431581	3.28E-12	Papillary renal cell
7373	2.508082688	1.41E-11	Papillary renal cell
60681	2.5176587	1.28E-11	Papillary renal cell
5740	2.522797932	5.91E-11	Papillary renal cell
460	2.526336655	1.92E-07	Papillary renal cell
23768	2.533614574	7.57E-13	Papillary renal cell
9902	2.539360746	2.00E-19	Papillary renal cell
64405	2.547552227	7.41E-06	Papillary renal cell
1311	2.55790697	3.98E-07	Papillary renal cell
3671	2.564999813	1.61E-10	Papillary renal cell
149461	2.568011018	2.65E-07	Papillary renal cell
84856	2.568296089	3.32E-07	Papillary renal cell
222865	2.571364885	3.13E-06	Papillary renal cell
5999	2.571545857	6.73E-13	Papillary renal cell
51450	2.571579986	4.57E-14	Papillary renal cell
25817	2.572720114	2.66E-08	Papillary renal cell
51237	2.580398986	1.45E-07	Papillary renal cell
286204	2.584285446	3.58E-09	Papillary renal cell
50649	2.587105874	7.93E-11	Papillary renal cell
204962	2.594006561	1.94E-05	Papillary renal cell
2295	2.597248848	3.55E-13	Papillary renal cell
55061	2.605358321	9.36E-12	Papillary renal cell

973	2.607399498	7.49E-09	Papillary renal cell
388610	2.610568769	2.57E-08	Papillary renal cell
84624	2.614927447	2.14E-10	Papillary renal cell
221806	2.624363085	5.27E-09	Papillary renal cell
29942	2.628713547	1.46E-09	Papillary renal cell
29943	2.631414964	5.10E-12	Papillary renal cell
392617	2.633632594	7.50E-09	Papillary renal cell
26585	2.633763435	7.81E-08	Papillary renal cell
4060	2.638751189	2.46E-08	Papillary renal cell
7138	2.645874086	6.23E-07	Papillary renal cell
1308	2.651184602	1.00E-06	Papillary renal cell
10631	2.656341929	1.56E-08	Papillary renal cell
8532	2.671114244	9.60E-12	Papillary renal cell
3207	2.674545394	6.89E-11	Papillary renal cell
5156	2.68319598	6.96E-10	Papillary renal cell
23213	2.690989768	6.04E-14	Papillary renal cell
6335	2.699183454	9.40E-13	Papillary renal cell
5157	2.699305861	1.42E-11	Papillary renal cell
6750	2.701879084	0.000361832	Papillary renal cell
8784	2.706400919	1.12E-13	Papillary renal cell
9244	2.709774839	7.36E-11	Papillary renal cell
1949	2.719606693	1.84E-09	Papillary renal cell
3512	2.723784035	1.85E-06	Papillary renal cell
374654	2.727060805	8.47E-23	Papillary renal cell
5010	2.73329793	3.69E-15	Papillary renal cell
5348	2.733714173	1.73E-12	Papillary renal cell
4920	2.738115711	3.25E-10	Papillary renal cell
2736	2.747800015	9.82E-20	Papillary renal cell
3776	2.767163257	2.48E-13	Papillary renal cell
84251	2.812579476	1.78E-15	Papillary renal cell
1289	2.818241767	1.21E-16	Papillary renal cell
333	2.835543311	9.96E-14	Papillary renal cell
7503	2.842909261	0.000946305	Papillary renal cell
25789	2.878287666	3.89E-15	Papillary renal cell
10149	2.889618329	1.83E-11	Papillary renal cell
6423	2.909407381	7.77E-07	Papillary renal cell
4237	2.923364178	1.13E-12	Papillary renal cell
10278	2.924324911	1.19E-12	Papillary renal cell
56265	3.036745396	5.51E-12	Papillary renal cell
2901	3.053029993	4.97E-09	Papillary renal cell
2191	3.066817695	3.25E-15	Papillary renal cell
1009	3.068721692	2.11E-13	Papillary renal cell
8755	3.096791009	1.35E-07	Papillary renal cell
57863	3.213487639	6.78E-07	Papillary renal cell
4188	3.224078672	2.10E-15	Papillary renal cell
57631	3.240348203	7.16E-16	Papillary renal cell
728239	3.47724976	1.68E-13	Papillary renal cell
728215	3.540566279	2.49E-14	Papillary renal cell
81557	3.598283855	2.52E-14	Papillary renal cell
148398	3.801427956	1.16E-16	Papillary renal cell
1576	-5.364872846	1.04E-13	Liver
10249	-5.13861276	4.50E-18	Liver
1582	-5.074481577	2.27E-19	Liver
1543	-4.821901028	3.98E-15	Liver
1544	-4.821301477	6.48E-12	Liver
9506	-4.506596419	5.79E-12	Liver
366	-4.399585982	1.44E-21	Liver
3242	-4.380219331	1.49E-13	Liver

6554	-4.12295746	6.75E-14	Liver
6506	-4.100333195	2.06E-17	Liver
143941	-4.083302916	1.36E-14	Liver
2998	-4.070997596	4.21E-15	Liver
6898	-4.059696546	6.83E-14	Liver
346	-4.029889709	4.90E-18	Liver
54657	-3.970592997	5.06E-12	Liver
83597	-3.912069052	5.78E-16	Liver
54659	-3.910565098	6.47E-15	Liver
1548	-3.907027022	2.10E-08	Liver
6360	-3.896526051	1.05E-14	Liver
10840	-3.889277272	5.49E-17	Liver
389932	-3.790475365	2.83E-20	Liver
554235	-3.777644671	6.32E-16	Liver
1828	-3.753305041	9.54E-16	Liver
462	-3.629277887	6.91E-14	Liver
3290	-3.600149375	1.42E-13	Liver
8647	-3.588317514	1.98E-12	Liver
64388	-3.578841609	1.85E-10	Liver
10998	-3.461843071	6.90E-22	Liver
6716	-3.447650784	1.09E-15	Liver
123876	-3.444292778	7.67E-15	Liver
255167	-3.397875509	9.52E-09	Liver
202299	-3.396923057	4.51E-08	Liver
218	-3.393348366	3.00E-06	Liver
1581	-3.380148969	3.79E-08	Liver
147111	-3.369107035	3.45E-08	Liver
25924	-3.364516317	4.72E-16	Liver
7365	-3.360402942	6.91E-14	Liver
5105	-3.359308994	1.26E-09	Liver
6822	-3.355044607	1.35E-11	Liver
127	-3.349114801	8.11E-09	Liver
54988	-3.336435103	6.11E-17	Liver
635	-3.321188806	1.27E-12	Liver
353299	-3.291564852	5.94E-12	Liver
348158	-3.26115043	3.87E-15	Liver
9970	-3.246572435	5.21E-19	Liver
1373	-3.245737975	7.67E-09	Liver
3250	-3.22785596	1.64E-12	Liver
125	-3.195175703	2.01E-13	Liver
5009	-3.19426643	4.60E-10	Liver
5207	-3.158606469	4.39E-16	Liver
220296	-3.144641982	1.23E-06	Liver
33	-3.129721238	5.08E-11	Liver
1610	-3.1214284	1.98E-13	Liver
57127	-3.098674835	9.51E-07	Liver
64850	-3.082381662	9.32E-11	Liver
127733	-3.056905948	2.85E-16	Liver
10747	-3.051999937	1.27E-11	Liver
51733	-3.026910224	1.78E-12	Liver
647309	-3.021409414	9.85E-18	Liver
51181	-2.960905212	6.11E-31	Liver
10877	-2.954639343	9.71E-10	Liver
1491	-2.945482174	8.72E-17	Liver
284111	-2.942518037	3.81E-09	Liver
81494	-2.939122589	3.62E-08	Liver
319	-2.930581334	1.52E-09	Liver
387778	-2.919400616	2.67E-21	Liver

116519	-2.898018211	3.03E-10	Liver
5972	-2.885994898	1.74E-12	Liver
116085	-2.872523796	3.03E-06	Liver
84696	-2.872257106	7.37E-16	Liver
1579	-2.834763248	5.63E-10	Liver
92292	-2.833289658	2.90E-13	Liver
1586	-2.812924642	6.70E-07	Liver
54363	-2.807434021	2.80E-12	Liver
8529	-2.807286875	2.32E-11	Liver
1036	-2.805716256	2.51E-12	Liver
8856	-2.800534175	2.00E-09	Liver
6540	-2.775678847	2.92E-14	Liver
80168	-2.772388993	2.11E-10	Liver
2938	-2.759558182	2.69E-08	Liver
7069	-2.739495549	3.10E-05	Liver
64816	-2.737209244	9.18E-11	Liver
10157	-2.718668055	7.21E-20	Liver
9154	-2.711532723	4.82E-11	Liver
1549	-2.707803942	3.45E-05	Liver
6694	-2.706414153	1.04E-06	Liver
3080	-2.7037839	1.55E-09	Liver
367	-2.703242566	2.77E-11	Liver
2328	-2.702000145	2.52E-14	Liver
762	-2.697535612	3.49E-10	Liver
148738	-2.695622829	2.24E-09	Liver
6006	-2.688564096	7.02E-19	Liver
5244	-2.685746575	1.44E-14	Liver
23541	-2.68360633	2.25E-20	Liver
7364	-2.663168809	1.03E-11	Liver
1496	-2.66289679	7.63E-06	Liver
284541	-2.659305136	1.10E-08	Liver
6530	-2.656924831	1.41E-05	Liver
57451	-2.632697059	1.27E-06	Liver
11199	-2.628160785	1.10E-10	Liver
1571	-2.623599904	7.49E-05	Liver
731	-2.618063148	5.86E-12	Liver
8608	-2.617512493	7.93E-10	Liver
54658	-2.615560776	3.42E-09	Liver
641654	-2.610637328	4.51E-05	Liver
4311	-2.610186024	0.000131605	Liver
388503	-2.608669003	5.12E-08	Liver
219595	-2.600531801	1.89E-09	Liver
26998	-2.591576099	2.27E-07	Liver
2538	-2.590593832	1.28E-09	Liver
4547	-2.587322913	2.05E-12	Liver
29958	-2.587229912	6.52E-15	Liver
728655	-2.586577557	2.63E-11	Liver
283848	-2.57761004	1.77E-14	Liver
54550	-2.559805604	4.22E-08	Liver
53841	-2.559476042	4.42E-08	Liver
54579	-2.557555466	2.59E-11	Liver
3131	-2.551294301	1.41E-15	Liver
54959	-2.542773959	4.02E-06	Liver
1558	-2.540787841	4.41E-08	Liver
2161	-2.532192707	2.48E-10	Liver
5444	-2.519992109	1.92E-09	Liver
151126	-2.519926309	9.24E-11	Liver
763	-2.516179728	4.15E-09	Liver

5340	-2.514878637	4.44E-09	Liver
1559	-2.511470106	1.06E-07	Liver
8824	-2.501890717	7.13E-22	Liver
1370	-2.497514887	1.30E-09	Liver
5068	-2.49691335	0.002652805	Liver
2165	-2.493537473	8.36E-10	Liver
440925	-2.492460873	2.01E-16	Liver
114899	-2.485537586	1.76E-10	Liver
3950	-2.485133066	7.35E-06	Liver
563	-2.482747737	6.94E-13	Liver
64241	-2.474803506	1.30E-08	Liver
363	-2.46567524	9.54E-07	Liver
339221	-2.44963772	1.01E-09	Liver
2939	-2.44683724	5.10E-06	Liver
8630	-2.441956667	4.63E-10	Liver
92129	-2.441393697	8.02E-11	Liver
64902	-2.441027865	1.10E-07	Liver
118471	-2.43162113	1.22E-08	Liver
23460	-2.415707567	6.00E-15	Liver
55889	-2.410452265	8.11E-10	Liver
10814	-2.409981134	0.000505673	Liver
162282	-2.409093754	1.95E-06	Liver
389643	-2.407587	5.55E-10	Liver
6557	-2.401763868	2.10E-06	Liver
127665	-2.391596551	1.21E-09	Liver
221223	-2.390010346	1.12E-10	Liver
388646	-2.381345696	3.43E-07	Liver
55224	-2.364305948	6.56E-17	Liver
1807	-2.354865669	1.42E-08	Liver
570	-2.346764125	2.00E-12	Liver
9075	-2.340039467	5.10E-07	Liver
130	-2.338317039	8.40E-12	Liver
345	-2.333195064	2.48E-07	Liver
2158	-2.331612546	1.00E-05	Liver
197	-2.325331144	5.03E-07	Liver
162417	-2.325131189	1.35E-12	Liver
1962	-2.323391529	1.03E-19	Liver
4143	-2.320186884	1.16E-11	Liver
22977	-2.312538268	4.92E-08	Liver
51268	-2.31132983	3.84E-14	Liver
27329	-2.306032936	7.36E-10	Liver
1066	-2.301027878	7.98E-12	Liver
122664	-2.29802453	1.40E-14	Liver
7366	-2.296257708	1.47E-06	Liver
84675	-2.287236306	0.000307871	Liver
25987	-2.286965415	1.90E-19	Liver
126	-2.279066403	3.31E-06	Liver
154	-2.274168955	1.70E-16	Liver
55867	-2.272089745	0.000167034	Liver
3081	-2.26624001	5.44E-13	Liver
29948	-2.259594416	5.95E-15	Liver
3240	-2.258811865	6.08E-07	Liver
146894	-2.256042709	6.26E-09	Liver
3953	-2.253638447	3.88E-10	Liver
54600	-2.251172905	1.56E-08	Liver
10786	-2.250540674	4.79E-12	Liver
197257	-2.250087322	8.16E-16	Liver
5053	-2.243425913	1.44E-09	Liver

246778	-2.241453976	1.25E-13	Liver
1238	-2.238171405	1.46E-14	Liver
157855	-2.230074245	1.83E-06	Liver
6580	-2.227404149	7.28E-05	Liver
84217	-2.222596496	8.88E-18	Liver
23316	-2.220573371	1.08E-08	Liver
57733	-2.214980422	5.21E-06	Liver
2822	-2.209347194	9.56E-08	Liver
51666	-2.201048119	1.37E-11	Liver
23026	-2.197214542	2.16E-12	Liver
27121	-2.194164422	1.40E-06	Liver
154661	-2.193710434	1.31E-14	Liver
1555	-2.192176925	3.73E-07	Liver
116285	-2.192100287	2.35E-05	Liver
7060	-2.187133966	7.76E-08	Liver
375719	-2.178935676	3.21E-07	Liver
57834	-2.174179396	4.04E-11	Liver
3697	-2.174003666	5.21E-10	Liver
27232	-2.173416954	6.24E-07	Liver
648791	-2.171270991	1.89E-08	Liver
6258	-2.163952685	3.73E-06	Liver
85407	-2.163810215	1.94E-05	Liver
137872	-2.162931766	7.64E-16	Liver
149563	-2.157494434	9.14E-06	Liver
336	-2.152675615	1.06E-06	Liver
189	-2.152029181	9.00E-07	Liver
79611	-2.145644803	1.58E-09	Liver
10841	-2.143253878	2.13E-06	Liver
80129	-2.138862243	1.03E-10	Liver
59272	-2.138030333	6.06E-05	Liver
58510	-2.131863515	1.71E-07	Liver
13	-2.131511976	3.58E-09	Liver
3764	-2.12478936	2.93E-19	Liver
2875	-2.122879058	1.35E-10	Liver
7276	-2.122352287	1.85E-07	Liver
6581	-2.121381456	4.01E-10	Liver
3827	-2.120035931	4.75E-08	Liver
1101	-2.118663123	4.36E-12	Liver
342096	-2.117391994	5.10E-12	Liver
150280	-2.114735502	7.26E-11	Liver
1109	-2.108724642	9.41E-08	Liver
622	-2.10654437	2.09E-12	Liver
27284	-2.105894656	4.32E-08	Liver
1573	-2.103012798	1.87E-15	Liver
5950	-2.094007429	3.04E-11	Liver
2752	-2.082062863	2.27E-09	Liver
9429	-2.079806313	9.86E-13	Liver
340419	-2.077917133	8.51E-07	Liver
51716	-2.077792559	4.13E-09	Liver
84706	-2.070027339	2.37E-16	Liver
5106	-2.068264565	5.24E-19	Liver
10878	-2.065629461	3.20E-06	Liver
64405	-2.059352573	1.36E-06	Liver
254778	-2.059290702	1.66E-14	Liver
392636	-2.057006956	2.52E-13	Liver
55277	-2.055381548	2.00E-12	Liver
10	-2.054071496	5.45E-06	Liver
134526	-2.050367814	1.27E-06	Liver

252995	-2.047143952	3.10E-05	Liver
51557	-2.046706484	3.27E-07	Liver
53345	-2.042441181	2.07E-10	Liver
200931	-2.041928273	2.42E-06	Liver
23600	-2.041755088	7.10E-15	Liver
389434	-2.039938975	2.16E-07	Liver
1	-2.037315343	1.24E-07	Liver
2052	-2.036725134	5.35E-16	Liver
4329	-2.030627351	2.45E-14	Liver
5313	-2.028447711	5.87E-08	Liver
64240	-2.027899819	3.50E-08	Liver
2944	-2.022579213	0.009976166	Liver
56961	-2.018755223	1.12E-06	Liver
2329	-2.018321391	6.15E-20	Liver
3745	-2.017313337	1.61E-07	Liver
1733	-2.017145275	4.84E-07	Liver
201651	-2.015140559	6.44E-12	Liver
3700	-2.013340495	1.24E-09	Liver
7704	-2.012542869	6.06E-10	Liver
91614	-2.011845382	1.32E-16	Liver
23743	-2.010430491	9.45E-12	Liver
2925	-2.010034454	1.69E-06	Liver
266629	-2.008351975	2.98E-08	Liver
23305	-2.002759308	2.47E-07	Liver
6097	-2.001636386	1.61E-10	Liver
55244	-1.996380488	1.09E-13	Liver
60676	-1.991118703	1.00E-05	Liver
7274	-1.977836271	2.10E-08	Liver
6539	-1.976463123	6.79E-09	Liver
6514	-1.970301696	3.61E-08	Liver
340811	-1.960479265	1.38E-08	Liver
388595	-1.959534351	3.84E-06	Liver
1361	-1.959247243	6.34E-10	Liver
5166	-1.958949038	3.25E-11	Liver
173	-1.952954771	3.01E-05	Liver
3638	-1.952896923	1.10E-13	Liver
6569	-1.952070755	2.27E-12	Liver
10599	-1.950398159	7.91E-07	Liver
1553	-1.944128472	1.83E-05	Liver
8309	-1.941222829	9.18E-14	Liver
51302	-1.939494846	1.56E-05	Liver
8991	-1.938966392	2.52E-16	Liver
202151	-1.929594141	1.32E-06	Liver
2160	-1.928649315	1.99E-11	Liver
3484	-1.927330404	7.70E-06	Liver
28999	-1.925286041	6.40E-14	Liver
6652	-1.92264334	5.44E-17	Liver
6457	-1.92050361	1.20E-05	Liver
54578	-1.917073815	4.87E-07	Liver
728635	-1.916495197	4.60E-20	Liver
7447	-1.914414767	9.45E-07	Liver
18	-1.908346203	2.84E-13	Liver
350	-1.907182726	7.66E-07	Liver
10351	-1.903014725	1.18E-06	Liver
646023	-1.902525436	1.41E-09	Liver
64881	-1.900205517	7.30E-05	Liver
54490	-1.898788188	5.91E-09	Liver
80274	-1.898309878	0.000222245	Liver

729	-1.896362796	2.06E-06	Liver
9622	-1.896322474	1.42E-05	Liver
1565	-1.890011742	3.78E-06	Liver
722	-1.888673784	1.44E-06	Liver
23632	-1.884640639	3.38E-06	Liver
6926	-1.87481975	8.86E-09	Liver
6296	-1.871973227	1.11E-09	Liver
54923	-1.86978124	2.53E-13	Liver
148709	-1.869589911	2.03E-09	Liver
83758	-1.866094079	2.26E-10	Liver
316	-1.864434055	8.99E-06	Liver
90586	-1.85838723	1.92E-08	Liver
64849	-1.857816396	0.001296927	Liver
23464	-1.85559359	4.51E-18	Liver
1564	-1.852153003	1.42E-07	Liver
6568	-1.839991451	2.56E-07	Liver
8858	-1.837497009	4.03E-06	Liver
140689	-1.836001012	4.68E-05	Liver
3273	-1.832499561	0.000958586	Liver
4051	-1.830983601	4.46E-07	Liver
66002	-1.82152624	1.94E-08	Liver
23491	-1.818034161	3.23E-08	Liver
135138	-1.816682597	1.17E-07	Liver
90523	-1.813777371	5.17E-07	Liver
7108	-1.810645734	1.46E-14	Liver
11001	-1.806636779	3.80E-08	Liver
55937	-1.806194703	2.33E-08	Liver
124	-1.802360648	8.73E-06	Liver
23676	-1.798857273	1.58E-06	Liver
389058	-1.797011259	2.86E-07	Liver
229	-1.796237228	4.90E-05	Liver
7018	-1.792293533	1.34E-06	Liver
23562	-1.792262006	3.94E-12	Liver
130013	-1.785940588	6.69E-06	Liver
3053	-1.785492692	8.06E-07	Liver
5624	-1.78473722	6.64E-09	Liver
5267	-1.784639315	4.58E-06	Liver
51703	-1.784464978	9.63E-13	Liver
51085	-1.782296692	3.41E-12	Liver
285386	-1.777823238	5.81E-11	Liver
11162	-1.772397689	5.39E-20	Liver
1646	-1.771308178	9.84E-08	Liver
29947	-1.767001529	3.45E-05	Liver
23498	-1.762735941	2.91E-15	Liver
2147	-1.761040679	3.47E-06	Liver
88	-1.757534748	0.000829251	Liver
114	-1.756945859	3.02E-05	Liver
6529	-1.75669966	9.09E-08	Liver
100133985	-1.754435472	8.71E-14	Liver
400830	-1.75327077	2.04E-05	Liver
2155	-1.753155066	6.41E-07	Liver
4522	-1.75075608	1.01E-20	Liver
3699	-1.750631055	5.74E-07	Liver
1812	-1.743626276	3.56E-06	Liver
26049	-1.733556203	1.15E-10	Liver
25830	-1.731380571	0.001049806	Liver
9104	-1.730979983	1.70E-10	Liver
2674	-1.729619873	0.00045682	Liver

3485	-1.724353291	3.12E-07	Liver
641372	-1.718851177	4.42E-14	Liver
161247	-1.71410647	8.52E-11	Liver
145645	-1.713251504	1.30E-08	Liver
84141	-1.712743601	1.29E-10	Liver
3078	-1.712161657	1.92E-05	Liver
2277	-1.711940674	2.81E-06	Liver
1244	-1.710984812	4.73E-07	Liver
1409	-1.705954953	0.001772125	Liver
7036	-1.704998436	4.36E-08	Liver
725	-1.697858551	6.90E-09	Liver
732	-1.697479592	6.73E-06	Liver
64072	-1.695090889	3.60E-12	Liver
54979	-1.690972425	4.14E-05	Liver
134147	-1.688808704	6.02E-11	Liver
353322	-1.686001544	4.10E-12	Liver
85320	-1.675704955	3.22E-08	Liver
211	-1.674562291	1.87E-19	Liver
339398	-1.674551532	1.37E-06	Liver
57644	-1.671635731	1.82E-06	Liver
79632	-1.671606222	9.74E-11	Liver
404037	-1.670850274	1.53E-07	Liver
55256	-1.669461659	1.36E-23	Liver
144193	-1.667402859	1.08E-08	Liver
5827	-1.666928127	4.28E-19	Liver
100130015	-1.66654307	3.58E-16	Liver
5446	-1.665526034	6.50E-10	Liver
27443	-1.664700349	2.01E-07	Liver
3172	-1.659956779	3.12E-10	Liver
56666	-1.659168261	9.61E-07	Liver
9848	-1.652106812	5.33E-08	Liver
2153	-1.65189493	4.29E-10	Liver
5091	-1.651124383	1.24E-15	Liver
1577	-1.649961192	1.16E-06	Liver
5959	-1.649024901	7.85E-11	Liver
7363	-1.64304265	1.84E-06	Liver
11209	-1.642185264	2.04E-11	Liver
216	-1.639266577	3.16E-14	Liver
10991	-1.63555521	1.72E-07	Liver
1428	-1.633220079	6.13E-08	Liver
79750	-1.632703096	0.000382985	Liver
3990	-1.628274878	2.54E-08	Liver
387700	-1.626817788	5.97E-05	Liver
283	-1.626392771	4.64E-09	Liver
58503	-1.62485564	0.000536632	Liver
4485	-1.624810939	3.04E-09	Liver
10060	-1.623829654	4.93E-09	Liver
170961	-1.622605857	2.74E-15	Liver
162515	-1.618509998	2.06E-05	Liver
3373	-1.613255883	1.51E-13	Liver
23086	-1.609976559	3.50E-05	Liver
5243	-1.608435471	4.71E-06	Liver
2053	-1.607747976	6.58E-13	Liver
64799	-1.606010778	1.24E-07	Liver
1842	-1.603129811	2.97E-11	Liver
2099	-1.60304896	2.74E-05	Liver
79791	-1.602930075	4.34E-18	Liver
132158	-1.601271945	2.10E-15	Liver

3158	-1.597526971	1.68E-05	Liver
254295	-1.597438267	9.50E-05	Liver
55638	-1.593865937	8.90E-10	Liver
2646	-1.592265223	2.37E-09	Liver
54886	-1.590046247	1.07E-07	Liver
1757	-1.589530088	2.20E-11	Liver
148534	-1.58948901	5.43E-13	Liver
6997	-1.588420562	0.001026541	Liver
440184	-1.585018754	3.44E-08	Liver
3818	-1.584073711	1.75E-08	Liver
1583	-1.580616511	0.001032926	Liver
91703	-1.576486837	5.51E-08	Liver
347853	-1.576163297	7.47E-09	Liver
27141	-1.5755584	2.61E-12	Liver
441432	-1.571055464	4.99E-06	Liver
873	-1.56957615	3.92E-19	Liver
85474	-1.569527678	1.24E-10	Liver
155066	-1.569048785	1.70E-14	Liver
1528	-1.567619799	3.57E-17	Liver
2620	-1.563463883	3.21E-10	Liver
10267	-1.560464021	1.30E-07	Liver
392465	-1.560136531	3.88E-07	Liver
283600	-1.559635198	0.009374369	Liver
3263	-1.558205566	6.51E-05	Liver
246181	-1.555975307	2.78E-08	Liver
217	-1.555557783	3.26E-17	Liver
4610	-1.553884248	9.77E-13	Liver
341	-1.552446479	3.19E-11	Liver
6359	-1.55083983	1.55E-06	Liver
7498	-1.549006894	8.43E-06	Liver
6307	-1.537012412	3.71E-16	Liver
4489	-1.53597136	0.000150082	Liver
92960	-1.535387395	3.66E-17	Liver
1645	-1.533714091	1.67E-07	Liver
10246	-1.527935655	0.000185867	Liver
256394	-1.527539859	0.000336529	Liver
4153	-1.526803885	0.00105424	Liver
7923	-1.526687803	3.17E-18	Liver
91608	-1.524795076	0.002761338	Liver
7399	-1.521732807	9.70E-06	Liver
6783	-1.519703081	0.000662302	Liver
1557	-1.519338859	1.57E-05	Liver
338645	-1.515697095	0.000299315	Liver
5625	-1.514289804	0.000710285	Liver
5414	-1.510975744	1.90E-08	Liver
246182	-1.507954623	3.77E-14	Liver
3029	-1.507477208	5.87E-19	Liver
257629	-1.505628374	2.34E-07	Liver
646962	-1.502819722	2.30E-06	Liver
3795	-1.501460188	8.99E-10	Liver
6579	-1.500401889	0.000254107	Liver
197358	1.501108307	3.69E-17	Liver
7100	1.501914987	9.16E-13	Liver
10287	1.502068329	8.40E-24	Liver
9662	1.50245821	2.96E-20	Liver
387751	1.503394451	1.33E-10	Liver
9466	1.50381104	1.46E-17	Liver
3486	1.504043997	2.93E-12	Liver

9537	1.504218726	1.84E-24	Liver
285596	1.504420185	3.62E-09	Liver
56130	1.505638446	2.29E-07	Liver
79161	1.505696738	2.54E-14	Liver
3399	1.506303093	1.38E-18	Liver
5420	1.506591378	1.78E-18	Liver
80757	1.507610589	2.02E-07	Liver
8645	1.508400835	1.87E-05	Liver
221472	1.508512482	1.95E-13	Liver
199800	1.508815884	5.28E-14	Liver
25790	1.510626458	1.87E-08	Liver
3490	1.511773938	2.23E-17	Liver
9938	1.511926517	2.99E-21	Liver
3215	1.51204201	4.23E-13	Liver
84069	1.512913544	2.18E-06	Liver
51225	1.514265851	2.40E-22	Liver
5322	1.514714181	2.16E-10	Liver
157313	1.51494998	1.08E-07	Liver
3902	1.515868397	1.35E-08	Liver
113	1.51622468	2.03E-17	Liver
2036	1.516340548	5.13E-08	Liver
23531	1.516559911	1.45E-16	Liver
55351	1.517115961	7.75E-11	Liver
256380	1.517407989	1.60E-06	Liver
644165	1.51741954	3.58E-09	Liver
6415	1.517554525	1.15E-12	Liver
4542	1.518113156	1.32E-18	Liver
24147	1.518549536	1.41E-15	Liver
7305	1.518974297	6.99E-16	Liver
2793	1.519954194	6.33E-17	Liver
4242	1.520329694	9.29E-29	Liver
1058	1.520583595	6.76E-07	Liver
339665	1.520690997	1.27E-17	Liver
55630	1.5207047	0.000145917	Liver
283663	1.521150649	1.18E-12	Liver
8492	1.522339039	0.000331222	Liver
23129	1.522449319	1.17E-28	Liver
200879	1.522590016	8.25E-05	Liver
3910	1.522902594	9.97E-23	Liver
2296	1.523230266	6.08E-09	Liver
719	1.523662969	5.94E-14	Liver
3363	1.52378444	1.61E-11	Liver
55803	1.524375275	5.70E-22	Liver
64097	1.525198209	4.97E-13	Liver
85315	1.52566569	1.52E-08	Liver
3787	1.526023773	9.78E-07	Liver
257106	1.526420008	4.63E-17	Liver
701	1.527103914	4.18E-08	Liver
2274	1.527519727	1.73E-08	Liver
2358	1.527749967	9.76E-09	Liver
54575	1.528126126	0.001772915	Liver
55859	1.528485331	0.002653678	Liver
129293	1.528585325	0.000342066	Liver
6768	1.529278073	7.91E-07	Liver
5552	1.529959285	1.92E-14	Liver
63923	1.530035718	2.91E-07	Liver
2571	1.53012319	4.37E-05	Liver
166614	1.531029152	1.68E-14	Liver

113157	1.531770546	4.71E-10	Liver
85027	1.531909464	4.41E-12	Liver
728392	1.532498325	6.23E-12	Liver
5621	1.533188392	6.25E-11	Liver
2826	1.533201776	5.17E-10	Liver
1464	1.533311857	4.29E-18	Liver
308	1.533933875	1.25E-17	Liver
171024	1.534566414	4.52E-11	Liver
10381	1.535059388	1.08E-14	Liver
3274	1.535460605	7.24E-10	Liver
5916	1.535806518	1.61E-21	Liver
55190	1.53599255	2.27E-07	Liver
4162	1.536566191	4.43E-24	Liver
7621	1.536613462	1.41E-21	Liver
2207	1.536695113	9.92E-16	Liver
9510	1.536715489	3.67E-12	Liver
6857	1.536882712	0.001199508	Liver
2252	1.538745633	2.72E-08	Liver
146760	1.538929377	2.09E-07	Liver
55964	1.539279283	2.83E-06	Liver
1620	1.540242546	1.10E-06	Liver
29992	1.540488246	1.53E-16	Liver
254228	1.540899106	3.56E-16	Liver
100033820	1.541202041	5.03E-08	Liver
53829	1.541238405	2.53E-12	Liver
84002	1.541485672	8.77E-10	Liver
22856	1.54189704	4.97E-21	Liver
333929	1.541929627	1.75E-16	Liver
3112	1.542865768	6.57E-07	Liver
26576	1.54430731	3.75E-07	Liver
5523	1.5446648	3.76E-12	Liver
3589	1.545095896	4.94E-08	Liver
8643	1.545129299	1.98E-11	Liver
30846	1.545635497	1.79E-21	Liver
9034	1.545800887	1.28E-17	Liver
4613	1.54580539	0.000122795	Liver
23643	1.545980113	1.34E-06	Liver
164668	1.546079072	1.30E-10	Liver
84848	1.546155187	4.23E-13	Liver
135228	1.546429137	4.72E-07	Liver
374872	1.546573182	3.80E-17	Liver
80352	1.547014118	5.38E-10	Liver
152110	1.547016899	1.56E-07	Liver
130026	1.547598117	1.73E-18	Liver
84689	1.547621416	6.96E-12	Liver
445347	1.548216993	2.41E-10	Liver
3223	1.548590474	1.23E-05	Liver
9254	1.548626162	9.72E-11	Liver
157378	1.549168671	2.43E-20	Liver
83849	1.549179651	2.28E-08	Liver
90381	1.549291225	1.95E-08	Liver
1436	1.549497563	7.28E-15	Liver
130132	1.550521157	8.16E-11	Liver
150763	1.55208537	2.11E-06	Liver
10584	1.553558566	7.75E-05	Liver
9781	1.554563214	4.35E-15	Liver
3001	1.554847005	1.35E-09	Liver
342615	1.554878506	4.32E-11	Liver

4283	1.554939752	7.44E-06	Liver
57526	1.556034618	6.40E-09	Liver
5150	1.55656466	6.62E-26	Liver
192683	1.556775464	2.55E-10	Liver
10161	1.557051966	9.36E-27	Liver
58494	1.557354223	7.71E-10	Liver
80119	1.557954204	9.21E-10	Liver
25891	1.558591881	3.35E-09	Liver
80301	1.558727075	2.64E-30	Liver
4356	1.55956445	2.08E-07	Liver
10156	1.560010025	1.46E-16	Liver
23096	1.560439172	2.03E-15	Liver
862	1.561932431	1.47E-09	Liver
56105	1.56284929	8.36E-10	Liver
1852	1.563507579	8.01E-05	Liver
23551	1.563941928	1.30E-15	Liver
220108	1.564439391	1.05E-11	Liver
397	1.564671347	2.57E-27	Liver
55081	1.566396094	5.30E-12	Liver
9912	1.566480157	2.50E-07	Liver
4648	1.566540362	0.000118436	Liver
23171	1.566902361	6.85E-15	Liver
83700	1.567315584	1.19E-23	Liver
1734	1.568156479	0.000520745	Liver
93663	1.568296757	5.13E-25	Liver
221	1.56855612	3.59E-17	Liver
3749	1.568908373	1.53E-18	Liver
11065	1.56958777	1.62E-07	Liver
29951	1.56978789	2.81E-10	Liver
78989	1.570476844	2.87E-07	Liver
2657	1.570790652	2.39E-09	Liver
56125	1.571286909	9.58E-07	Liver
971	1.571450194	9.64E-12	Liver
284406	1.57182873	9.15E-10	Liver
3109	1.571965763	1.82E-13	Liver
1903	1.572854083	2.77E-11	Liver
23349	1.572864554	3.38E-10	Liver
4753	1.572880528	6.21E-05	Liver
356	1.573106803	2.39E-09	Liver
255057	1.573170679	4.19E-11	Liver
64174	1.573641872	1.28E-13	Liver
26228	1.574201596	1.53E-10	Liver
81035	1.574278143	1.33E-05	Liver
414332	1.574435377	1.39E-06	Liver
4861	1.575033693	4.16E-09	Liver
92912	1.575408863	3.75E-15	Liver
29128	1.575469476	3.51E-09	Liver
1230	1.576206533	2.27E-14	Liver
338761	1.576283088	1.08E-06	Liver
58516	1.57804741	2.29E-19	Liver
81831	1.578475876	1.14E-12	Liver
54660	1.578768788	4.87E-15	Liver
90246	1.579487787	9.33E-10	Liver
79729	1.579667704	1.30E-14	Liver
56905	1.579709424	7.66E-35	Liver
9497	1.580168579	2.49E-17	Liver
23218	1.580747472	4.16E-31	Liver
128346	1.580759507	1.04E-15	Liver

51471	1.580832073	4.02E-05	Liver
84518	1.580919359	2.50E-06	Liver
57717	1.581118142	2.25E-07	Liver
2004	1.581341159	3.62E-13	Liver
94015	1.581430083	3.21E-11	Liver
3122	1.581708628	4.29E-12	Liver
23284	1.58313045	1.53E-11	Liver
54328	1.583338503	6.49E-16	Liver
3113	1.584078873	6.18E-13	Liver
80115	1.58438749	1.55E-05	Liver
54518	1.584874858	2.17E-08	Liver
1240	1.585796953	2.57E-17	Liver
3653	1.585816807	1.71E-07	Liver
3945	1.586013196	1.15E-14	Liver
2212	1.586040195	4.60E-15	Liver
55586	1.586522561	2.78E-05	Liver
773	1.586595807	5.00E-11	Liver
10643	1.586754222	6.01E-05	Liver
90273	1.587473284	5.58E-10	Liver
7762	1.587994267	2.59E-06	Liver
393	1.588685195	3.65E-14	Liver
79626	1.588955115	1.08E-16	Liver
51286	1.589258432	1.02E-11	Liver
55824	1.590006729	1.11E-13	Liver
84159	1.590914099	8.73E-24	Liver
53616	1.591389979	1.00E-06	Liver
64231	1.591488967	6.79E-15	Liver
25816	1.592164361	7.26E-18	Liver
7010	1.592238662	4.29E-09	Liver
170959	1.592917499	5.79E-14	Liver
283948	1.593305966	1.81E-18	Liver
83552	1.593408002	3.89E-18	Liver
22806	1.593814715	2.95E-08	Liver
145820	1.593829702	3.68E-12	Liver
124739	1.593994383	1.12E-05	Liver
339453	1.594032802	1.05E-15	Liver
123099	1.594912518	4.94E-06	Liver
283358	1.595455073	3.03E-05	Liver
2949	1.596087423	1.41E-06	Liver
51393	1.596825929	1.52E-17	Liver
6538	1.597242982	0.002438434	Liver
89872	1.598045238	6.89E-06	Liver
147660	1.598416639	1.25E-08	Liver
137994	1.598948179	1.03E-13	Liver
148213	1.599377951	1.07E-06	Liver
9121	1.599665647	2.09E-15	Liver
441666	1.600945303	6.61E-06	Liver
56109	1.601165867	4.76E-10	Liver
317649	1.601317375	4.84E-11	Liver
22891	1.603437471	1.21E-10	Liver
5050	1.604288558	1.59E-10	Liver
64127	1.60442418	4.66E-11	Liver
53905	1.604558933	9.10E-08	Liver
165631	1.605159583	5.50E-09	Liver
5858	1.605838862	0.000454862	Liver
85004	1.608010123	1.28E-12	Liver
26249	1.609427057	5.79E-08	Liver
5337	1.609489197	1.18E-12	Liver

301	1.610200596	2.18E-16	Liver
146909	1.610870418	2.12E-08	Liver
9024	1.611077782	1.42E-05	Liver
6548	1.61188051	2.12E-28	Liver
54331	1.612307921	3.23E-23	Liver
57153	1.612338516	4.60E-23	Liver
3781	1.612641612	1.73E-06	Liver
284611	1.613049649	2.23E-21	Liver
493812	1.614084529	8.10E-21	Liver
29931	1.614282797	9.27E-16	Liver
8807	1.614994199	4.34E-11	Liver
57580	1.6150732	1.46E-22	Liver
160365	1.61593046	2.98E-12	Liver
148170	1.615953861	4.23E-18	Liver
56124	1.616001909	3.39E-10	Liver
10763	1.617063867	1.12E-22	Liver
10309	1.618397675	1.19E-05	Liver
3269	1.619258146	2.01E-17	Liver
344595	1.619996824	2.54E-13	Liver
349114	1.620080041	8.28E-18	Liver
56128	1.620134032	1.28E-06	Liver
168667	1.620443835	4.76E-07	Liver
9413	1.620969753	1.94E-12	Liver
10261	1.62161435	9.99E-14	Liver
27128	1.621649947	6.43E-19	Liver
64067	1.621919586	6.00E-12	Liver
64805	1.622729355	4.23E-11	Liver
51186	1.622768473	3.44E-10	Liver
1675	1.623272062	4.82E-12	Liver
57469	1.624093247	1.16E-13	Liver
51700	1.62441586	2.07E-07	Liver
143503	1.625172254	2.04E-13	Liver
9627	1.62583454	1.86E-08	Liver
7832	1.626134526	2.12E-18	Liver
80150	1.626206607	2.94E-10	Liver
3386	1.626287947	8.28E-08	Liver
3784	1.626827154	1.98E-12	Liver
57608	1.627247611	3.82E-17	Liver
6714	1.627256672	7.42E-11	Liver
146664	1.628158571	6.42E-10	Liver
10071	1.628685625	1.47E-07	Liver
57650	1.628696044	3.56E-10	Liver
79767	1.629663471	2.23E-07	Liver
56963	1.630781249	1.32E-11	Liver
84740	1.631894711	0.002759568	Liver
956	1.632741738	1.06E-07	Liver
404550	1.633092948	5.86E-10	Liver
1847	1.633242912	1.54E-12	Liver
100124692	1.633760093	8.26E-07	Liver
23090	1.636211588	1.12E-13	Liver
8516	1.636884002	2.21E-08	Liver
151056	1.636932494	6.47E-13	Liver
92949	1.637040635	1.02E-09	Liver
129080	1.637379582	6.72E-08	Liver
2262	1.637471481	4.67E-05	Liver
157983	1.637530355	2.43E-07	Liver
7136	1.637659867	3.22E-07	Liver
85376	1.638215897	2.34E-12	Liver

1608	1.639270795	2.59E-07	Liver
128344	1.639838021	2.09E-07	Liver
56648	1.639856901	8.37E-07	Liver
259197	1.640380191	8.24E-11	Liver
79168	1.640813433	1.21E-12	Liver
170958	1.641679599	2.42E-08	Liver
286122	1.641687385	1.82E-10	Liver
50846	1.642024116	1.88E-11	Liver
10234	1.643079993	2.47E-14	Liver
388242	1.64309687	7.55E-12	Liver
861	1.643216199	6.15E-18	Liver
3212	1.643221058	3.11E-24	Liver
92558	1.643307541	4.28E-08	Liver
51704	1.64339167	2.86E-12	Liver
125893	1.643740739	2.52E-10	Liver
51066	1.64381657	0.00050007	Liver
9294	1.644612407	1.68E-13	Liver
8676	1.645245367	1.62E-13	Liver
909	1.645458108	3.06E-10	Liver
1138	1.645535794	4.04E-09	Liver
11015	1.645956324	2.76E-10	Liver
79668	1.646038879	2.96E-12	Liver
57172	1.646766898	6.24E-10	Liver
11096	1.647240431	1.74E-15	Liver
388372	1.647598116	4.88E-10	Liver
64411	1.647910402	3.30E-25	Liver
55698	1.648152758	4.19E-13	Liver
8828	1.648819723	1.37E-17	Liver
155185	1.649541103	1.06E-06	Liver
57710	1.649681913	1.22E-12	Liver
7594	1.650795299	6.78E-10	Liver
8642	1.650803086	1.65E-27	Liver
9672	1.650872111	4.58E-25	Liver
55	1.651183003	3.78E-08	Liver
140710	1.65189864	2.00E-19	Liver
27253	1.652317892	1.59E-16	Liver
10562	1.652666696	5.61E-05	Liver
644	1.652962261	2.07E-14	Liver
6332	1.653280761	8.91E-08	Liver
57162	1.653355939	1.11E-27	Liver
3098	1.653719285	1.87E-15	Liver
84766	1.654676513	9.08E-11	Liver
8651	1.655144923	4.28E-12	Liver
9355	1.656042486	3.61E-08	Liver
65012	1.656079359	3.81E-11	Liver
120425	1.656660648	1.04E-14	Liver
1297	1.656967656	2.15E-09	Liver
10388	1.656975847	2.10E-09	Liver
115761	1.657031351	3.36E-14	Liver
56144	1.657552527	6.97E-10	Liver
4481	1.657609671	2.54E-15	Liver
3339	1.65763067	4.56E-23	Liver
1308	1.657731692	1.40E-09	Liver
53342	1.657945593	2.11E-05	Liver
29944	1.658656193	0.000173939	Liver
56992	1.659092007	9.86E-07	Liver
2069	1.660057247	4.58E-05	Liver
2702	1.660248764	4.87E-12	Liver

29967	1.660765582	2.89E-11	Liver
79949	1.66123616	2.75E-05	Liver
723809	1.661538706	1.79E-06	Liver
54504	1.661929702	2.29E-11	Liver
23052	1.661986382	1.46E-16	Liver
51765	1.663536316	8.14E-08	Liver
55653	1.665593457	2.30E-26	Liver
395	1.665638153	1.76E-18	Liver
375323	1.665703462	3.01E-05	Liver
51200	1.666489019	9.24E-08	Liver
56131	1.666757761	1.48E-11	Liver
57449	1.666801861	1.21E-27	Liver
1014	1.668409661	0.000442806	Liver
79608	1.668426398	4.23E-07	Liver
5272	1.669083567	6.86E-19	Liver
25902	1.67036213	1.21E-17	Liver
25789	1.670389994	6.27E-14	Liver
3216	1.670793516	8.14E-12	Liver
83729	1.671326048	1.78E-06	Liver
3128	1.671811019	8.37E-09	Liver
6769	1.671834701	3.54E-07	Liver
5317	1.672127187	1.27E-08	Liver
11025	1.672360996	1.35E-15	Liver
3115	1.673733399	2.57E-15	Liver
441204	1.674314452	1.21E-08	Liver
8497	1.674399415	6.03E-07	Liver
4803	1.67604384	2.13E-14	Liver
27147	1.676949514	4.65E-21	Liver
55769	1.677355449	4.24E-09	Liver
342926	1.678472599	2.84E-11	Liver
57333	1.678866127	7.21E-27	Liver
23114	1.67941687	5.14E-12	Liver
57655	1.679583626	3.24E-17	Liver
9639	1.68075954	8.88E-17	Liver
143903	1.680885852	1.51E-15	Liver
2740	1.681597964	3.77E-07	Liver
6039	1.681767518	5.27E-17	Liver
8076	1.682778806	2.65E-08	Liver
133022	1.682844525	2.84E-06	Liver
3199	1.68386228	3.47E-12	Liver
139716	1.683991602	8.53E-23	Liver
9427	1.684280769	0.000304132	Liver
3101	1.685620205	8.62E-10	Liver
55106	1.686430617	3.23E-12	Liver
4066	1.687010225	4.64E-26	Liver
3767	1.687307722	2.17E-07	Liver
100129842	1.687381754	4.99E-11	Liver
80863	1.688268539	5.67E-18	Liver
284677	1.688412653	2.91E-10	Liver
23507	1.688755722	7.60E-16	Liver
167691	1.689022043	4.74E-16	Liver
23363	1.689269922	6.89E-07	Liver
2882	1.689503291	1.70E-09	Liver
65108	1.690021047	2.53E-17	Liver
7031	1.690371656	2.32E-05	Liver
131405	1.691197362	1.48E-07	Liver
140628	1.691907911	2.00E-05	Liver
4017	1.692511655	1.01E-25	Liver

660	1.693826592	1.42E-10	Liver
624	1.694125398	4.45E-10	Liver
162681	1.694431149	1.36E-18	Liver
10316	1.694615832	1.09E-13	Liver
7940	1.69485968	1.09E-15	Liver
643224	1.695044668	3.53E-05	Liver
284340	1.696422614	7.08E-06	Liver
283209	1.697929939	6.04E-24	Liver
4224	1.698259289	0.003272967	Liver
9638	1.698540146	2.67E-15	Liver
116832	1.699688738	3.87E-05	Liver
84329	1.700089689	8.17E-22	Liver
219902	1.701685899	1.39E-14	Liver
80328	1.701880486	5.51E-08	Liver
51285	1.702472328	4.73E-22	Liver
728936	1.703590981	9.69E-07	Liver
347	1.704399593	4.27E-08	Liver
113026	1.704510929	6.21E-17	Liver
79630	1.704920221	4.08E-21	Liver
93166	1.704924797	9.32E-13	Liver
3206	1.706709359	0.000781309	Liver
7546	1.706936749	0.000140365	Liver
4689	1.707171247	1.09E-16	Liver
54619	1.707763396	5.02E-12	Liver
1359	1.708167333	3.59E-05	Liver
7710	1.708466857	1.95E-16	Liver
131450	1.708758675	3.56E-14	Liver
153733	1.708791043	4.65E-12	Liver
1521	1.709795197	3.45E-09	Liver
2825	1.710298084	4.82E-09	Liver
22998	1.710466147	1.10E-11	Liver
6352	1.710885544	9.26E-11	Liver
27303	1.711113865	1.99E-15	Liver
84671	1.71131839	9.35E-11	Liver
57636	1.713025983	2.31E-21	Liver
1602	1.71310218	1.05E-13	Liver
388115	1.713899921	2.09E-12	Liver
353219	1.714509982	1.26E-07	Liver
9398	1.71513201	5.83E-19	Liver
4056	1.715397142	2.62E-12	Liver
9493	1.71593884	1.21E-09	Liver
57692	1.716498418	2.79E-12	Liver
2824	1.717284821	2.02E-14	Liver
59352	1.717789151	1.78E-05	Liver
84258	1.717926086	1.29E-05	Liver
30851	1.717959803	1.09E-26	Liver
57493	1.718959738	7.62E-27	Liver
56853	1.719059099	3.15E-12	Liver
4987	1.719377818	1.95E-17	Liver
89858	1.719712182	3.89E-08	Liver
54437	1.719714411	1.02E-17	Liver
3202	1.72042236	2.99E-10	Liver
3684	1.720802215	1.31E-11	Liver
285973	1.721050736	3.94E-15	Liver
29125	1.721623163	1.96E-12	Liver
54843	1.721722793	1.20E-18	Liver
7903	1.721798678	2.45E-21	Liver
7462	1.72322834	1.56E-19	Liver

7124	1.723368469	2.09E-12	Liver
100133545	1.723812279	1.33E-05	Liver
4131	1.723928169	4.66E-19	Liver
1282	1.724102074	1.45E-21	Liver
10220	1.724909841	5.91E-15	Liver
4851	1.725282301	9.63E-35	Liver
4935	1.725492938	8.48E-06	Liver
57105	1.72593078	8.61E-11	Liver
23547	1.725971578	1.46E-11	Liver
11314	1.726357308	1.39E-18	Liver
4166	1.726452384	2.11E-10	Liver
7056	1.726954353	8.93E-18	Liver
6928	1.727663951	4.38E-07	Liver
84632	1.727989354	4.56E-21	Liver
2596	1.72894372	1.88E-10	Liver
2679	1.729385788	1.90E-07	Liver
84626	1.729573564	1.79E-13	Liver
3714	1.729671302	1.21E-17	Liver
9435	1.729979845	2.57E-24	Liver
11213	1.73071862	4.35E-15	Liver
6909	1.730858809	3.39E-20	Liver
10871	1.73096151	2.57E-14	Liver
388125	1.731422735	6.81E-10	Liver
2256	1.732363521	1.13E-14	Liver
139728	1.732641988	0.000114371	Liver
84417	1.732938137	4.03E-09	Liver
6678	1.733547097	6.66E-26	Liver
401612	1.734627719	1.20E-14	Liver
9985	1.735466623	8.43E-14	Liver
84561	1.735826514	5.40E-09	Liver
256714	1.735951581	0.000110947	Liver
8698	1.73688821	5.15E-15	Liver
6664	1.737906343	3.67E-10	Liver
57494	1.738511233	2.65E-17	Liver
781	1.738759282	3.89E-12	Liver
261734	1.738826312	2.32E-08	Liver
3090	1.739414734	2.21E-22	Liver
1636	1.739547556	1.61E-15	Liver
256691	1.740284059	6.52E-09	Liver
91227	1.740321198	2.56E-06	Liver
653	1.740631408	1.84E-09	Liver
389792	1.741377358	6.00E-14	Liver
64857	1.742247745	3.72E-32	Liver
3004	1.742345666	9.57E-12	Liver
8320	1.742485956	8.12E-10	Liver
51146	1.743629033	3.67E-09	Liver
89765	1.744495289	1.13E-08	Liver
57555	1.745101913	2.20E-14	Liver
161582	1.746844399	3.74E-11	Liver
51177	1.746857391	6.23E-24	Liver
158158	1.74703153	4.34E-05	Liver
1410	1.747294812	4.97E-07	Liver
286827	1.747734011	3.05E-16	Liver
122060	1.748682873	1.35E-05	Liver
3217	1.749247497	1.35E-09	Liver
57707	1.749866731	1.25E-11	Liver
9610	1.75050967	2.94E-18	Liver
254428	1.750632894	2.67E-15	Liver

170954	1.751126245	8.14E-29	Liver
64407	1.751275553	2.07E-13	Liver
26468	1.751282927	5.36E-12	Liver
8821	1.751724301	2.44E-12	Liver
140	1.752205099	1.89E-14	Liver
92922	1.753228019	7.47E-19	Liver
392255	1.753871511	7.35E-11	Liver
9806	1.753996011	2.68E-18	Liver
23466	1.75431265	4.86E-11	Liver
124930	1.754486307	1.55E-14	Liver
26150	1.755088022	2.18E-07	Liver
7041	1.755920678	2.80E-25	Liver
6755	1.755979824	5.39E-07	Liver
2139	1.756708593	1.17E-07	Liver
3248	1.757064397	0.004158308	Liver
2056	1.757335214	0.000206822	Liver
7351	1.758017849	2.71E-19	Liver
3560	1.758631261	1.28E-14	Liver
79412	1.758986293	6.55E-10	Liver
158931	1.759958613	8.12E-18	Liver
55808	1.760575683	8.24E-11	Liver
27111	1.761707474	4.70E-08	Liver
10462	1.761855936	6.83E-12	Liver
84842	1.762283136	1.17E-09	Liver
9001	1.762857908	1.26E-10	Liver
79901	1.763072193	1.00E-12	Liver
83714	1.763471866	2.21E-20	Liver
9424	1.763647577	1.52E-22	Liver
134	1.76523871	8.93E-06	Liver
286204	1.765984798	7.38E-14	Liver
608	1.766032596	2.24E-09	Liver
64943	1.766557289	1.32E-11	Liver
55118	1.766633824	8.62E-09	Liver
79788	1.766795378	4.81E-11	Liver
639	1.766888365	7.97E-21	Liver
7177	1.766911921	3.41E-05	Liver
64926	1.766996891	1.95E-21	Liver
6515	1.767106185	2.97E-15	Liver
51176	1.767152383	2.87E-06	Liver
79781	1.767787045	3.07E-11	Liver
11173	1.768409404	6.52E-28	Liver
11107	1.769019178	1.83E-10	Liver
247	1.770501686	2.08E-05	Liver
84247	1.770567869	4.01E-16	Liver
257194	1.77111525	4.49E-12	Liver
3059	1.771817213	1.45E-23	Liver
7188	1.772107763	2.44E-22	Liver
161742	1.772327908	3.91E-27	Liver
55214	1.772481821	5.78E-12	Liver
131368	1.774009422	1.79E-05	Liver
654346	1.774871583	1.43E-09	Liver
55576	1.77495769	1.59E-06	Liver
137835	1.776102869	1.75E-18	Liver
3623	1.77749298	8.59E-06	Liver
23194	1.778304382	1.87E-17	Liver
5101	1.778548834	3.58E-08	Liver
970	1.779266541	5.95E-11	Liver
9473	1.780120719	2.72E-19	Liver

253190	1.780706933	8.27E-08	Liver
4921	1.780773347	2.47E-10	Liver
482	1.780812384	6.74E-12	Liver
3119	1.780966715	3.18E-12	Liver
8792	1.78256254	9.65E-10	Liver
871	1.783560964	8.31E-30	Liver
10083	1.784385946	0.004900031	Liver
29798	1.784516225	6.54E-16	Liver
27036	1.784959579	1.79E-16	Liver
135398	1.785181923	1.29E-06	Liver
656	1.785702493	9.38E-07	Liver
23428	1.786796153	1.06E-15	Liver
7032	1.786862508	1.20E-05	Liver
50856	1.787001328	2.72E-22	Liver
3225	1.787683938	3.25E-06	Liver
10227	1.788263268	1.28E-30	Liver
4093	1.788851483	8.99E-10	Liver
7439	1.789400404	2.22E-16	Liver
55076	1.789868637	9.65E-06	Liver
112616	1.790434896	2.11E-22	Liver
2615	1.79048713	1.67E-20	Liver
5837	1.791263786	2.05E-15	Liver
27143	1.79128458	5.81E-27	Liver
1953	1.792452876	1.00E-07	Liver
10537	1.792528843	2.60E-07	Liver
5066	1.793469272	1.39E-18	Liver
3768	1.793670995	5.79E-15	Liver
3689	1.794006449	3.35E-18	Liver
89796	1.794267175	6.61E-27	Liver
55026	1.794633718	2.65E-09	Liver
115352	1.795440282	1.36E-08	Liver
9468	1.79664626	1.44E-10	Liver
3586	1.797571429	9.27E-14	Liver
23533	1.798136542	1.33E-22	Liver
9899	1.798210001	1.92E-06	Liver
79153	1.798344642	3.05E-09	Liver
339983	1.79844069	8.44E-08	Liver
1759	1.798667608	7.31E-09	Liver
57685	1.799012625	6.22E-12	Liver
340554	1.799309439	7.60E-10	Liver
63940	1.799411075	1.58E-25	Liver
9002	1.800157838	6.78E-10	Liver
5745	1.800339482	5.43E-11	Liver
10077	1.800379611	7.16E-13	Liver
606724	1.800635341	3.13E-14	Liver
4496	1.800649218	0.000538366	Liver
7117	1.800736563	1.72E-30	Liver
26153	1.801181164	3.02E-14	Liver
5314	1.801508096	8.61E-05	Liver
23529	1.802475967	4.37E-14	Liver
57552	1.802707909	8.32E-16	Liver
84807	1.803970043	1.19E-16	Liver
91156	1.804049763	6.71E-07	Liver
3428	1.804901934	6.94E-19	Liver
2170	1.80571405	3.89E-10	Liver
92241	1.80627357	7.13E-25	Liver
4065	1.807481771	1.12E-13	Liver
283431	1.80767714	5.70E-09	Liver

126567	1.807829446	8.10E-13	Liver
59339	1.808250303	4.91E-26	Liver
80312	1.808567479	1.80E-11	Liver
199720	1.809114026	3.96E-19	Liver
9260	1.809725183	9.95E-31	Liver
8029	1.810085727	9.49E-22	Liver
55771	1.810963557	1.10E-07	Liver
389336	1.811512186	1.18E-07	Liver
4680	1.812292946	1.27E-05	Liver
151313	1.812525369	3.87E-06	Liver
9456	1.812819383	8.18E-08	Liver
1489	1.813475506	7.20E-11	Liver
56127	1.81438311	4.53E-10	Liver
162979	1.814721672	2.42E-12	Liver
116211	1.815293131	1.04E-07	Liver
11237	1.816661905	2.78E-21	Liver
284023	1.816731523	6.22E-17	Liver
23254	1.818280151	1.42E-14	Liver
2784	1.818523466	2.47E-18	Liver
221416	1.818869392	0.000390303	Liver
80731	1.819254342	1.38E-10	Liver
170689	1.819403227	9.18E-11	Liver
3766	1.820077144	5.71E-08	Liver
57126	1.820393222	1.12E-08	Liver
11010	1.820460003	1.40E-14	Liver
81618	1.820794898	8.28E-15	Liver
10451	1.821701504	2.70E-14	Liver
83643	1.821953537	1.32E-14	Liver
255743	1.82255647	7.24E-09	Liver
8854	1.822872259	1.57E-08	Liver
84058	1.822952604	2.78E-13	Liver
84900	1.823573853	2.34E-11	Liver
7639	1.824362326	6.36E-12	Liver
9402	1.825195001	1.08E-09	Liver
6546	1.825303425	3.55E-19	Liver
7980	1.825834122	4.20E-09	Liver
27439	1.826120159	4.77E-19	Liver
152789	1.826150426	5.17E-10	Liver
342897	1.826331969	9.00E-12	Liver
221981	1.826594391	1.61E-12	Liver
79465	1.827404926	1.49E-12	Liver
5334	1.828050396	1.62E-23	Liver
7431	1.82946694	5.30E-32	Liver
4900	1.829790112	1.07E-21	Liver
150372	1.829941314	7.62E-19	Liver
7846	1.83017645	3.31E-31	Liver
219855	1.831443033	2.50E-22	Liver
55558	1.832283765	1.25E-14	Liver
30845	1.833106769	3.34E-19	Liver
92126	1.834024997	7.99E-10	Liver
944	1.834510361	3.24E-13	Liver
7169	1.834639839	8.03E-18	Liver
51411	1.834892285	4.08E-20	Liver
266747	1.835182817	1.69E-17	Liver
26253	1.836208241	2.14E-08	Liver
5159	1.836839469	6.23E-26	Liver
84188	1.83691052	1.38E-23	Liver
83872	1.8378954	3.01E-10	Liver

117854	1.838714299	8.83E-10	Liver
27065	1.840188579	7.26E-14	Liver
5017	1.840801318	2.97E-06	Liver
83648	1.841979878	1.91E-06	Liver
23111	1.842138147	8.70E-15	Liver
147700	1.842470673	5.78E-11	Liver
6519	1.842608068	0.000144183	Liver
898	1.843070331	7.84E-12	Liver
57611	1.843437919	3.60E-13	Liver
5217	1.8436293	3.54E-10	Liver
244	1.843977682	1.17E-05	Liver
1911	1.844121578	3.39E-24	Liver
84174	1.844229794	9.90E-15	Liver
90355	1.844235139	6.93E-10	Liver
55016	1.844310133	1.10E-16	Liver
126014	1.845186444	2.36E-18	Liver
27090	1.845400693	2.53E-20	Liver
29108	1.845530089	2.69E-14	Liver
4684	1.846622325	1.17E-06	Liver
2525	1.846710355	3.75E-06	Liver
7461	1.847560569	2.77E-13	Liver
4772	1.849796659	3.63E-18	Liver
152330	1.849942188	4.75E-11	Liver
3635	1.850055843	8.86E-27	Liver
430	1.85012425	1.33E-10	Liver
285195	1.850267572	2.39E-22	Liver
90113	1.850431771	5.88E-10	Liver
25925	1.851058124	4.05E-14	Liver
151888	1.85179006	5.05E-11	Liver
577	1.852528435	3.68E-13	Liver
623	1.852574269	1.22E-09	Liver
51214	1.852991769	8.49E-05	Liver
4082	1.853123306	9.32E-27	Liver
6688	1.853267339	4.58E-23	Liver
5361	1.854897376	1.23E-27	Liver
91607	1.856044856	2.08E-20	Liver
3815	1.856281538	2.25E-13	Liver
8609	1.856363773	1.29E-12	Liver
2841	1.856365412	4.53E-14	Liver
6916	1.857577838	4.13E-21	Liver
56849	1.857598485	8.69E-20	Liver
8633	1.857651458	1.41E-14	Liver
5087	1.857688119	4.29E-10	Liver
219738	1.857979851	3.93E-08	Liver
11098	1.859744457	3.31E-20	Liver
79444	1.859776217	2.01E-08	Liver
64420	1.859913835	9.87E-22	Liver
8654	1.860089561	3.36E-20	Liver
645121	1.862278556	4.31E-13	Liver
54842	1.862727884	2.78E-22	Liver
3741	1.863075988	8.77E-12	Liver
2268	1.86540874	1.21E-21	Liver
8826	1.865766601	1.68E-31	Liver
84752	1.86637992	5.43E-20	Liver
154075	1.867211189	1.42E-13	Liver
57168	1.867517827	4.40E-21	Liver
114793	1.867651216	2.27E-14	Liver
2124	1.868007029	1.34E-17	Liver

112464	1.86869536	1.85E-18	Liver
344787	1.869275658	6.89E-14	Liver
84814	1.869402203	8.32E-15	Liver
55529	1.870171086	1.69E-12	Liver
27239	1.87041339	2.58E-13	Liver
139065	1.870447832	1.74E-12	Liver
22846	1.87178352	4.16E-31	Liver
26031	1.871895562	1.33E-21	Liver
5341	1.872135326	3.53E-16	Liver
90865	1.872402463	2.98E-13	Liver
399669	1.872632284	1.25E-11	Liver
56121	1.872773615	2.95E-15	Liver
113278	1.872835181	6.70E-08	Liver
90249	1.873405999	2.92E-10	Liver
58499	1.874670633	2.44E-11	Liver
55604	1.874852528	2.89E-11	Liver
727936	1.87615068	2.41E-09	Liver
3673	1.876443366	5.77E-10	Liver
388662	1.877359119	3.97E-15	Liver
346157	1.878870545	7.25E-11	Liver
6710	1.879474306	5.32E-22	Liver
2983	1.880234264	9.80E-23	Liver
5355	1.880567235	5.10E-16	Liver
2769	1.88133594	1.09E-20	Liver
114569	1.881357284	5.51E-09	Liver
84254	1.881759576	5.18E-24	Liver
151963	1.882166066	1.75E-20	Liver
3687	1.882184802	6.63E-20	Liver
28232	1.882240153	2.72E-21	Liver
139818	1.882478766	9.34E-15	Liver
4064	1.882717995	2.69E-16	Liver
55509	1.883124081	8.52E-19	Liver
388228	1.883329885	2.23E-10	Liver
2322	1.883686379	8.79E-11	Liver
8325	1.883758656	4.78E-15	Liver
51450	1.884286922	5.24E-10	Liver
100132948	1.8851656	1.50E-10	Liver
81553	1.88533192	2.38E-16	Liver
85479	1.885579273	1.63E-11	Liver
399948	1.886125474	3.14E-10	Liver
10000	1.886259581	7.76E-18	Liver
124872	1.886449731	1.03E-05	Liver
654816	1.886647918	5.84E-15	Liver
84941	1.886840657	2.92E-12	Liver
7138	1.887317542	1.31E-06	Liver
283897	1.887463646	3.33E-14	Liver
56129	1.88761425	4.16E-12	Liver
8705	1.888134477	1.74E-16	Liver
388963	1.88847097	8.40E-24	Liver
169611	1.88855941	3.85E-21	Liver
126017	1.888964434	2.65E-08	Liver
91010	1.889814739	1.18E-40	Liver
10846	1.890958279	4.41E-16	Liver
5579	1.891320783	1.59E-14	Liver
79905	1.891481306	3.65E-14	Liver
145741	1.891682447	3.16E-05	Liver
84830	1.89251246	3.14E-07	Liver
284021	1.892758028	8.83E-16	Liver

10742	1.89314017	3.71E-17	Liver
6553	1.893212663	5.50E-21	Liver
644150	1.89335219	1.02E-08	Liver
51063	1.893389186	5.46E-34	Liver
57631	1.893821533	3.15E-16	Liver
8477	1.89410876	1.45E-20	Liver
114821	1.894369048	2.71E-07	Liver
157773	1.894491032	3.75E-17	Liver
54900	1.894513909	1.53E-13	Liver
54933	1.895103084	6.96E-14	Liver
1306	1.89522321	1.58E-14	Liver
9383	1.895816355	0.002352319	Liver
54470	1.897592082	2.09E-14	Liver
3034	1.89769336	0.001724276	Liver
29109	1.898248548	1.78E-33	Liver
976	1.898571601	4.11E-20	Liver
7223	1.898721185	6.56E-13	Liver
124961	1.899402263	8.97E-14	Liver
6624	1.89951872	3.73E-27	Liver
219623	1.899677628	1.19E-19	Liver
222962	1.900671882	8.36E-06	Liver
4345	1.901279987	4.65E-22	Liver
11123	1.901516732	5.87E-15	Liver
388692	1.901690881	1.50E-18	Liver
387763	1.901883913	8.81E-15	Liver
2246	1.901961707	6.34E-11	Liver
79772	1.90206288	3.01E-09	Liver
200373	1.90297233	3.54E-06	Liver
1284	1.903689668	3.03E-25	Liver
162967	1.903787468	3.77E-10	Liver
113828	1.90402875	7.51E-07	Liver
22859	1.904949279	4.77E-12	Liver
101	1.905905013	1.15E-21	Liver
79930	1.906401893	2.51E-28	Liver
79839	1.906953609	5.46E-26	Liver
641	1.907479482	1.55E-12	Liver
57460	1.907884942	1.56E-10	Liver
147372	1.908244672	6.65E-09	Liver
4240	1.908335528	1.26E-24	Liver
2678	1.908872758	3.18E-07	Liver
55800	1.909097512	9.38E-15	Liver
284656	1.909320482	4.44E-07	Liver
9289	1.909675038	1.51E-18	Liver
9290	1.909866267	6.90E-18	Liver
58529	1.910687162	1.20E-14	Liver
85012	1.912025099	9.65E-22	Liver
143458	1.912094419	2.94E-17	Liver
10586	1.912163566	2.51E-06	Liver
2984	1.912240655	0.000184088	Liver
7941	1.912596518	7.03E-13	Liver
9452	1.913468172	8.21E-13	Liver
203286	1.914638537	5.19E-08	Liver
5790	1.914783245	4.14E-17	Liver
83988	1.915335456	1.68E-14	Liver
1601	1.915453502	8.72E-19	Liver
150771	1.915816378	1.15E-16	Liver
55607	1.915825721	1.02E-08	Liver
54913	1.916694473	1.06E-18	Liver

54839	1.917946422	1.38E-15	Liver
78991	1.918283048	1.24E-25	Liver
8745	1.918416974	4.47E-06	Liver
2015	1.91864637	1.02E-10	Liver
51309	1.919099405	8.77E-22	Liver
374654	1.921724675	5.17E-16	Liver
51512	1.92179198	3.30E-12	Liver
56895	1.922219363	3.38E-13	Liver
22898	1.922385293	1.30E-30	Liver
375298	1.922446234	3.02E-21	Liver
3055	1.923031029	2.98E-21	Liver
6404	1.923993214	6.76E-23	Liver
9240	1.924533739	8.50E-37	Liver
284307	1.92681314	1.63E-12	Liver
5330	1.92691708	1.72E-22	Liver
283212	1.927538347	2.33E-13	Liver
1687	1.928018264	3.10E-10	Liver
4046	1.928159336	4.66E-18	Liver
114836	1.928834111	4.46E-13	Liver
149837	1.929016795	2.02E-11	Liver
1796	1.930009231	3.25E-34	Liver
256472	1.930049248	2.55E-07	Liver
9450	1.933144569	1.33E-20	Liver
942	1.933221728	1.20E-19	Liver
50650	1.93332022	1.89E-18	Liver
84302	1.933565635	2.61E-07	Liver
6503	1.934301446	4.93E-19	Liver
4916	1.934640044	1.80E-13	Liver
4323	1.934676024	2.29E-24	Liver
4312	1.935033012	6.39E-07	Liver
10186	1.935062687	5.03E-24	Liver
9498	1.935219687	7.05E-21	Liver
30818	1.935328464	8.84E-08	Liver
2162	1.936694682	1.11E-09	Liver
57198	1.937128478	8.82E-18	Liver
654463	1.937151689	5.07E-06	Liver
84441	1.937202214	4.38E-14	Liver
23136	1.937310288	7.89E-16	Liver
9214	1.938637578	6.35E-17	Liver
127294	1.938648452	1.43E-15	Liver
8463	1.939850661	8.16E-20	Liver
283229	1.940113948	1.62E-13	Liver
10184	1.940564169	2.08E-19	Liver
254531	1.941194455	8.78E-25	Liver
6876	1.941246915	2.33E-19	Liver
10052	1.941655142	2.88E-25	Liver
9839	1.942846964	1.90E-24	Liver
51564	1.942971551	1.48E-40	Liver
136	1.943199301	4.27E-11	Liver
3311	1.944968998	2.69E-10	Liver
6273	1.945761055	2.37E-08	Liver
440712	1.945932048	6.99E-08	Liver
7837	1.946643933	1.68E-24	Liver
146227	1.946912758	5.16E-12	Liver
340061	1.948236199	1.72E-26	Liver
58495	1.948802634	1.41E-09	Liver
4052	1.949318038	6.98E-15	Liver
23101	1.949398812	1.14E-08	Liver

149628	1.950049679	1.17E-11	Liver
124460	1.950085988	1.78E-15	Liver
23120	1.950448988	4.71E-07	Liver
4015	1.951293551	9.04E-12	Liver
3792	1.952654359	5.46E-06	Liver
85460	1.953680862	1.24E-15	Liver
57124	1.954538306	6.52E-26	Liver
10859	1.955020096	1.00E-17	Liver
23302	1.955518917	1.37E-12	Liver
23682	1.956594149	3.28E-13	Liver
6423	1.956744832	1.83E-06	Liver
100130958	1.95714515	6.08E-14	Liver
11149	1.957707627	1.19E-16	Liver
84264	1.95777063	1.40E-10	Liver
222663	1.958338299	2.74E-16	Liver
55225	1.958725956	6.87E-13	Liver
644353	1.958865521	1.27E-22	Liver
2359	1.959531421	1.67E-19	Liver
51101	1.960577531	5.99E-14	Liver
3111	1.961387836	1.50E-16	Liver
4045	1.961587574	1.37E-09	Liver
65989	1.961592397	2.45E-07	Liver
7070	1.962576404	1.45E-25	Liver
11279	1.963039999	2.50E-17	Liver
26167	1.963819941	3.34E-08	Liver
7805	1.96402835	6.59E-26	Liver
374403	1.965107078	3.04E-13	Liver
770	1.965341443	8.54E-14	Liver
56134	1.966054396	4.23E-07	Liver
5064	1.966465893	4.54E-20	Liver
6280	1.966765798	3.59E-09	Liver
126410	1.968050985	0.000462092	Liver
53827	1.970172375	3.45E-24	Liver
23414	1.970255468	1.17E-16	Liver
115207	1.970453358	5.06E-25	Liver
2047	1.971091692	4.79E-16	Liver
259217	1.971365349	2.97E-16	Liver
80128	1.972661209	2.59E-16	Liver
22996	1.972762735	7.43E-08	Liver
9181	1.973517354	1.89E-31	Liver
644538	1.973592203	8.84E-20	Liver
26034	1.974114482	4.44E-20	Liver
57415	1.97469566	9.26E-07	Liver
5125	1.974915042	4.60E-12	Liver
23017	1.975269654	1.57E-14	Liver
10516	1.976882763	2.28E-11	Liver
2123	1.977213489	1.03E-20	Liver
114879	1.977464139	5.59E-22	Liver
2662	1.977916467	1.26E-08	Liver
2744	1.977950807	3.30E-18	Liver
26301	1.978402486	7.22E-26	Liver
23708	1.97844426	5.55E-07	Liver
11074	1.979013142	1.64E-05	Liver
120376	1.979292242	1.03E-11	Liver
165530	1.979365528	4.14E-13	Liver
11254	1.979679418	3.71E-07	Liver
7561	1.98070213	1.41E-15	Liver
84868	1.980712845	1.76E-21	Liver

6775	1.981057275	2.28E-12	Liver
57835	1.981963855	1.51E-24	Liver
65055	1.98267617	6.70E-09	Liver
85360	1.982845575	2.00E-27	Liver
128611	1.983045407	5.54E-12	Liver
10538	1.983275857	1.50E-10	Liver
4681	1.984061474	1.42E-15	Liver
51566	1.98501027	1.53E-11	Liver
497189	1.985413112	2.96E-14	Liver
27295	1.985414233	1.53E-14	Liver
3738	1.985551027	3.57E-12	Liver
29774	1.986399116	5.13E-17	Liver
147138	1.987507826	1.20E-20	Liver
3903	1.987900769	2.67E-19	Liver
84957	1.988497188	1.72E-29	Liver
144406	1.988540595	2.71E-15	Liver
59345	1.990362128	1.12E-30	Liver
338442	1.990485484	7.96E-11	Liver
60489	1.99166834	1.60E-19	Liver
60598	1.992065504	3.46E-09	Liver
7694	1.992279714	3.89E-11	Liver
223117	1.992451621	3.78E-10	Liver
219539	1.993118872	6.50E-27	Liver
23413	1.994088629	4.59E-13	Liver
3750	1.994702693	1.59E-23	Liver
56271	1.995342458	9.87E-21	Liver
83706	1.995555522	1.45E-27	Liver
56667	1.995763674	0.000836979	Liver
253152	1.996111705	7.72E-15	Liver
2182	1.997026997	3.65E-07	Liver
1028	1.997923994	1.34E-12	Liver
57232	1.99847128	1.42E-13	Liver
1290	1.99852586	3.70E-20	Liver
695	1.999068238	1.33E-21	Liver
11167	2.000195669	5.32E-21	Liver
57452	2.000565305	4.39E-12	Liver
158228	2.000662415	7.26E-08	Liver
200634	2.0031636	0.000116277	Liver
4776	2.00464749	7.57E-19	Liver
85477	2.005072832	4.87E-10	Liver
91775	2.00525985	2.30E-19	Liver
9262	2.005479771	2.96E-21	Liver
145407	2.006045426	1.42E-12	Liver
57801	2.008442581	1.13E-15	Liver
26499	2.008508598	4.23E-06	Liver
89832	2.009432268	1.63E-13	Liver
8876	2.009509136	3.32E-05	Liver
54836	2.009600092	6.88E-07	Liver
441168	2.009631357	4.16E-15	Liver
136895	2.009851817	7.79E-13	Liver
2122	2.010100771	4.69E-24	Liver
55816	2.010903659	1.92E-12	Liver
29800	2.011035578	1.33E-16	Liver
54440	2.011508778	1.97E-22	Liver
6252	2.011925367	2.88E-14	Liver
79850	2.012265241	5.02E-27	Liver
399668	2.01284744	6.93E-15	Liver
5737	2.013178734	6.37E-05	Liver

963	2.013464228	6.96E-21	Liver
55861	2.014412637	1.88E-27	Liver
162963	2.014454586	4.01E-12	Liver
91179	2.014465905	5.24E-24	Liver
140886	2.014650543	1.09E-16	Liver
347454	2.014960429	1.45E-20	Liver
3214	2.015296567	1.45E-23	Liver
26996	2.015956067	7.38E-20	Liver
57489	2.017892189	1.46E-17	Liver
7535	2.018002596	1.31E-15	Liver
5294	2.018241701	1.03E-14	Liver
1588	2.019224397	5.01E-06	Liver
8744	2.019814635	9.71E-10	Liver
153478	2.020482328	2.42E-07	Liver
3937	2.022344751	9.65E-25	Liver
2348	2.022438305	5.09E-07	Liver
81706	2.022828928	8.29E-10	Liver
8395	2.02338709	1.04E-10	Liver
56253	2.023982647	3.53E-14	Liver
147081	2.02536703	3.89E-13	Liver
51196	2.025608265	4.99E-20	Liver
91624	2.026032197	3.11E-17	Liver
4854	2.026305598	4.93E-23	Liver
401237	2.026402579	1.73E-15	Liver
187	2.026628996	3.44E-16	Liver
56126	2.027004814	5.76E-14	Liver
140883	2.027430152	3.95E-14	Liver
776	2.02825843	1.92E-09	Liver
94120	2.028583226	7.17E-16	Liver
83982	2.0287613	1.43E-15	Liver
2867	2.029092165	6.44E-12	Liver
10544	2.03158779	1.08E-22	Liver
90649	2.032686119	1.11E-11	Liver
2303	2.033162758	2.01E-20	Liver
284759	2.034371958	4.43E-15	Liver
5241	2.034377229	4.53E-10	Liver
7480	2.034483476	1.96E-14	Liver
57121	2.035076928	1.80E-19	Liver
27180	2.035305082	1.69E-19	Liver
146722	2.035462464	2.53E-22	Liver
55786	2.035791185	6.07E-13	Liver
4602	2.036328377	2.71E-13	Liver
387357	2.036693212	1.23E-13	Liver
64499	2.037718583	3.71E-06	Liver
10154	2.03810894	1.79E-07	Liver
6558	2.038332933	5.56E-16	Liver
5031	2.038384883	7.01E-14	Liver
10335	2.03842941	1.48E-21	Liver
109	2.038749505	4.75E-30	Liver
2562	2.039543717	0.000159173	Liver
4135	2.040128391	1.00E-12	Liver
80832	2.040179104	5.67E-20	Liver
1264	2.040216361	5.80E-13	Liver
93649	2.040401241	9.50E-13	Liver
3594	2.040931139	6.59E-18	Liver
4973	2.040974916	1.58E-07	Liver
4651	2.041457493	1.64E-16	Liver
864	2.043942935	4.14E-20	Liver

7732	2.044105102	9.60E-18	Liver
79935	2.044753238	3.63E-07	Liver
28954	2.045479661	8.63E-19	Liver
1291	2.047206055	3.32E-21	Liver
286333	2.047483336	3.76E-16	Liver
10398	2.047504893	3.19E-19	Liver
55711	2.047607199	2.60E-17	Liver
3965	2.047758808	5.98E-27	Liver
116442	2.04823487	1.97E-19	Liver
7786	2.048590122	8.14E-36	Liver
8778	2.049232792	3.73E-16	Liver
5570	2.049733867	1.50E-08	Liver
3779	2.049795114	2.67E-22	Liver
79953	2.053398709	4.98E-13	Liver
54329	2.054043661	9.31E-20	Liver
558	2.054189992	1.83E-25	Liver
84251	2.054347263	7.15E-11	Liver
9805	2.055210868	1.74E-14	Liver
924	2.055599601	5.13E-12	Liver
79852	2.056035077	6.98E-15	Liver
202052	2.056231441	1.47E-31	Liver
6362	2.056573313	3.00E-09	Liver
79413	2.056597553	9.90E-12	Liver
79574	2.056829298	0.000108979	Liver
169792	2.056923656	2.11E-13	Liver
126393	2.056971183	8.24E-10	Liver
50810	2.058078263	1.17E-23	Liver
92521	2.058138437	4.63E-13	Liver
10046	2.059157501	1.62E-14	Liver
894	2.059461785	2.86E-24	Liver
160364	2.061639514	5.38E-15	Liver
259307	2.061679838	1.19E-19	Liver
7421	2.062735219	5.22E-10	Liver
1536	2.064799601	9.57E-17	Liver
84838	2.065355955	6.22E-15	Liver
6535	2.066084421	5.74E-09	Liver
64855	2.06655483	6.51E-27	Liver
169834	2.067670349	6.22E-09	Liver
55655	2.067671879	2.49E-08	Liver
54704	2.068243183	1.15E-16	Liver
646851	2.068368402	3.05E-13	Liver
56099	2.068782281	4.84E-11	Liver
221914	2.069414963	8.49E-17	Liver
11226	2.069914704	2.97E-26	Liver
2696	2.071180247	2.15E-12	Liver
2150	2.071285459	1.92E-07	Liver
9021	2.072166973	5.25E-16	Liver
167838	2.072912127	3.21E-11	Liver
1286	2.073531242	4.70E-12	Liver
3488	2.073819512	3.71E-15	Liver
4908	2.074667295	5.68E-15	Liver
3898	2.07503388	1.84E-06	Liver
3117	2.076541807	1.68E-14	Liver
3820	2.07687359	6.47E-12	Liver
54847	2.076952217	3.70E-18	Liver
56675	2.077137196	7.39E-20	Liver
688	2.077341748	2.91E-09	Liver
57156	2.077805612	3.01E-10	Liver

9625	2.077824074	1.76E-17	Liver
91584	2.078206	1.30E-09	Liver
4068	2.079457412	1.73E-12	Liver
28231	2.079619159	8.41E-10	Liver
1820	2.080099329	8.04E-14	Liver
7020	2.08059548	4.40E-08	Liver
83850	2.081287584	1.67E-11	Liver
84553	2.081701926	1.17E-11	Liver
90427	2.081931467	1.63E-21	Liver
64098	2.082429666	1.61E-22	Liver
6001	2.083055169	1.30E-24	Liver
5452	2.084014279	1.87E-19	Liver
57713	2.085399144	1.50E-13	Liver
10320	2.085857764	9.67E-21	Liver
1735	2.086731685	1.61E-10	Liver
5973	2.086765863	3.17E-13	Liver
2524	2.087474192	3.65E-08	Liver
23089	2.087538169	0.002043124	Liver
8972	2.087628494	2.38E-08	Liver
283869	2.08785477	6.62E-06	Liver
4605	2.090313355	1.07E-09	Liver
6943	2.090377251	5.18E-15	Liver
1233	2.091294111	4.06E-15	Liver
84695	2.091525222	1.09E-26	Liver
221336	2.091543455	9.45E-16	Liver
55789	2.091621939	1.76E-10	Liver
25975	2.091785294	1.30E-12	Liver
645166	2.092048263	1.91E-12	Liver
283349	2.092338869	3.82E-12	Liver
3071	2.093268111	1.53E-20	Liver
6092	2.093861112	3.20E-08	Liver
23208	2.095863963	2.58E-32	Liver
7148	2.096007507	2.19E-18	Liver
54498	2.096032038	9.46E-20	Liver
784	2.096340758	2.28E-29	Liver
6693	2.096399224	4.96E-21	Liver
337	2.096570002	0.003099029	Liver
26033	2.09880668	1.51E-06	Liver
9723	2.098854368	8.36E-07	Liver
150244	2.098986607	1.60E-13	Liver
5788	2.099678121	6.18E-16	Liver
145781	2.10018318	6.97E-16	Liver
80264	2.100456297	3.61E-15	Liver
7456	2.100724754	2.25E-31	Liver
7226	2.103917326	1.47E-18	Liver
3751	2.104359694	1.15E-12	Liver
3321	2.104954539	5.92E-10	Liver
23066	2.105691653	8.73E-17	Liver
8706	2.106857706	2.12E-21	Liver
7097	2.107958567	3.15E-15	Liver
10659	2.108565957	6.56E-22	Liver
170690	2.108588931	5.56E-06	Liver
89795	2.10905463	2.59E-08	Liver
5293	2.109611274	4.75E-24	Liver
56133	2.11086937	4.00E-11	Liver
114614	2.11132624	1.14E-17	Liver
25849	2.11182167	5.74E-20	Liver
56907	2.112623357	1.17E-16	Liver

7409	2.112656496	6.72E-20	Liver
55843	2.113616685	1.30E-25	Liver
84856	2.113958944	2.76E-12	Liver
440359	2.114973674	4.07E-19	Liver
27242	2.115904765	2.97E-18	Liver
55619	2.117139283	5.74E-20	Liver
4857	2.117334886	2.43E-08	Liver
401474	2.118752252	5.95E-08	Liver
26027	2.119391821	7.95E-11	Liver
54413	2.120050528	1.69E-27	Liver
116496	2.121261962	4.46E-23	Liver
11151	2.121386814	9.93E-23	Liver
5732	2.121725202	1.71E-15	Liver
548321	2.122195092	8.44E-14	Liver
306	2.122700443	1.76E-07	Liver
1380	2.123252039	1.30E-08	Liver
5178	2.124193886	1.29E-05	Liver
3003	2.124374313	1.93E-11	Liver
219348	2.124911586	1.26E-14	Liver
10045	2.125814527	8.64E-17	Liver
1378	2.126204247	2.96E-11	Liver
129049	2.126693097	3.55E-11	Liver
285513	2.127897476	6.35E-14	Liver
5328	2.128248095	5.96E-29	Liver
64092	2.128746806	3.67E-19	Liver
814	2.12940841	7.51E-20	Liver
8906	2.130057225	5.05E-20	Liver
8531	2.130425767	3.04E-23	Liver
7503	2.131784159	0.00719669	Liver
56301	2.1320698	6.03E-07	Liver
9168	2.132285275	4.60E-23	Liver
1488	2.132491451	4.05E-26	Liver
57616	2.134294423	1.08E-25	Liver
135295	2.135289597	3.34E-13	Liver
9828	2.13531731	7.78E-33	Liver
57604	2.136983091	3.09E-08	Liver
272	2.137681274	4.33E-23	Liver
94234	2.139173277	1.30E-05	Liver
29993	2.13966853	2.33E-08	Liver
11037	2.13992011	9.32E-20	Liver
5592	2.141009338	6.60E-15	Liver
313	2.141150636	2.39E-16	Liver
27345	2.141459157	1.60E-19	Liver
9770	2.141566408	1.42E-30	Liver
10518	2.14261963	1.39E-14	Liver
4744	2.143328691	2.90E-15	Liver
8870	2.144339863	1.29E-15	Liver
201305	2.145705406	1.78E-15	Liver
79822	2.146602352	2.12E-12	Liver
10457	2.146878261	1.39E-18	Liver
2707	2.147605348	3.67E-12	Liver
84439	2.147813461	1.40E-21	Liver
387882	2.148609267	3.54E-10	Liver
51311	2.150052444	3.87E-14	Liver
5327	2.151431976	4.63E-19	Liver
441518	2.152302198	5.08E-29	Liver
478	2.152502631	1.46E-12	Liver
5019	2.152634427	5.22E-11	Liver

9966	2.152900977	3.13E-15	Liver
2171	2.153241238	3.82E-19	Liver
80329	2.154495652	2.95E-13	Liver
6262	2.154530195	6.70E-08	Liver
7412	2.15576033	4.88E-13	Liver
4919	2.155831849	3.19E-10	Liver
5724	2.157555418	2.65E-16	Liver
11031	2.157688442	3.21E-30	Liver
8115	2.158220031	3.43E-12	Liver
7171	2.161062622	1.30E-42	Liver
23779	2.162180652	4.62E-09	Liver
57535	2.162783806	2.12E-11	Liver
10446	2.163142826	3.15E-12	Liver
3198	2.163565834	9.98E-23	Liver
114548	2.163832776	6.53E-21	Liver
5880	2.164239521	1.62E-22	Liver
7220	2.164569435	2.49E-17	Liver
57664	2.165864209	3.87E-14	Liver
30817	2.167069467	2.61E-23	Liver
91523	2.167971433	9.32E-25	Liver
407977	2.168252457	9.84E-10	Liver
11185	2.169162232	4.24E-12	Liver
388341	2.16995832	9.52E-23	Liver
3702	2.170256489	1.29E-14	Liver
2487	2.170328471	2.24E-21	Liver
89857	2.170487563	8.96E-24	Liver
83394	2.170855016	3.34E-13	Liver
4982	2.1709468	2.59E-07	Liver
440823	2.172158479	1.32E-16	Liver
199964	2.174393622	1.90E-11	Liver
140564	2.174631056	8.32E-19	Liver
654817	2.174911958	4.56E-14	Liver
53346	2.175532867	2.18E-26	Liver
219527	2.175977891	6.53E-18	Liver
56978	2.176017251	6.42E-19	Liver
4692	2.176198353	1.10E-14	Liver
80714	2.176960392	6.84E-20	Liver
11006	2.176982993	1.75E-17	Liver
27132	2.177140403	1.30E-09	Liver
23555	2.179005337	2.04E-25	Liver
140947	2.179425131	9.28E-15	Liver
4118	2.179446962	3.96E-13	Liver
339488	2.179719394	5.09E-19	Liver
123920	2.180640417	3.69E-35	Liver
146850	2.18073424	1.16E-26	Liver
22797	2.182805028	7.14E-21	Liver
9180	2.183145242	2.59E-12	Liver
6403	2.183762905	9.20E-10	Liver
29909	2.185742621	3.27E-13	Liver
1490	2.18779463	2.88E-22	Liver
79827	2.187943158	4.59E-13	Liver
79370	2.188467798	4.07E-10	Liver
400713	2.188566906	1.53E-13	Liver
65266	2.191388938	1.77E-05	Liver
80231	2.191490024	2.24E-22	Liver
23406	2.191657925	6.02E-29	Liver
121601	2.192835158	6.33E-13	Liver
25884	2.192873219	3.17E-10	Liver

402665	2.192892013	2.66E-11	Liver
84940	2.193412722	6.27E-17	Liver
2894	2.193589362	9.39E-07	Liver
58504	2.194388753	4.40E-22	Liver
57484	2.194549648	1.10E-14	Liver
2014	2.195646797	7.85E-24	Liver
27254	2.195847434	2.64E-12	Liver
645784	2.196113855	1.42E-13	Liver
11035	2.196789112	9.67E-24	Liver
54491	2.196922761	3.39E-22	Liver
5866	2.197829995	2.31E-22	Liver
3569	2.198272418	3.93E-11	Liver
10663	2.198984358	4.73E-14	Liver
81539	2.199179099	3.90E-10	Liver
196051	2.199916394	2.31E-12	Liver
257101	2.199940798	2.38E-14	Liver
1960	2.200020488	2.48E-18	Liver
81606	2.200603257	1.00E-33	Liver
8832	2.201126476	3.48E-15	Liver
4049	2.202092868	2.53E-18	Liver
79977	2.202453748	3.43E-08	Liver
5413	2.202563951	4.97E-18	Liver
2533	2.202920142	1.19E-19	Liver
140711	2.202926374	4.98E-14	Liver
1829	2.20348116	5.57E-10	Liver
7043	2.203789832	5.57E-25	Liver
54510	2.20407341	3.43E-21	Liver
374	2.204314045	1.08E-09	Liver
80307	2.206272931	2.19E-09	Liver
55466	2.206508129	8.80E-19	Liver
55165	2.207981392	2.61E-16	Liver
221188	2.208060165	6.26E-16	Liver
11178	2.208146891	4.75E-31	Liver
4330	2.208665801	8.84E-16	Liver
8875	2.209391809	2.15E-08	Liver
3213	2.209543756	3.62E-26	Liver
4363	2.210161151	1.40E-18	Liver
283174	2.210554941	2.91E-20	Liver
4856	2.211077931	5.69E-21	Liver
401190	2.212016224	5.50E-11	Liver
22821	2.213651589	6.14E-24	Liver
286527	2.21448314	1.62E-19	Liver
55040	2.215168826	2.39E-11	Liver
50619	2.217084962	1.96E-27	Liver
64581	2.217300872	2.68E-19	Liver
26353	2.217952575	3.54E-08	Liver
962	2.218210219	2.62E-21	Liver
9834	2.218246435	2.15E-11	Liver
29785	2.21906684	3.73E-13	Liver
29957	2.219525929	3.52E-14	Liver
2650	2.219767006	1.57E-19	Liver
80228	2.220022451	8.44E-40	Liver
92092	2.220196034	7.59E-15	Liver
11211	2.220533806	1.55E-14	Liver
222183	2.221314681	1.70E-09	Liver
2201	2.221576877	8.85E-17	Liver
64333	2.221906911	1.25E-21	Liver
51334	2.222195714	8.66E-22	Liver

388135	2.224660631	5.15E-13	Liver
3958	2.22516736	7.18E-18	Liver
1844	2.225665653	2.00E-17	Liver
89958	2.226276191	8.51E-15	Liver
130574	2.227271228	3.33E-09	Liver
9071	2.228013884	5.98E-06	Liver
219790	2.228651952	2.44E-11	Liver
27334	2.228753822	2.56E-14	Liver
3676	2.228779039	3.03E-23	Liver
4061	2.229204568	3.16E-12	Liver
219833	2.230900057	4.29E-18	Liver
7423	2.231248191	2.63E-14	Liver
387104	2.231725424	2.77E-17	Liver
100188953	2.231734531	1.93E-18	Liver
10344	2.232049208	2.14E-14	Liver
4773	2.232781203	5.99E-10	Liver
120114	2.233161287	2.50E-16	Liver
3912	2.235802632	2.66E-30	Liver
283383	2.238511767	1.23E-06	Liver
54863	2.238729907	2.01E-29	Liver
969	2.238971267	1.84E-15	Liver
10257	2.23927699	3.95E-10	Liver
56833	2.239684871	1.67E-20	Liver
10319	2.239804264	1.68E-12	Liver
5979	2.240012291	3.22E-09	Liver
2205	2.240609845	1.27E-13	Liver
6356	2.241219333	3.20E-15	Liver
1439	2.241621726	3.59E-24	Liver
440193	2.241646501	2.55E-27	Liver
586	2.241698112	8.41E-22	Liver
4688	2.2419019	5.57E-19	Liver
389136	2.242995987	2.09E-14	Liver
10666	2.243338512	4.25E-17	Liver
2316	2.243645133	1.25E-27	Liver
91977	2.244861613	1.28E-21	Liver
55205	2.245358862	1.25E-36	Liver
64753	2.247366085	1.85E-20	Liver
2591	2.247698367	4.93E-12	Liver
55186	2.24827901	3.54E-25	Liver
7262	2.248419682	8.76E-13	Liver
9454	2.248874117	5.13E-30	Liver
4062	2.250469484	1.30E-13	Liver
5588	2.252520346	5.02E-17	Liver
90019	2.252925658	4.78E-08	Liver
84221	2.253450835	3.28E-12	Liver
5646	2.253671251	0.000250508	Liver
9103	2.25372742	1.17E-11	Liver
3691	2.254021573	2.53E-18	Liver
90594	2.254721135	1.88E-21	Liver
113802	2.254807972	2.36E-14	Liver
84206	2.254892699	9.46E-22	Liver
5026	2.256188354	1.93E-20	Liver
182	2.256376972	1.25E-27	Liver
26298	2.256645962	4.22E-08	Liver
27134	2.257643256	7.90E-08	Liver
9823	2.258910523	1.62E-25	Liver
92745	2.259841292	1.35E-12	Liver
6695	2.2604239	1.17E-07	Liver

27071	2.260571156	7.24E-21	Liver
2149	2.261411847	9.90E-30	Liver
9545	2.262315531	5.43E-18	Liver
85409	2.262755344	5.98E-09	Liver
11156	2.263449763	1.90E-21	Liver
728215	2.263604823	1.21E-19	Liver
3959	2.264174187	3.25E-14	Liver
2833	2.264242004	2.06E-17	Liver
221935	2.264948403	1.13E-07	Liver
636	2.266841422	1.30E-19	Liver
5961	2.266861288	9.33E-21	Liver
7057	2.26733211	2.03E-16	Liver
10673	2.268930585	4.28E-18	Liver
50852	2.269501996	3.59E-14	Liver
10004	2.269583876	4.45E-13	Liver
1043	2.269801314	7.64E-22	Liver
115362	2.271251102	8.66E-14	Liver
219699	2.271630414	4.42E-30	Liver
57125	2.272876186	3.28E-25	Liver
4884	2.275427623	1.34E-11	Liver
9447	2.276291037	5.15E-13	Liver
951	2.27741532	2.57E-27	Liver
8843	2.278081677	3.98E-11	Liver
85453	2.278883117	1.26E-24	Liver
23251	2.279174562	9.18E-19	Liver
10563	2.279555975	1.40E-05	Liver
9603	2.280634129	8.15E-24	Liver
84446	2.282950171	1.95E-21	Liver
64084	2.283029296	6.93E-12	Liver
3606	2.28389468	5.15E-20	Liver
64359	2.284483781	1.05E-14	Liver
26115	2.284875551	3.90E-19	Liver
66000	2.285364077	1.98E-17	Liver
114881	2.285492084	1.58E-26	Liver
114783	2.286596642	2.32E-08	Liver
51127	2.287759717	9.13E-12	Liver
30812	2.287934719	6.57E-20	Liver
56241	2.288439536	5.22E-13	Liver
27347	2.289103142	2.54E-14	Liver
65009	2.290274491	3.36E-16	Liver
139221	2.292110633	1.62E-07	Liver
2151	2.292139955	2.79E-11	Liver
150368	2.292544152	5.40E-22	Liver
57622	2.293445126	1.17E-20	Liver
5652	2.29434649	0.000353348	Liver
201799	2.294902328	7.46E-13	Liver
81855	2.296266311	1.13E-38	Liver
2125	2.296309282	1.20E-06	Liver
54626	2.296956941	3.70E-14	Liver
728264	2.298757494	5.61E-22	Liver
56659	2.298789115	4.07E-12	Liver
84532	2.298953248	2.75E-20	Liver
10391	2.299410153	4.59E-19	Liver
5791	2.29973926	8.15E-33	Liver
4325	2.300469937	3.06E-19	Liver
25806	2.302482715	5.20E-13	Liver
3955	2.30259325	1.39E-32	Liver
25797	2.303160258	1.61E-14	Liver

925	2.303806688	1.84E-14	Liver
4300	2.304156124	4.18E-15	Liver
401494	2.306811599	2.55E-31	Liver
100130776	2.30820167	6.52E-31	Liver
9363	2.308570394	9.05E-27	Liver
5021	2.309861531	3.14E-17	Liver
60312	2.309927584	1.08E-31	Liver
5742	2.310221674	1.03E-18	Liver
57633	2.310322641	3.07E-10	Liver
2813	2.312470186	3.64E-06	Liver
57556	2.313667875	1.09E-15	Liver
4248	2.314122421	9.17E-22	Liver
201294	2.314412689	1.72E-24	Liver
5157	2.315281243	1.02E-17	Liver
84152	2.315870041	1.21E-07	Liver
43	2.316209675	4.81E-10	Liver
377007	2.3170089	1.64E-14	Liver
126306	2.317319215	4.05E-13	Liver
79986	2.31748133	6.73E-14	Liver
3036	2.317654484	5.89E-14	Liver
5778	2.318829365	1.46E-21	Liver
645369	2.319426621	1.91E-16	Liver
91409	2.322217899	1.80E-20	Liver
9123	2.323198659	2.07E-20	Liver
9957	2.323944106	1.96E-26	Liver
5831	2.324082054	1.69E-07	Liver
119	2.32609275	2.24E-20	Liver
84898	2.326534226	3.39E-18	Liver
6586	2.326835598	2.94E-17	Liver
5141	2.329296686	8.75E-26	Liver
353500	2.329431616	5.46E-21	Liver
3790	2.330210414	3.19E-15	Liver
3800	2.333373823	3.30E-14	Liver
9182	2.333610123	6.15E-12	Liver
85352	2.335185499	1.00E-16	Liver
6274	2.335237256	9.58E-16	Liver
5350	2.335754202	1.71E-14	Liver
166336	2.335840339	1.83E-16	Liver
84525	2.336037919	4.00E-23	Liver
9734	2.336271873	1.57E-22	Liver
6282	2.337870375	1.63E-23	Liver
655	2.340664568	3.53E-08	Liver
5158	2.340923138	2.30E-19	Liver
4600	2.342001763	1.73E-19	Liver
10272	2.343229072	3.17E-19	Liver
2950	2.344523171	4.25E-24	Liver
926	2.345594251	3.35E-12	Liver
23138	2.348412615	1.41E-27	Liver
1015	2.348980959	2.17E-09	Liver
51673	2.349165094	4.47E-26	Liver
50804	2.349596441	1.43E-08	Liver
50853	2.35059857	1.22E-28	Liver
1470	2.351370239	6.77E-15	Liver
1236	2.351676671	2.21E-16	Liver
124599	2.35291454	3.52E-17	Liver
6444	2.35338813	1.92E-10	Liver
5521	2.353620065	8.89E-15	Liver
253982	2.354357219	2.72E-07	Liver

952	2.354756089	3.12E-12	Liver
78986	2.355557649	3.00E-16	Liver
64073	2.356425689	4.27E-08	Liver
26230	2.357379408	1.99E-16	Liver
5139	2.358556903	3.74E-13	Liver
6504	2.358943211	4.38E-18	Liver
91461	2.359023682	1.62E-22	Liver
5800	2.359649042	4.65E-20	Liver
959	2.359882206	2.11E-16	Liver
4897	2.35992203	2.59E-06	Liver
9840	2.360553499	1.65E-18	Liver
9911	2.361533881	1.88E-27	Liver
941	2.362218603	3.62E-19	Liver
81849	2.362518977	1.32E-15	Liver
5764	2.363031619	9.33E-15	Liver
1909	2.363977073	3.41E-29	Liver
84959	2.364215423	8.80E-23	Liver
9056	2.365570616	1.19E-27	Liver
273	2.366420327	4.21E-18	Liver
285016	2.366565318	7.77E-09	Liver
100233209	2.366865973	6.44E-22	Liver
84106	2.368329405	4.92E-24	Liver
440556	2.368811783	1.16E-11	Liver
1896	2.370549894	9.27E-17	Liver
80183	2.37087872	2.86E-25	Liver
3897	2.373539292	3.05E-13	Liver
81931	2.37377171	1.47E-15	Liver
3489	2.374698743	4.45E-23	Liver
401884	2.37522926	2.79E-14	Liver
283208	2.37561708	9.31E-22	Liver
114818	2.377008571	2.52E-14	Liver
57158	2.37728084	1.11E-18	Liver
57593	2.377735907	2.30E-15	Liver
54210	2.378660123	1.79E-10	Liver
1285	2.379543112	8.83E-12	Liver
10718	2.380096508	4.82E-13	Liver
85462	2.380863696	2.66E-19	Liver
4063	2.380948539	1.26E-17	Liver
85416	2.382101909	6.36E-08	Liver
117283	2.382335555	3.13E-06	Liver
5613	2.382861782	1.12E-24	Liver
55084	2.382988006	4.70E-13	Liver
30008	2.383486102	5.04E-32	Liver
51308	2.384767603	1.94E-14	Liver
4148	2.384872296	8.10E-08	Liver
57146	2.384919145	1.03E-24	Liver
55784	2.386402002	1.64E-10	Liver
64409	2.386793041	3.09E-12	Liver
25903	2.387288933	2.75E-26	Liver
30850	2.390300157	4.80E-29	Liver
54209	2.390369333	1.61E-16	Liver
165545	2.390859126	2.62E-07	Liver
6275	2.391564515	8.52E-22	Liver
1118	2.392125456	4.17E-07	Liver
10507	2.392157847	2.33E-31	Liver
6850	2.39227025	5.26E-26	Liver
115004	2.392355172	1.15E-18	Liver
7852	2.392510932	2.29E-33	Liver

80774	2.393781829	1.80E-26	Liver
2743	2.39391644	4.02E-08	Liver
284207	2.394154109	2.90E-24	Liver
10123	2.39439003	7.18E-30	Liver
145773	2.395149096	5.09E-19	Liver
10125	2.395442921	8.45E-19	Liver
5228	2.395510011	1.22E-28	Liver
92815	2.395604175	5.64E-13	Liver
930	2.39653725	2.14E-11	Liver
10736	2.396638273	1.39E-08	Liver
10797	2.396655975	3.66E-31	Liver
54	2.397285644	7.57E-20	Liver
752	2.397751946	5.59E-30	Liver
4486	2.398576839	4.01E-11	Liver
81704	2.398703952	1.31E-23	Liver
55809	2.398759086	2.04E-34	Liver
26509	2.398936739	4.81E-26	Liver
78999	2.400500943	2.43E-26	Liver
5376	2.40302049	1.37E-34	Liver
2921	2.405183977	5.08E-14	Liver
124925	2.407683563	1.07E-06	Liver
348378	2.408653756	3.47E-15	Liver
57161	2.410771267	1.91E-23	Liver
115019	2.412332929	2.42E-12	Liver
911	2.412513938	3.64E-17	Liver
27124	2.413076576	2.16E-14	Liver
27074	2.413247975	1.50E-14	Liver
2781	2.413906823	2.21E-12	Liver
5806	2.414029957	3.54E-15	Liver
1794	2.414039353	8.50E-23	Liver
1606	2.414192197	5.99E-27	Liver
148206	2.414208936	1.82E-15	Liver
81030	2.414471729	2.50E-14	Liver
9751	2.41457406	1.35E-24	Liver
7472	2.415033992	3.53E-14	Liver
128853	2.41519336	1.55E-12	Liver
9843	2.41556363	2.52E-26	Liver
253827	2.417689942	2.29E-31	Liver
782	2.417997053	5.30E-30	Liver
2530	2.419316675	3.13E-28	Liver
10256	2.420404032	3.30E-08	Liver
81615	2.420422683	3.01E-09	Liver
340075	2.421731476	4.24E-15	Liver
27240	2.423489327	4.96E-18	Liver
85449	2.425210702	2.91E-10	Liver
1880	2.425754543	1.80E-20	Liver
84958	2.425965636	1.00E-20	Liver
64780	2.429332222	5.75E-32	Liver
118932	2.429883032	1.64E-09	Liver
6387	2.430581837	5.01E-16	Liver
56925	2.430680075	2.71E-28	Liver
5209	2.431282738	2.08E-20	Liver
55384	2.432201911	6.72E-10	Liver
6604	2.432279903	3.32E-23	Liver
11184	2.43333174	2.02E-21	Liver
53347	2.433739326	5.54E-18	Liver
653361	2.434111987	7.92E-18	Liver
3932	2.43421044	3.05E-21	Liver

3316	2.434711843	2.35E-25	Liver
6347	2.434869524	6.17E-22	Liver
1117	2.434976392	1.16E-12	Liver
7004	2.435454645	4.09E-25	Liver
9914	2.437240838	2.26E-11	Liver
23187	2.437872808	2.29E-44	Liver
54674	2.441762061	3.61E-20	Liver
203522	2.44202735	7.71E-25	Liver
196403	2.442411338	2.58E-26	Liver
4057	2.443047066	9.35E-14	Liver
1107	2.4447935	8.87E-33	Liver
26191	2.446112997	7.10E-22	Liver
9469	2.448248649	2.26E-30	Liver
1159	2.449728852	5.24E-09	Liver
6489	2.449762924	2.63E-21	Liver
10085	2.450079335	2.93E-11	Liver
56938	2.450879923	2.52E-11	Liver
10225	2.455246842	2.11E-18	Liver
152007	2.457405621	1.07E-35	Liver
284348	2.45782407	5.60E-22	Liver
51809	2.459546783	5.69E-21	Liver
2859	2.460846591	1.08E-10	Liver
90853	2.46247661	4.61E-15	Liver
2357	2.462610254	2.58E-18	Liver
79864	2.46277982	2.22E-23	Liver
11240	2.462790659	2.62E-11	Liver
7482	2.464498209	1.48E-19	Liver
286	2.465093183	1.61E-18	Liver
5522	2.465567557	2.57E-06	Liver
9058	2.468399043	9.01E-08	Liver
126374	2.468690222	6.86E-26	Liver
57549	2.468782913	1.75E-06	Liver
25999	2.470657179	5.63E-31	Liver
79170	2.47136503	3.11E-08	Liver
152485	2.47244074	1.50E-18	Liver
26960	2.473724707	1.97E-15	Liver
64170	2.473965808	2.02E-30	Liver
10231	2.475152363	2.84E-25	Liver
6622	2.47534599	1.58E-20	Liver
201191	2.476381829	2.46E-29	Liver
8440	2.476591635	2.59E-29	Liver
57596	2.478160327	5.59E-12	Liver
284749	2.478337124	6.39E-11	Liver
575	2.479264333	1.70E-15	Liver
79883	2.4803296	3.03E-21	Liver
7077	2.480602961	2.82E-28	Liver
147495	2.480715816	6.59E-09	Liver
7991	2.482926978	8.51E-11	Liver
23635	2.483559488	1.06E-23	Liver
1234	2.484434802	2.80E-22	Liver
5734	2.484503044	2.22E-27	Liver
117289	2.484744805	2.28E-22	Liver
57336	2.486996299	2.35E-23	Liver
10439	2.48866632	1.80E-14	Liver
11174	2.489494087	4.98E-20	Liver
7033	2.490005389	6.09E-12	Liver
57595	2.491148766	5.37E-20	Liver
165215	2.493109888	1.52E-26	Liver

441631	2.494038671	1.01E-17	Liver
747	2.494416428	8.65E-16	Liver
9595	2.495209921	1.83E-20	Liver
7804	2.495812147	3.07E-18	Liver
4948	2.496885065	2.26E-09	Liver
775	2.49761446	5.55E-25	Liver
3118	2.498322712	2.64E-15	Liver
3797	2.498563343	3.43E-29	Liver
525	2.500565009	4.74E-13	Liver
9052	2.505390474	3.61E-13	Liver
7040	2.507523264	1.07E-35	Liver
643	2.507573669	3.48E-16	Liver
83468	2.507721125	6.08E-24	Liver
388121	2.507754106	1.52E-15	Liver
3553	2.507994505	1.92E-21	Liver
401647	2.508027772	1.66E-08	Liver
84034	2.50808522	6.48E-31	Liver
597	2.508340414	2.68E-18	Liver
29	2.510902516	1.68E-39	Liver
493869	2.511588611	2.71E-31	Liver
83690	2.511976124	6.05E-17	Liver
3547	2.512042244	3.12E-08	Liver
146433	2.512547305	1.26E-21	Liver
916	2.512717641	1.34E-19	Liver
158326	2.513488511	8.65E-11	Liver
8321	2.513537581	1.47E-30	Liver
286676	2.514438115	2.65E-09	Liver
3680	2.515761128	4.42E-22	Liver
140606	2.518159438	7.79E-18	Liver
53831	2.520447345	9.36E-21	Liver
3614	2.522032542	1.51E-28	Liver
8425	2.523604392	2.83E-24	Liver
4139	2.523755035	5.07E-20	Liver
57699	2.523898248	2.67E-27	Liver
917	2.523980292	3.54E-18	Liver
287	2.525015656	1.40E-21	Liver
51284	2.529978493	4.66E-22	Liver
114897	2.530169035	3.93E-18	Liver
245972	2.531185412	1.20E-12	Liver
2737	2.534100104	1.57E-27	Liver
79730	2.534859453	2.27E-10	Liver
4291	2.535485707	2.67E-15	Liver
9245	2.536977526	1.58E-07	Liver
29933	2.537240872	3.07E-26	Liver
2210	2.537552773	1.47E-21	Liver
80332	2.53913019	5.63E-16	Liver
6899	2.539208208	1.32E-16	Liver
55356	2.542207425	2.80E-10	Liver
10900	2.543615371	3.08E-17	Liver
5997	2.544377586	7.58E-23	Liver
6320	2.544636385	8.31E-27	Liver
729230	2.545212016	3.59E-21	Liver
2051	2.547098846	3.69E-12	Liver
27123	2.548606447	9.94E-14	Liver
80023	2.551835101	1.31E-18	Liver
115273	2.554960303	1.19E-23	Liver
100132417	2.555520701	1.21E-20	Liver
1672	2.555732619	5.98E-06	Liver

64399	2.556299495	7.03E-12	Liver
23704	2.556318341	4.60E-22	Liver
55423	2.558228565	3.90E-16	Liver
57823	2.558853025	5.40E-19	Liver
1893	2.559445832	1.95E-30	Liver
943	2.559651886	2.89E-28	Liver
8685	2.560336219	2.14E-08	Liver
345557	2.562616239	1.95E-12	Liver
57194	2.564014862	3.50E-13	Liver
222223	2.564924107	2.04E-14	Liver
64131	2.565299425	9.58E-28	Liver
27445	2.565309482	1.53E-09	Liver
164832	2.565853332	1.16E-10	Liver
8091	2.565896139	1.50E-07	Liver
7275	2.568134886	2.23E-10	Liver
29851	2.568304266	4.84E-16	Liver
2532	2.56873324	5.95E-10	Liver
55220	2.570920308	5.50E-19	Liver
11182	2.571613021	1.23E-19	Liver
9369	2.573236585	8.53E-10	Liver
89846	2.574108758	1.29E-28	Liver
914	2.574596979	2.20E-20	Liver
9051	2.576103793	8.71E-20	Liver
9760	2.576733554	2.78E-12	Liver
22873	2.577091483	4.38E-19	Liver
25960	2.57852496	2.42E-34	Liver
4332	2.579308769	2.00E-27	Liver
84628	2.581815387	3.59E-22	Liver
333	2.582846184	9.39E-11	Liver
11322	2.583790511	1.94E-27	Liver
51477	2.586149064	1.43E-23	Liver
57537	2.586492134	1.49E-09	Liver
2294	2.586538679	8.18E-26	Liver
84662	2.587121017	5.88E-25	Liver
27350	2.588658551	1.05E-28	Liver
653145	2.589641523	6.76E-11	Liver
4319	2.590482099	1.50E-11	Liver
5332	2.59096368	1.16E-23	Liver
389432	2.591454817	2.17E-09	Liver
83481	2.592468068	1.19E-08	Liver
84824	2.593160451	3.41E-11	Liver
201633	2.595988893	3.07E-17	Liver
257144	2.596770823	4.90E-25	Liver
4810	2.597502845	7.21E-30	Liver
27087	2.599107664	3.74E-09	Liver
10650	2.600642007	6.08E-24	Liver
6615	2.601045572	2.30E-27	Liver
1438	2.601420427	3.41E-20	Liver
1515	2.601568314	1.66E-08	Liver
2982	2.602143843	4.09E-31	Liver
7291	2.602414532	7.46E-17	Liver
6510	2.603522628	1.02E-22	Liver
1441	2.604832293	6.65E-24	Liver
2200	2.604940364	4.17E-25	Liver
54549	2.605143405	2.85E-11	Liver
1949	2.606135004	2.18E-20	Liver
9486	2.607082639	3.13E-22	Liver
2118	2.608241937	1.49E-08	Liver

1809	2.60826357	1.38E-28	Liver
202309	2.609624061	1.91E-24	Liver
5214	2.609700823	2.74E-18	Liver
923	2.610988213	8.19E-21	Liver
23057	2.612041123	1.79E-17	Liver
1001	2.612883685	5.87E-17	Liver
55806	2.614180254	1.62E-14	Liver
6590	2.617174414	1.78E-05	Liver
1287	2.617992965	7.71E-08	Liver
57453	2.619574073	7.29E-15	Liver
2006	2.620035886	7.05E-11	Liver
58189	2.620564758	3.24E-18	Liver
57165	2.621000047	1.31E-26	Liver
7380	2.621021001	1.73E-06	Liver
114884	2.621730275	1.32E-28	Liver
91373	2.622771228	5.13E-22	Liver
64919	2.622880714	1.10E-22	Liver
9744	2.625538926	6.82E-19	Liver
440	2.626317841	9.69E-16	Liver
4958	2.626844031	6.05E-14	Liver
55561	2.628861464	1.26E-12	Liver
93010	2.629258595	1.38E-20	Liver
255231	2.630055403	4.11E-18	Liver
6785	2.630453542	4.67E-19	Liver
347902	2.632589262	1.29E-21	Liver
2529	2.634746456	4.67E-24	Liver
57683	2.635330417	7.34E-15	Liver
1303	2.636150476	2.01E-25	Liver
1305	2.638365704	8.56E-21	Liver
1271	2.63892388	5.76E-07	Liver
133418	2.639468066	8.92E-24	Liver
27112	2.63959398	4.34E-12	Liver
64005	2.640173772	3.65E-27	Liver
3221	2.640857491	4.04E-21	Liver
4622	2.640888818	0.000150013	Liver
124975	2.641068617	3.60E-13	Liver
5320	2.641465606	0.000429661	Liver
5023	2.641662478	5.28E-23	Liver
23108	2.645391613	1.06E-16	Liver
1295	2.645734414	6.89E-17	Liver
8840	2.645966059	2.73E-18	Liver
6541	2.646190415	2.46E-18	Liver
5396	2.646267836	2.91E-21	Liver
8618	2.647361397	7.56E-12	Liver
3777	2.649095081	1.89E-16	Liver
8786	2.649944706	3.32E-19	Liver
57221	2.65069611	6.28E-10	Liver
147650	2.650990921	2.35E-23	Liver
27063	2.651224943	4.28E-09	Liver
4675	2.652485042	1.26E-18	Liver
5315	2.654164982	1.25E-28	Liver
3231	2.65553801	1.06E-12	Liver
115123	2.657586681	1.81E-29	Liver
65997	2.657627283	6.62E-17	Liver
26025	2.657724224	3.66E-17	Liver
9047	2.658574933	2.16E-23	Liver
146754	2.660462331	2.28E-22	Liver
5008	2.661813827	2.60E-21	Liver

55024	2.662263904	4.37E-15	Liver
116372	2.662442397	1.66E-10	Liver
51473	2.663669451	2.90E-08	Liver
23043	2.665008947	5.42E-16	Liver
26470	2.665962554	6.19E-07	Liver
7042	2.667368793	9.68E-21	Liver
2906	2.668316481	1.28E-27	Liver
1040	2.669702376	1.52E-09	Liver
57562	2.671480854	6.83E-24	Liver
2625	2.673008836	3.58E-22	Liver
2526	2.67658093	8.52E-21	Liver
658	2.677430262	1.86E-18	Liver
57210	2.677565998	1.48E-25	Liver
6662	2.679405016	3.68E-12	Liver
2000	2.684586356	1.99E-39	Liver
11187	2.684890287	5.48E-10	Liver
8707	2.686423919	1.50E-16	Liver
283987	2.688887989	7.84E-26	Liver
50489	2.690323622	7.65E-18	Liver
140766	2.692454939	3.41E-24	Liver
913	2.692470373	2.84E-20	Liver
150726	2.694013262	1.82E-23	Liver
9641	2.696722191	1.40E-19	Liver
5325	2.698059106	5.20E-24	Liver
79674	2.699123329	6.46E-11	Liver
167681	2.699136198	9.04E-24	Liver
5602	2.701071315	1.66E-20	Liver
150365	2.702163902	8.83E-21	Liver
158471	2.702880626	9.08E-22	Liver
2260	2.704737073	9.82E-29	Liver
374907	2.707050323	1.33E-27	Liver
57689	2.707508029	6.73E-24	Liver
5069	2.707726138	6.10E-18	Liver
55959	2.707745704	2.12E-29	Liver
241	2.709884583	5.26E-21	Liver
939	2.711371857	2.55E-19	Liver
55506	2.711938632	4.78E-14	Liver
165679	2.71208403	1.06E-13	Liver
489	2.713211859	7.93E-35	Liver
54503	2.713292316	1.27E-25	Liver
8784	2.713592253	1.75E-19	Liver
124220	2.71445714	8.34E-13	Liver
84000	2.715796038	1.26E-16	Liver
54751	2.71585629	1.53E-33	Liver
8534	2.716303907	3.62E-28	Liver
10570	2.718690169	8.97E-16	Liver
6752	2.719129287	1.33E-14	Liver
1535	2.721279855	3.08E-26	Liver
915	2.722146669	5.22E-17	Liver
8506	2.726008849	7.10E-28	Liver
113146	2.726775502	1.37E-12	Liver
10855	2.726858325	1.39E-24	Liver
55092	2.727483973	1.34E-36	Liver
27181	2.727553102	5.91E-20	Liver
9148	2.728107277	2.98E-15	Liver
23148	2.728590208	5.73E-27	Liver
56924	2.731557152	1.98E-16	Liver
3561	2.731696852	1.22E-24	Liver

9022	2.733305214	1.37E-14	Liver
284069	2.73500131	3.24E-21	Liver
80008	2.736196865	4.84E-14	Liver
10637	2.736346617	1.22E-14	Liver
91851	2.738094428	2.19E-12	Liver
51316	2.738877585	2.36E-13	Liver
25907	2.74091588	2.27E-21	Liver
1364	2.742558686	3.41E-07	Liver
119587	2.742910771	6.19E-20	Liver
1493	2.743536991	6.68E-17	Liver
23616	2.744492695	7.87E-32	Liver
3559	2.745144962	8.72E-21	Liver
79705	2.745706835	3.49E-35	Liver
2318	2.749143294	3.71E-09	Liver
399	2.751998529	2.89E-24	Liver
89944	2.752658939	4.41E-14	Liver
54796	2.752991898	9.25E-22	Liver
10529	2.755133096	4.70E-11	Liver
93082	2.75556853	1.64E-12	Liver
64218	2.7561552	8.53E-32	Liver
9719	2.761258926	5.92E-13	Liver
55227	2.76319118	4.24E-14	Liver
29842	2.764726963	9.19E-18	Liver
1400	2.765743551	2.64E-26	Liver
27233	2.7657913	2.86E-17	Liver
55503	2.765876523	7.16E-11	Liver
27286	2.766541248	3.27E-20	Liver
8111	2.768914425	2.85E-29	Liver
1513	2.769808971	2.31E-30	Liver
84419	2.771273033	1.27E-10	Liver
1293	2.775093638	1.22E-29	Liver
89790	2.776245033	2.95E-29	Liver
54753	2.77643297	6.28E-30	Liver
80003	2.776819465	3.40E-22	Liver
933	2.777562749	1.58E-19	Liver
23467	2.777742708	3.01E-14	Liver
921	2.778851998	5.42E-21	Liver
100270710	2.780746881	8.15E-33	Liver
2687	2.782373504	1.03E-23	Liver
121506	2.782577148	1.28E-14	Liver
6875	2.786268378	3.18E-23	Liver
3694	2.786881398	5.58E-13	Liver
5329	2.787548621	1.05E-33	Liver
1235	2.788052705	1.23E-16	Liver
1292	2.789191865	1.27E-36	Liver
57863	2.794701473	2.55E-15	Liver
130367	2.796528319	1.38E-13	Liver
3718	2.798658878	2.58E-31	Liver
146439	2.80010687	1.04E-11	Liver
54829	2.800545885	1.38E-24	Liver
1396	2.800799277	5.14E-28	Liver
9170	2.801009769	4.09E-26	Liver
3099	2.802123531	2.46E-13	Liver
6422	2.802734606	9.84E-15	Liver
160428	2.806230664	1.11E-25	Liver
4804	2.807724513	3.37E-17	Liver
25758	2.808060258	2.34E-17	Liver
57167	2.809292049	4.17E-12	Liver

6261	2.810639243	4.74E-20	Liver
84433	2.81147592	3.21E-25	Liver
11247	2.815335404	8.42E-14	Liver
115557	2.815358232	9.46E-33	Liver
5152	2.816148916	2.69E-16	Liver
51676	2.820102869	1.73E-28	Liver
1004	2.821792857	2.90E-22	Liver
57111	2.822071409	2.72E-09	Liver
1741	2.823219432	4.55E-22	Liver
200197	2.825072033	8.38E-21	Liver
83871	2.825080079	7.11E-22	Liver
283659	2.826578382	1.85E-20	Liver
4355	2.827420126	4.37E-22	Liver
8187	2.831194603	5.01E-16	Liver
1510	2.832273942	1.66E-09	Liver
84894	2.836999027	1.16E-24	Liver
80341	2.837224166	5.41E-07	Liver
6236	2.841538439	1.32E-19	Liver
23432	2.843225211	7.20E-34	Liver
83716	2.844310616	2.68E-24	Liver
404217	2.84614587	2.15E-23	Liver
619373	2.848752976	4.72E-16	Liver
23601	2.850450487	1.53E-22	Liver
114905	2.850804565	1.49E-22	Liver
340024	2.851530614	8.52E-09	Liver
79695	2.853814596	4.02E-14	Liver
64063	2.858245742	2.78E-13	Liver
8483	2.86205097	3.38E-17	Liver
576	2.865568071	1.09E-21	Liver
146336	2.866879626	3.77E-08	Liver
6277	2.867102123	2.76E-24	Liver
342035	2.867952114	4.17E-11	Liver
79887	2.868062853	6.09E-20	Liver
7754	2.86812227	7.96E-15	Liver
27319	2.868176895	9.23E-20	Liver
150696	2.868819664	1.93E-13	Liver
100128385	2.86921833	1.03E-28	Liver
81793	2.871116555	1.99E-22	Liver
8522	2.871582925	1.22E-35	Liver
5360	2.872223589	4.46E-25	Liver
9514	2.873833893	2.93E-11	Liver
56603	2.87525427	5.08E-14	Liver
9900	2.877299764	1.46E-29	Liver
56944	2.880403503	9.64E-28	Liver
4629	2.881842224	1.55E-17	Liver
2209	2.883278544	2.94E-20	Liver
1627	2.884144472	1.41E-34	Liver
23255	2.884917731	1.10E-19	Liver
6659	2.887292124	6.91E-24	Liver
3872	2.887430319	1.13E-10	Liver
1382	2.896035422	2.95E-18	Liver
5010	2.896861519	1.14E-17	Liver
860	2.898151368	3.05E-26	Liver
80326	2.899040351	3.17E-27	Liver
10371	2.900640855	1.08E-18	Liver
83416	2.900813063	1.73E-14	Liver
8510	2.901081743	3.06E-20	Liver
822	2.902292938	1.95E-26	Liver

79734	2.903474443	1.58E-20	Liver
9721	2.90364338	4.67E-14	Liver
84870	2.905888258	2.74E-14	Liver
2302	2.907509128	1.43E-10	Liver
53832	2.908328916	8.67E-12	Liver
1846	2.909745202	5.84E-26	Liver
6853	2.910297375	1.19E-22	Liver
1755	2.910337779	9.13E-10	Liver
353189	2.913122314	1.09E-09	Liver
6285	2.913620463	5.07E-24	Liver
5133	2.914452527	7.29E-19	Liver
23627	2.91482131	5.09E-19	Liver
23641	2.915198197	1.40E-24	Liver
50515	2.916016128	1.75E-28	Liver
26508	2.916658117	2.39E-33	Liver
405753	2.917393207	4.70E-09	Liver
57705	2.91871535	3.25E-28	Liver
10279	2.919981519	1.05E-11	Liver
9244	2.920222135	4.79E-18	Liver
260436	2.921997637	1.07E-09	Liver
55013	2.922121792	7.59E-27	Liver
57817	2.922646882	3.03E-07	Liver
10409	2.923073872	1.44E-24	Liver
4058	2.92418756	1.10E-19	Liver
8537	2.924643158	8.39E-11	Liver
23423	2.925253923	4.80E-20	Liver
81792	2.9317018	3.05E-19	Liver
8839	2.931754875	5.41E-14	Liver
6696	2.934690406	5.87E-07	Liver
288	2.936104488	1.99E-18	Liver
53335	2.936695338	4.81E-18	Liver
143689	2.93746542	7.00E-20	Liver
8324	2.93841809	7.21E-28	Liver
4326	2.938901135	5.28E-14	Liver
7857	2.939137031	2.97E-23	Liver
23025	2.940448893	3.12E-16	Liver
5577	2.941176265	1.31E-25	Liver
54733	2.945786586	6.18E-28	Liver
3480	2.947777696	1.75E-26	Liver
5349	2.950203103	1.21E-09	Liver
151306	2.957658628	1.52E-30	Liver
5739	2.958864461	4.09E-30	Liver
401115	2.959505641	4.38E-15	Liver
146857	2.961565248	8.39E-18	Liver
2295	2.961964044	2.85E-26	Liver
10642	2.964169384	7.19E-07	Liver
3400	2.964225192	4.12E-25	Liver
3757	2.966622264	7.84E-13	Liver
55243	2.969452857	2.27E-19	Liver
1959	2.970991712	8.17E-27	Liver
54039	2.974146301	3.32E-21	Liver
220	2.974593519	3.10E-15	Liver
2213	2.974711782	4.18E-14	Liver
5569	2.977846231	1.20E-21	Liver
2307	2.979730904	3.20E-29	Liver
114898	2.980581281	7.72E-31	Liver
79616	2.982150214	1.67E-23	Liver
63976	2.985316101	1.53E-15	Liver

5743	2.985383157	2.86E-24	Liver
780	2.988122327	9.80E-20	Liver
6367	2.988672407	5.58E-26	Liver
4053	2.991587173	4.41E-32	Liver
3371	2.992957844	6.92E-21	Liver
3773	2.993300635	2.77E-11	Liver
10278	3.001223668	7.92E-26	Liver
146206	3.001332136	7.05E-23	Liver
2550	3.002533294	2.97E-28	Liver
3675	3.003655379	9.65E-25	Liver
8788	3.007648764	8.87E-06	Liver
64094	3.008700074	1.87E-23	Liver
5366	3.010270762	5.59E-26	Liver
54972	3.011353773	3.02E-29	Liver
6819	3.013667562	2.37E-09	Liver
84627	3.01405201	2.46E-33	Liver
1674	3.014205361	9.33E-15	Liver
346389	3.015154568	1.04E-15	Liver
131578	3.01720301	3.03E-15	Liver
256236	3.021050493	2.49E-24	Liver
79152	3.024252816	5.66E-14	Liver
79884	3.024742293	7.21E-22	Liver
55323	3.026056502	2.26E-17	Liver
2326	3.026176265	7.50E-11	Liver
4582	3.034300927	1.43E-13	Liver
10100	3.03458142	8.48E-30	Liver
135892	3.038331095	2.77E-08	Liver
79690	3.039563592	2.83E-34	Liver
10644	3.047369986	9.12E-12	Liver
3730	3.04784588	2.56E-28	Liver
652995	3.048534217	2.37E-12	Liver
152573	3.051557806	8.85E-15	Liver
3755	3.056865232	2.63E-19	Liver
1401	3.058899847	7.42E-06	Liver
3783	3.061868439	3.81E-24	Liver
2619	3.061927728	3.30E-23	Liver
730	3.063609126	6.63E-10	Liver
9143	3.066254772	7.84E-25	Liver
9609	3.066464096	1.56E-22	Liver
4130	3.06743588	1.81E-34	Liver
163933	3.06746868	1.92E-20	Liver
283248	3.072826718	1.04E-19	Liver
130576	3.074204359	1.94E-12	Liver
8912	3.075624103	1.26E-12	Liver
96610	3.088386564	2.32E-19	Liver
7130	3.089244086	1.28E-20	Liver
27129	3.092517102	1.88E-22	Liver
124842	3.095928176	6.12E-24	Liver
144165	3.098133326	7.10E-24	Liver
2903	3.101036661	2.52E-16	Liver
83742	3.102525095	1.73E-42	Liver
3082	3.102994511	3.99E-18	Liver
27293	3.104495212	6.23E-15	Liver
11033	3.107114111	1.27E-29	Liver
112399	3.110576561	4.27E-17	Liver
2568	3.112142173	8.13E-15	Liver
3481	3.113583906	2.67E-06	Liver
51454	3.115227392	1.19E-12	Liver

80201	3.115419635	9.39E-10	Liver
50861	3.11559163	2.15E-31	Liver
79365	3.11602365	1.76E-20	Liver
388610	3.124274093	2.31E-15	Liver
54478	3.125180717	5.95E-20	Liver
283120	3.125893705	1.49E-11	Liver
60681	3.126064816	7.13E-34	Liver
8612	3.130327423	7.09E-09	Liver
84168	3.132703164	3.18E-34	Liver
931	3.135234128	2.14E-14	Liver
22801	3.140658812	7.44E-29	Liver
56917	3.140992061	8.80E-34	Liver
147906	3.145511979	5.78E-35	Liver
10406	3.14627965	2.57E-10	Liver
85442	3.146522239	4.98E-09	Liver
4256	3.14972215	5.59E-29	Liver
51310	3.15839204	2.90E-15	Liver
2192	3.158464423	3.07E-23	Liver
54756	3.158670889	9.95E-33	Liver
340547	3.158983692	7.04E-15	Liver
57214	3.158994113	1.29E-13	Liver
5321	3.159750279	4.24E-31	Liver
148398	3.161952079	9.05E-15	Liver
26279	3.163481555	2.02E-14	Liver
6523	3.16632767	3.10E-12	Liver
50615	3.166991398	5.42E-25	Liver
827	3.167209398	2.07E-10	Liver
3575	3.174370435	6.36E-16	Liver
147798	3.177062966	8.57E-16	Liver
4070	3.180892749	6.65E-14	Liver
50649	3.181282244	5.99E-17	Liver
148252	3.181998802	8.79E-17	Liver
25827	3.183723638	7.52E-28	Liver
220963	3.185665035	2.01E-10	Liver
10551	3.185846593	1.07E-07	Liver
3576	3.186611688	7.46E-12	Liver
9508	3.188107492	7.52E-26	Liver
3662	3.188992042	1.07E-20	Liver
4050	3.19220947	2.39E-26	Liver
90161	3.193838237	2.98E-11	Liver
2199	3.194035313	4.66E-19	Liver
51339	3.19794746	8.26E-32	Liver
55283	3.19833429	3.13E-13	Liver
2152	3.200183161	2.69E-28	Liver
84707	3.205281079	9.74E-10	Liver
6690	3.205796886	8.83E-06	Liver
129642	3.20629399	7.27E-28	Liver
7477	3.20782843	2.51E-14	Liver
57402	3.210533632	2.87E-12	Liver
633	3.212755367	8.98E-35	Liver
5996	3.213042847	5.00E-22	Liver
80117	3.214771691	1.34E-14	Liver
27344	3.215216123	1.94E-11	Liver
122618	3.226155382	3.64E-27	Liver
79966	3.228724285	4.63E-26	Liver
771	3.228992366	5.94E-11	Liver
79983	3.230254444	7.50E-11	Liver
221002	3.234351208	4.19E-16	Liver

203111	3.239263749	7.37E-09	Liver
50512	3.23958544	1.21E-09	Liver
23416	3.239970862	1.10E-21	Liver
25825	3.242412077	9.28E-24	Liver
3957	3.243219998	6.63E-20	Liver
90557	3.245123754	1.98E-29	Liver
84033	3.245316005	1.68E-18	Liver
54587	3.246556851	1.92E-33	Liver
84935	3.247387925	7.79E-28	Liver
79679	3.24964479	2.56E-12	Liver
2026	3.251740759	6.56E-32	Liver
114801	3.261342412	3.66E-29	Liver
286133	3.261443034	1.30E-15	Liver
25890	3.268146251	1.61E-24	Liver
63876	3.268881377	2.55E-28	Liver
2735	3.273380744	2.23E-35	Liver
3651	3.273762212	6.47E-11	Liver
171177	3.275352388	1.80E-17	Liver
79804	3.279293613	1.37E-22	Liver
10164	3.280895358	1.61E-11	Liver
9200	3.282032646	2.18E-22	Liver
23231	3.28418255	1.51E-25	Liver
22925	3.284948075	4.29E-25	Liver
222865	3.285594295	1.25E-16	Liver
57476	3.287028909	2.14E-14	Liver
84620	3.293429391	3.56E-22	Liver
26032	3.296554613	2.14E-23	Liver
54509	3.298010172	4.06E-19	Liver
54852	3.301096728	8.92E-13	Liver
6533	3.3013049	8.09E-29	Liver
56967	3.303734136	4.99E-20	Liver
387914	3.309440804	4.88E-28	Liver
123036	3.310343041	2.92E-19	Liver
56937	3.315413138	4.38E-30	Liver
321	3.320647467	3.48E-24	Liver
5266	3.323143898	1.48E-11	Liver
54997	3.323145047	1.09E-17	Liver
56704	3.332254817	7.47E-20	Liver
26011	3.332774213	9.20E-25	Liver
3120	3.333529225	4.08E-26	Liver
199221	3.336741022	2.03E-35	Liver
4321	3.338254403	7.56E-13	Liver
79148	3.339973496	2.15E-27	Liver
26585	3.351312689	8.58E-14	Liver
10715	3.35416054	1.20E-18	Liver
8406	3.356030958	4.86E-22	Liver
25878	3.361768629	4.41E-26	Liver
6297	3.362924468	2.80E-20	Liver
6442	3.364101051	6.35E-25	Liver
400043	3.36854852	2.37E-28	Liver
111	3.370335001	1.70E-24	Liver
3037	3.376959131	5.51E-27	Liver
7076	3.383103358	3.12E-28	Liver
7552	3.383403661	1.67E-21	Liver
84171	3.38706179	5.89E-13	Liver
3855	3.390339601	1.62E-08	Liver
59341	3.395993782	1.56E-18	Liver
55083	3.39785867	2.00E-20	Liver

79098	3.399021221	4.81E-16	Liver
54361	3.403601474	2.21E-12	Liver
64866	3.409962727	4.62E-24	Liver
9201	3.415217272	2.54E-36	Liver
6286	3.417769071	3.54E-08	Liver
2535	3.424619223	1.35E-37	Liver
5999	3.431974632	1.87E-24	Liver
145864	3.433579305	8.88E-36	Liver
5768	3.438822105	1.07E-30	Liver
3754	3.439129878	8.80E-20	Liver
5136	3.439718446	4.89E-29	Liver
25894	3.445187477	1.27E-22	Liver
9242	3.44660766	2.86E-18	Liver
7039	3.450236162	2.45E-26	Liver
8572	3.4555126	9.32E-26	Liver
6616	3.456084459	1.32E-14	Liver
64699	3.461411775	3.28E-12	Liver
11117	3.464042197	2.50E-36	Liver
5365	3.465811733	6.28E-19	Liver
2669	3.480238915	1.01E-33	Liver
6512	3.480611732	6.44E-14	Liver
4222	3.484238189	7.96E-30	Liver
7162	3.491567114	1.08E-24	Liver
312	3.497445504	1.80E-11	Liver
8082	3.498074636	1.17E-28	Liver
9509	3.502698142	7.53E-24	Liver
387758	3.513745564	6.30E-22	Liver
5783	3.513943762	3.88E-26	Liver
2042	3.521761817	1.51E-24	Liver
22943	3.522643826	9.86E-10	Liver
6424	3.527885821	4.74E-21	Liver
83879	3.53311842	3.81E-18	Liver
5754	3.537334454	3.56E-29	Liver
647024	3.539358568	2.20E-23	Liver
240	3.541243791	1.77E-32	Liver
169044	3.54146163	6.41E-15	Liver
6364	3.54192043	6.00E-12	Liver
1805	3.545862722	9.98E-14	Liver
54825	3.548423891	9.74E-09	Liver
55203	3.551892699	7.39E-37	Liver
83987	3.553610539	4.91E-37	Liver
6344	3.556691664	1.73E-15	Liver
4969	3.557875278	1.07E-14	Liver
130271	3.558644221	3.10E-30	Liver
79987	3.558795453	4.55E-21	Liver
8437	3.561202857	1.10E-21	Liver
10331	3.568722366	1.39E-12	Liver
9902	3.572630491	4.05E-36	Liver
6649	3.580994152	8.72E-28	Liver
2121	3.581800127	5.89E-17	Liver
192668	3.589825205	4.43E-17	Liver
9358	3.593894443	6.26E-18	Liver
10290	3.597712652	5.71E-22	Liver
5099	3.601226744	1.89E-26	Liver
10536	3.6094453	2.71E-34	Liver
55228	3.614982521	3.19E-23	Liver
4320	3.627027808	6.18E-31	Liver
57419	3.62799464	1.04E-28	Liver

5802	3.640738414	2.87E-20	Liver
9353	3.643017426	3.17E-21	Liver
132884	3.646810176	4.15E-23	Liver
7301	3.647805692	6.05E-27	Liver
23532	3.65018444	2.81E-10	Liver
55061	3.655713823	6.23E-12	Liver
23149	3.657023983	2.99E-30	Liver
284422	3.658687006	2.31E-10	Liver
2300	3.663979762	2.77E-39	Liver
4440	3.664952159	3.13E-13	Liver
5450	3.666475274	3.68E-26	Liver
1281	3.67853196	1.49E-34	Liver
4922	3.682200426	1.53E-09	Liver
1278	3.693087552	1.47E-38	Liver
174	3.694992732	2.71E-09	Liver
4313	3.697312714	8.00E-35	Liver
10687	3.700507445	1.04E-33	Liver
80114	3.702065178	8.82E-16	Liver
200058	3.707277959	3.29E-30	Liver
284217	3.710600446	1.10E-18	Liver
8038	3.712111051	7.59E-27	Liver
148113	3.712983284	3.82E-29	Liver
3918	3.718454964	7.05E-17	Liver
284297	3.724998562	9.89E-36	Liver
220164	3.730042211	7.05E-32	Liver
3908	3.739820637	1.95E-21	Liver
10653	3.763388262	1.68E-24	Liver
7373	3.765192461	8.30E-26	Liver
1009	3.765617173	3.81E-32	Liver
6372	3.769214394	1.59E-12	Liver
4318	3.781672773	8.68E-22	Liver
5549	3.782866962	1.13E-24	Liver
30811	3.784130079	3.96E-20	Liver
6689	3.787725247	1.78E-23	Liver
727897	3.788073146	1.35E-07	Liver
150	3.788908875	2.11E-26	Liver
2919	3.796926719	3.26E-14	Liver
2736	3.804138591	2.28E-30	Liver
9536	3.806478352	2.33E-16	Liver
83959	3.811693884	1.04E-25	Liver
3976	3.8127515	1.67E-23	Liver
165	3.816995437	1.61E-30	Liver
144501	3.825431576	1.14E-18	Liver
176	3.832707115	1.69E-24	Liver
4241	3.83559306	4.12E-22	Liver
64220	3.835993008	1.75E-24	Liver
51148	3.836272133	3.92E-42	Liver
23024	3.841167019	3.44E-27	Liver
4885	3.841657036	5.48E-13	Liver
389332	3.842204671	2.14E-13	Liver
79669	3.84831305	2.79E-25	Liver
5307	3.859327877	2.67E-14	Liver
27299	3.87547794	2.12E-24	Liver
1902	3.876977981	1.75E-29	Liver
54869	3.877078228	3.80E-25	Liver
5270	3.883805623	2.50E-27	Liver
23213	3.888020334	1.73E-25	Liver
10863	3.894183297	2.45E-28	Liver

23768	3.894735565	3.36E-29	Liver
5744	3.896296774	1.84E-25	Liver
4920	3.898481048	1.53E-30	Liver
50863	3.900862583	2.16E-20	Liver
10418	3.903332836	1.40E-24	Liver
80760	3.91489473	4.58E-21	Liver
1289	3.924144192	1.48E-37	Liver
54102	3.925063234	2.76E-20	Liver
80206	3.925482701	3.64E-25	Liver
57216	3.947266101	6.38E-28	Liver
4588	3.963788995	3.15E-13	Liver
1298	3.974350985	8.90E-33	Liver
973	3.97468095	6.86E-23	Liver
259232	3.985381564	5.20E-28	Liver
1080	3.992132053	1.18E-14	Liver
4915	4.004878057	1.96E-21	Liver
5603	4.011114378	3.22E-26	Liver
59084	4.014306928	4.15E-20	Liver
338440	4.015217758	5.15E-29	Liver
79993	4.016931926	1.70E-16	Liver
56265	4.032338357	4.42E-34	Liver
10630	4.048937862	1.15E-25	Liver
6508	4.053830427	4.38E-23	Liver
3512	4.056740889	1.04E-19	Liver
4188	4.078268755	2.01E-37	Liver
9464	4.082703395	1.47E-23	Liver
9547	4.10785948	7.75E-19	Liver
338707	4.135897546	2.86E-17	Liver
8877	4.139110128	3.31E-27	Liver
57348	4.148694832	1.76E-21	Liver
90993	4.150907747	1.46E-32	Liver
7345	4.152956838	1.40E-17	Liver
1301	4.16559964	6.07E-21	Liver
768	4.170216865	1.51E-13	Liver
2239	4.173186125	5.86E-29	Liver
1634	4.180360036	1.86E-21	Liver
6374	4.184234554	2.42E-15	Liver
3710	4.202018616	1.63E-34	Liver
2331	4.212337415	2.53E-36	Liver
50506	4.215368228	1.65E-14	Liver
4016	4.236197269	7.56E-36	Liver
1277	4.239404186	3.63E-36	Liver
89932	4.244182098	6.80E-31	Liver
57586	4.244995969	4.08E-17	Liver
1946	4.253912274	1.05E-22	Liver
25817	4.259987025	1.92E-19	Liver
55765	4.267959555	1.13E-16	Liver
2049	4.27336045	2.46E-36	Liver
25984	4.288566998	1.36E-10	Liver
338773	4.290563801	4.29E-37	Liver
728239	4.295485465	9.85E-21	Liver
57722	4.303318188	2.41E-35	Liver
3696	4.313092241	5.84E-23	Liver
127435	4.31629254	1.19E-29	Liver
151887	4.323664115	4.40E-34	Liver
1800	4.351543451	2.65E-26	Liver
100133941	4.35654294	2.99E-16	Liver
1296	4.369281711	1.87E-43	Liver

24141	4.371083307	3.15E-26	Liver
7058	4.377282332	3.03E-28	Liver
2263	4.387066428	2.18E-13	Liver
115908	4.388216225	7.06E-34	Liver
2191	4.391355149	2.72E-34	Liver
1311	4.394715654	1.69E-21	Liver
51237	4.445255099	4.37E-23	Liver
94031	4.451120542	7.93E-41	Liver
57482	4.478753103	6.90E-39	Liver
1300	4.481055079	1.85E-24	Liver
9915	4.486248109	7.68E-28	Liver
80144	4.488298428	3.24E-22	Liver
5740	4.491317734	1.63E-30	Liver
9945	4.496130371	4.90E-33	Liver
144347	4.502162567	4.00E-28	Liver
54845	4.505557356	3.07E-16	Liver
8755	4.519309002	9.49E-25	Liver
10568	4.549417823	2.12E-19	Liver
26002	4.577562501	2.28E-33	Liver
79838	4.598522139	2.23E-17	Liver
8842	4.653493601	1.57E-18	Liver
126353	4.660882996	1.60E-18	Liver
5156	4.6943887	2.55E-26	Liver
2202	4.787845652	1.36E-26	Liver
11075	4.811569458	3.49E-27	Liver
1462	4.82569659	2.24E-37	Liver
6425	4.82992599	2.50E-18	Liver
10512	4.841947049	9.15E-43	Liver
1307	4.909095739	3.37E-40	Liver
6366	4.920891226	2.92E-22	Liver
81557	4.931452398	3.35E-25	Liver
4239	4.933826871	1.15E-28	Liver
58473	4.946033312	8.16E-29	Liver
10631	5.053568416	2.86E-31	Liver
4316	5.105907183	1.03E-20	Liver
6363	5.119882211	1.29E-23	Liver
93099	5.125909481	1.67E-21	Liver
6692	5.127158143	5.54E-28	Liver
1501	5.128174673	1.97E-21	Liver
3671	5.197613227	8.09E-36	Liver
8532	5.267176396	2.43E-34	Liver
84624	5.343933461	4.20E-37	Liver
4060	5.434421704	1.62E-27	Liver
4237	5.510283534	8.80E-47	Liver
486	5.862507004	3.11E-18	Liver
5730	5.931285578	3.77E-27	Liver
3880	6.039614296	9.50E-27	Liver
4072	6.157684638	1.62E-22	Liver
65268	6.171744459	3.09E-31	Liver
11199	-2.246102043	0.000371702	Stomach
155465	-2.10411495	1.95E-06	Stomach
342538	-2.054610397	9.04E-08	Stomach
79820	-1.993348853	5.37E-08	Stomach
266977	-1.965481696	3.39E-05	Stomach
5284	-1.928874724	0.000285588	Stomach
838	-1.869194533	1.54E-07	Stomach
9982	-1.82825586	0.000100205	Stomach
10551	-1.717996563	6.94E-05	Stomach

57016	-1.71342298	0.002931803	Stomach
246181	-1.670809628	1.40E-08	Stomach
654346	-1.652584971	2.56E-05	Stomach
4314	-1.648504226	0.000643837	Stomach
5288	-1.641539086	0.004543316	Stomach
22977	-1.606139797	2.96E-06	Stomach
1755	-1.600850951	0.008921865	Stomach
3934	-1.560120647	0.000888823	Stomach
8581	-1.532622394	0.005707727	Stomach
2568	-1.524058512	0.007899626	Stomach
151306	1.500129715	5.18E-10	Stomach
56110	1.500607973	7.52E-09	Stomach
8123	1.502374239	8.41E-07	Stomach
158880	1.502639149	1.12E-09	Stomach
83439	1.503288421	2.09E-16	Stomach
1280	1.503601693	0.000886654	Stomach
2535	1.504352228	9.69E-16	Stomach
4045	1.50437089	1.67E-08	Stomach
22881	1.504600446	4.83E-11	Stomach
140730	1.50477237	4.60E-05	Stomach
2593	1.505813954	2.08E-08	Stomach
4684	1.505998344	5.97E-07	Stomach
284367	1.506010829	2.16E-07	Stomach
1813	1.506050152	1.39E-05	Stomach
374900	1.507316162	5.57E-11	Stomach
6324	1.50757745	1.53E-15	Stomach
80816	1.50834314	2.62E-07	Stomach
3679	1.508624059	1.83E-10	Stomach
57817	1.50871298	1.88E-06	Stomach
1903	1.509754298	1.27E-12	Stomach
26353	1.510802964	3.43E-07	Stomach
164312	1.511278476	1.67E-05	Stomach
83756	1.511320813	7.59E-07	Stomach
27123	1.511397396	8.40E-08	Stomach
1805	1.513147626	4.15E-05	Stomach
285382	1.513614737	1.52E-08	Stomach
5158	1.513985005	3.77E-09	Stomach
9902	1.514932086	1.09E-16	Stomach
343637	1.514957762	0.000491039	Stomach
5345	1.515785636	1.47E-07	Stomach
114880	1.51611251	2.01E-07	Stomach
83543	1.516125751	7.54E-08	Stomach
26232	1.516714494	4.30E-05	Stomach
9509	1.516768168	9.06E-10	Stomach
114898	1.517058856	1.75E-11	Stomach
168667	1.517733085	4.75E-06	Stomach
7991	1.521337179	8.39E-11	Stomach
6366	1.521550665	0.000207544	Stomach
56138	1.521805266	0.000126776	Stomach
10417	1.523800577	8.80E-18	Stomach
23024	1.524642472	2.96E-09	Stomach
11155	1.52507555	6.81E-05	Stomach
149837	1.52540417	1.96E-10	Stomach
9717	1.525905264	4.00E-05	Stomach
3741	1.526350861	1.13E-07	Stomach
2273	1.526451582	4.65E-07	Stomach
4613	1.5267402	9.93E-07	Stomach
7499	1.526877811	4.91E-05	Stomach

114900	1.526967376	2.29E-08	Stomach
5507	1.52702234	6.05E-07	Stomach
388407	1.527054982	3.27E-12	Stomach
56660	1.527363259	7.42E-10	Stomach
56944	1.527456081	3.07E-12	Stomach
6689	1.527787693	9.82E-05	Stomach
30832	1.528072096	4.22E-09	Stomach
51676	1.528238912	1.06E-06	Stomach
183	1.528960263	5.50E-07	Stomach
56130	1.52998801	1.04E-06	Stomach
5961	1.532387733	6.92E-12	Stomach
338761	1.533415034	1.39E-07	Stomach
56135	1.533806951	4.08E-06	Stomach
8128	1.535224948	2.11E-07	Stomach
8516	1.535246345	1.15E-05	Stomach
114794	1.536031664	0.00014845	Stomach
253868	1.536354054	6.84E-06	Stomach
84627	1.537703582	3.20E-13	Stomach
84628	1.537898638	4.43E-14	Stomach
30820	1.538205563	1.41E-06	Stomach
94031	1.539295323	4.87E-12	Stomach
389136	1.542579376	1.43E-08	Stomach
50649	1.543543135	2.32E-05	Stomach
221687	1.543585177	8.63E-05	Stomach
153478	1.546376662	6.51E-05	Stomach
3623	1.546621246	3.36E-05	Stomach
10418	1.54668517	5.95E-07	Stomach
2774	1.547143314	4.63E-10	Stomach
6752	1.547186454	1.14E-13	Stomach
342897	1.547187086	0.00029748	Stomach
29942	1.54721629	1.26E-12	Stomach
8510	1.547392611	2.42E-12	Stomach
10536	1.549385496	1.34E-13	Stomach
5947	1.549823911	9.31E-10	Stomach
389658	1.549972257	1.88E-06	Stomach
51285	1.550046021	9.02E-17	Stomach
5322	1.552110097	1.02E-06	Stomach
8825	1.552851138	5.98E-08	Stomach
340527	1.552979602	2.38E-09	Stomach
2045	1.553537494	0.00033946	Stomach
2690	1.553596543	6.95E-08	Stomach
10699	1.553726191	2.59E-09	Stomach
9671	1.553936601	0.000385115	Stomach
2245	1.554314452	1.43E-20	Stomach
3231	1.556148012	1.56E-09	Stomach
7694	1.556318487	6.90E-11	Stomach
1759	1.556339073	2.95E-14	Stomach
387914	1.556674199	3.05E-08	Stomach
54749	1.55762912	6.47E-10	Stomach
3797	1.557690009	4.87E-21	Stomach
84913	1.557966721	4.49E-10	Stomach
7276	1.558348236	0.00197278	Stomach
348	1.559205728	4.00E-09	Stomach
170690	1.559519304	3.16E-05	Stomach
80852	1.559564837	2.27E-09	Stomach
6403	1.559841998	1.21E-05	Stomach
30008	1.560219002	3.74E-16	Stomach
79608	1.560883483	6.60E-06	Stomach

340706	1.561163882	0.000419498	Stomach
27287	1.562020555	3.79E-12	Stomach
55190	1.562408908	9.93E-10	Stomach
147	1.562926837	5.42E-08	Stomach
94030	1.564144317	3.33E-15	Stomach
30818	1.564629439	1.35E-08	Stomach
10216	1.564714361	5.34E-07	Stomach
3488	1.565131945	7.57E-13	Stomach
342926	1.568179108	1.04E-10	Stomach
401237	1.568418011	1.53E-12	Stomach
57710	1.568852396	4.73E-17	Stomach
29993	1.569111749	2.95E-08	Stomach
92949	1.569293493	2.41E-11	Stomach
7291	1.569865283	3.36E-09	Stomach
5443	1.570471618	3.09E-09	Stomach
3489	1.570739208	1.11E-12	Stomach
374899	1.570830202	2.06E-09	Stomach
728819	1.570879092	7.63E-08	Stomach
23414	1.571106755	1.46E-08	Stomach
23025	1.571575367	3.75E-07	Stomach
57198	1.572096192	4.70E-16	Stomach
26025	1.57346272	2.58E-09	Stomach
5649	1.574433634	1.85E-05	Stomach
59084	1.575182891	4.74E-06	Stomach
91464	1.575673426	0.002863995	Stomach
2042	1.575928112	1.78E-09	Stomach
152404	1.576285801	2.69E-06	Stomach
4036	1.577417577	0.000150527	Stomach
23641	1.578107125	8.80E-11	Stomach
10516	1.579217848	3.15E-13	Stomach
9074	1.579427971	0.003345421	Stomach
129804	1.580827323	4.31E-15	Stomach
1287	1.583859334	2.68E-06	Stomach
339524	1.583979638	3.28E-09	Stomach
8406	1.58470962	1.03E-07	Stomach
655	1.586302828	0.000113497	Stomach
5104	1.586656222	8.94E-05	Stomach
58504	1.586746278	1.56E-18	Stomach
162515	1.589092387	4.53E-10	Stomach
4747	1.589933393	2.55E-06	Stomach
283078	1.590071442	1.68E-06	Stomach
2326	1.590498808	1.67E-05	Stomach
128414	1.59126552	2.53E-06	Stomach
2814	1.591563936	9.05E-12	Stomach
129080	1.591676704	3.21E-09	Stomach
4139	1.594380541	4.99E-08	Stomach
56100	1.596558386	3.23E-11	Stomach
6876	1.596614212	1.47E-07	Stomach
115827	1.598860138	2.98E-06	Stomach
4935	1.599566549	0.000259591	Stomach
29944	1.600722981	2.42E-05	Stomach
6939	1.600822227	9.48E-11	Stomach
22873	1.602356432	7.36E-13	Stomach
11178	1.603188183	2.10E-17	Stomach
3201	1.603344303	4.10E-11	Stomach
414332	1.603440152	1.50E-07	Stomach
610	1.605391333	7.23E-10	Stomach
84215	1.605533032	7.88E-08	Stomach

387597	1.606249912	4.57E-07	Stomach
9796	1.608045008	1.83E-09	Stomach
9723	1.608292016	0.000346566	Stomach
64641	1.608712293	5.73E-07	Stomach
53947	1.609206099	2.53E-17	Stomach
1687	1.609917536	6.50E-12	Stomach
27295	1.6101834	3.52E-08	Stomach
379	1.610739444	3.30E-09	Stomach
9229	1.611089967	1.71E-06	Stomach
10406	1.611294415	0.001611528	Stomach
1307	1.613995526	3.30E-16	Stomach
196047	1.614326079	3.24E-08	Stomach
388531	1.6149932	1.10E-09	Stomach
10814	1.61579447	0.000420454	Stomach
130497	1.616185015	2.45E-07	Stomach
9719	1.617247667	1.77E-15	Stomach
4921	1.617594823	5.60E-09	Stomach
81035	1.619969539	3.74E-07	Stomach
55203	1.620117679	2.83E-09	Stomach
5354	1.620853678	0.00023941	Stomach
100128164	1.621942672	5.34E-09	Stomach
171558	1.622515243	3.22E-13	Stomach
51252	1.622553901	3.23E-05	Stomach
554	1.6225865	2.49E-09	Stomach
81557	1.623041861	1.82E-09	Stomach
335	1.623512085	0.00250306	Stomach
8277	1.623556334	0.00078593	Stomach
124925	1.624345662	8.67E-06	Stomach
50861	1.624576247	5.71E-11	Stomach
5176	1.624871735	3.35E-12	Stomach
54039	1.625548082	1.58E-07	Stomach
5624	1.626088987	1.49E-05	Stomach
84171	1.626184163	2.02E-09	Stomach
5866	1.626618573	1.18E-18	Stomach
5157	1.629809726	8.45E-07	Stomach
128209	1.630870303	5.66E-08	Stomach
25960	1.631778896	5.49E-23	Stomach
219654	1.632169656	2.35E-13	Stomach
401190	1.632254489	4.59E-09	Stomach
9823	1.632265435	2.58E-13	Stomach
79783	1.632359145	5.83E-10	Stomach
4037	1.63365003	1.33E-15	Stomach
85004	1.633777763	9.06E-09	Stomach
10991	1.634423656	0.000105399	Stomach
2899	1.634635663	2.85E-05	Stomach
140628	1.635536244	0.002684505	Stomach
196403	1.636143661	1.32E-15	Stomach
9024	1.636937862	4.96E-06	Stomach
84662	1.640440988	7.89E-20	Stomach
158866	1.640990903	1.54E-08	Stomach
7145	1.642790269	7.78E-11	Stomach
4103	1.642993151	0.002659402	Stomach
23705	1.64434939	4.83E-11	Stomach
5350	1.644758681	1.82E-05	Stomach
145820	1.64539941	9.86E-08	Stomach
6320	1.64563211	3.76E-15	Stomach
4137	1.646737123	1.90E-07	Stomach
64067	1.646861556	6.68E-09	Stomach

171024	1.646963333	3.01E-05	Stomach
10398	1.647408741	2.34E-08	Stomach
54328	1.647937107	2.70E-12	Stomach
3229	1.649079235	0.000485889	Stomach
399959	1.650341404	1.29E-07	Stomach
3899	1.650379165	4.29E-07	Stomach
56104	1.650383708	2.07E-07	Stomach
946	1.650582941	3.97E-07	Stomach
56101	1.650844462	9.70E-09	Stomach
4130	1.651175082	8.33E-15	Stomach
1780	1.651855412	1.92E-07	Stomach
590	1.652801112	2.44E-05	Stomach
22979	1.653703287	3.52E-12	Stomach
8639	1.653995893	2.61E-09	Stomach
83690	1.655312132	2.63E-10	Stomach
4674	1.65622094	2.32E-07	Stomach
339768	1.657403956	9.59E-07	Stomach
53820	1.659752775	2.98E-05	Stomach
10077	1.659929047	2.53E-09	Stomach
2674	1.660311509	7.08E-05	Stomach
9890	1.660420497	5.34E-11	Stomach
11107	1.661365631	1.40E-07	Stomach
56137	1.66188232	1.54E-05	Stomach
23432	1.662968524	5.94E-18	Stomach
2903	1.663053054	3.07E-05	Stomach
26301	1.66361372	2.45E-17	Stomach
221935	1.663978446	1.33E-09	Stomach
23743	1.664500627	9.02E-08	Stomach
338	1.664692361	0.003672044	Stomach
51148	1.66518906	8.05E-14	Stomach
57582	1.665254363	2.69E-06	Stomach
4917	1.666155441	2.74E-10	Stomach
22844	1.667094908	1.60E-06	Stomach
25903	1.667848547	3.53E-13	Stomach
55800	1.668476779	2.68E-08	Stomach
399668	1.668485478	3.04E-08	Stomach
57419	1.668518144	1.57E-10	Stomach
6900	1.668602985	2.21E-07	Stomach
1593	1.671356227	5.10E-15	Stomach
78989	1.671980386	5.83E-10	Stomach
2532	1.673132499	3.61E-05	Stomach
9340	1.673532237	5.12E-07	Stomach
5270	1.673749518	7.53E-12	Stomach
5602	1.674102649	2.25E-08	Stomach
2615	1.675053809	1.60E-18	Stomach
136227	1.675559138	2.42E-07	Stomach
9911	1.676979376	2.98E-18	Stomach
23336	1.677552798	2.53E-05	Stomach
51440	1.678321234	1.16E-08	Stomach
5999	1.680548548	5.65E-08	Stomach
286753	1.682131809	1.19E-05	Stomach
116535	1.682458496	1.09E-08	Stomach
441204	1.683619367	4.88E-14	Stomach
7482	1.684189972	1.56E-11	Stomach
146433	1.685993369	4.94E-15	Stomach
55137	1.686166827	1.60E-08	Stomach
8912	1.687887675	1.01E-11	Stomach
79605	1.688119584	1.53E-10	Stomach

651	1.690968291	0.000200397	Stomach
11075	1.691634444	5.63E-06	Stomach
57758	1.692054882	1.34E-07	Stomach
170685	1.692895743	3.13E-08	Stomach
3776	1.694041901	1.63E-05	Stomach
633	1.69483704	5.99E-15	Stomach
22801	1.696302407	2.38E-12	Stomach
56475	1.696514198	1.35E-07	Stomach
4054	1.698476676	7.10E-23	Stomach
167681	1.6989646	1.09E-08	Stomach
221458	1.698966894	8.89E-08	Stomach
2736	1.70135232	2.41E-13	Stomach
56967	1.703213306	1.16E-10	Stomach
401720	1.703839056	4.84E-18	Stomach
7545	1.70393392	1.78E-05	Stomach
1641	1.70452779	5.32E-06	Stomach
10278	1.70511175	4.85E-14	Stomach
338328	1.707955953	2.82E-09	Stomach
10316	1.708286915	8.25E-13	Stomach
4059	1.711076503	1.71E-14	Stomach
91409	1.712586755	1.51E-12	Stomach
27198	1.71376892	5.96E-06	Stomach
6586	1.714534192	1.96E-10	Stomach
56265	1.71468896	1.13E-10	Stomach
541468	1.715028934	9.28E-14	Stomach
56977	1.715398165	2.55E-09	Stomach
9066	1.715817063	7.32E-06	Stomach
7306	1.717039299	6.33E-06	Stomach
3680	1.717426384	7.57E-11	Stomach
221336	1.718840629	3.29E-10	Stomach
10382	1.718946137	1.46E-07	Stomach
6329	1.719475679	1.29E-09	Stomach
56133	1.719596473	5.67E-10	Stomach
89932	1.719907786	1.28E-14	Stomach
79948	1.720185403	9.21E-09	Stomach
163223	1.720426581	6.00E-08	Stomach
135295	1.721324001	1.23E-12	Stomach
4744	1.722679398	2.70E-11	Stomach
152641	1.723940518	3.89E-15	Stomach
6857	1.724562211	8.45E-05	Stomach
2949	1.724862906	8.31E-08	Stomach
654790	1.728745384	6.07E-06	Stomach
79987	1.729426561	3.04E-10	Stomach
6899	1.729456395	5.80E-08	Stomach
2260	1.729817394	1.55E-15	Stomach
7066	1.730332412	1.28E-07	Stomach
26038	1.73100302	2.06E-07	Stomach
80199	1.731609836	6.47E-12	Stomach
2167	1.732311144	7.94E-05	Stomach
2687	1.733284466	1.39E-17	Stomach
6332	1.734020518	2.61E-05	Stomach
1501	1.734476059	2.33E-05	Stomach
8325	1.734651918	6.93E-20	Stomach
23302	1.734920568	2.29E-10	Stomach
9899	1.735632927	7.24E-09	Stomach
55243	1.737532724	2.47E-17	Stomach
2258	1.738918951	3.04E-09	Stomach
56134	1.742308818	1.41E-05	Stomach

5178	1.745187057	3.11E-09	Stomach
54437	1.745483463	4.69E-16	Stomach
114905	1.745707653	7.31E-08	Stomach
6543	1.746456521	1.11E-05	Stomach
149345	1.747081671	6.06E-18	Stomach
3356	1.74708671	9.26E-09	Stomach
4857	1.747255937	1.88E-06	Stomach
7512	1.74816433	0.000185684	Stomach
5837	1.748423784	4.91E-07	Stomach
285598	1.748568065	5.10E-16	Stomach
7448	1.74942614	5.52E-05	Stomach
284654	1.750096123	4.92E-08	Stomach
338707	1.750170379	0.000603316	Stomach
66004	1.750356069	7.22E-08	Stomach
1129	1.752178446	0.00017242	Stomach
80303	1.752535465	4.25E-17	Stomach
146760	1.752678122	1.24E-07	Stomach
4628	1.75277356	1.27E-14	Stomach
84446	1.753138251	4.23E-16	Stomach
84814	1.755970288	1.46E-15	Stomach
3778	1.756128501	5.14E-06	Stomach
5152	1.758223236	2.45E-08	Stomach
128344	1.759481939	4.24E-06	Stomach
79623	1.759614823	6.17E-06	Stomach
5730	1.762286352	8.06E-09	Stomach
345557	1.762530342	1.23E-05	Stomach
8927	1.762589945	6.05E-11	Stomach
9687	1.76279761	1.11E-11	Stomach
339453	1.764165075	1.09E-11	Stomach
1268	1.765474592	2.00E-05	Stomach
140710	1.765810966	1.22E-18	Stomach
114805	1.767184888	1.81E-06	Stomach
57593	1.767317708	4.50E-14	Stomach
79864	1.767580717	1.57E-12	Stomach
55816	1.768049352	2.55E-11	Stomach
81794	1.768486001	7.10E-17	Stomach
6585	1.768834303	2.94E-08	Stomach
56917	1.771265119	8.49E-18	Stomach
26086	1.771294339	5.11E-18	Stomach
494470	1.771348487	2.24E-09	Stomach
79998	1.771480601	4.25E-14	Stomach
4675	1.772135898	8.60E-11	Stomach
390205	1.772260036	2.14E-09	Stomach
8224	1.772455045	3.18E-09	Stomach
8941	1.772617248	4.94E-06	Stomach
340533	1.773707919	8.52E-07	Stomach
3237	1.774194715	4.54E-06	Stomach
4239	1.774358043	1.60E-09	Stomach
6474	1.775358308	8.53E-10	Stomach
22871	1.777412781	9.88E-07	Stomach
26002	1.777488464	9.11E-11	Stomach
146556	1.779233095	1.57E-05	Stomach
11117	1.779479433	1.23E-17	Stomach
56132	1.779625905	1.80E-12	Stomach
348093	1.782456099	1.21E-08	Stomach
57689	1.782763931	3.03E-08	Stomach
125	1.783783214	0.000553883	Stomach
53826	1.784228288	4.83E-14	Stomach

23460	1.784285838	1.18E-07	Stomach
349136	1.787715402	5.81E-14	Stomach
6616	1.789068889	1.95E-05	Stomach
56113	1.791467919	2.39E-13	Stomach
55068	1.793284863	3.27E-12	Stomach
9370	1.793328802	0.000148354	Stomach
338773	1.793927678	4.57E-14	Stomach
9945	1.794520632	8.29E-10	Stomach
5789	1.795464145	2.03E-09	Stomach
10350	1.79578616	2.15E-07	Stomach
23466	1.799254349	8.00E-16	Stomach
4135	1.800768193	6.67E-11	Stomach
145258	1.801482698	7.55E-08	Stomach
9023	1.801711448	8.74E-12	Stomach
23475	1.801971464	1.00E-07	Stomach
1820	1.805217982	5.85E-16	Stomach
11197	1.805308669	6.95E-06	Stomach
6343	1.805818925	4.06E-08	Stomach
26508	1.805889897	1.75E-19	Stomach
9542	1.806091433	7.25E-09	Stomach
4803	1.806152678	8.86E-11	Stomach
645191	1.807687178	2.53E-11	Stomach
4256	1.808186045	4.63E-09	Stomach
84063	1.811367697	1.16E-06	Stomach
50846	1.811896091	3.38E-13	Stomach
90525	1.8124315	1.89E-15	Stomach
1809	1.813461444	4.91E-12	Stomach
88	1.817230414	1.84E-05	Stomach
377007	1.81741099	5.62E-11	Stomach
26960	1.817431448	7.32E-07	Stomach
9254	1.81793773	9.31E-10	Stomach
4355	1.818650543	9.15E-12	Stomach
65997	1.818947094	7.22E-11	Stomach
4053	1.819391158	1.08E-19	Stomach
3090	1.819542104	6.15E-22	Stomach
79739	1.820138392	7.00E-07	Stomach
120114	1.820507235	3.73E-07	Stomach
2155	1.822058243	5.66E-06	Stomach
23105	1.822816977	1.03E-05	Stomach
9543	1.823896837	1.16E-06	Stomach
1038	1.826610979	1.18E-10	Stomach
85409	1.827892168	7.41E-07	Stomach
28999	1.828181941	2.71E-07	Stomach
862	1.830122938	6.30E-11	Stomach
84502	1.831521443	7.75E-15	Stomach
79603	1.833079483	5.82E-11	Stomach
23349	1.833631067	6.54E-10	Stomach
93166	1.833848317	5.58E-11	Stomach
124842	1.834867755	1.12E-11	Stomach
7483	1.835446852	2.27E-07	Stomach
185	1.837457246	4.46E-06	Stomach
79953	1.837883845	2.65E-11	Stomach
170850	1.837938053	3.05E-10	Stomach
149461	1.839643377	1.35E-08	Stomach
6660	1.840235397	6.38E-12	Stomach
100190938	1.840746968	2.18E-16	Stomach
283933	1.842316775	8.47E-18	Stomach
57453	1.842317951	1.50E-06	Stomach

7373	1.842518618	1.76E-10	Stomach
25802	1.842540835	2.87E-07	Stomach
9900	1.844856667	4.08E-15	Stomach
1747	1.845629737	3.38E-05	Stomach
1363	1.846662165	4.57E-11	Stomach
147111	1.848370148	0.001775912	Stomach
164284	1.850092121	1.14E-10	Stomach
65982	1.850352466	5.98E-14	Stomach
2134	1.851295005	2.47E-12	Stomach
151126	1.8519734	1.36E-07	Stomach
5662	1.852189024	5.36E-08	Stomach
165	1.85296032	3.36E-16	Stomach
340526	1.854940213	9.94E-15	Stomach
10391	1.856518936	9.30E-12	Stomach
220	1.857683874	1.66E-16	Stomach
26032	1.857756724	8.85E-10	Stomach
26585	1.858959414	1.01E-06	Stomach
4653	1.860830662	0.000105546	Stomach
79570	1.861735504	1.67E-09	Stomach
6330	1.864565576	1.48E-12	Stomach
341640	1.869724604	0.000232462	Stomach
119587	1.869866322	4.12E-10	Stomach
92293	1.871127612	1.08E-07	Stomach
3642	1.871620058	0.000107232	Stomach
3479	1.873118982	3.24E-08	Stomach
144165	1.875247321	6.30E-14	Stomach
29951	1.875612119	1.67E-05	Stomach
482	1.876236782	1.24E-13	Stomach
91179	1.876310545	1.46E-18	Stomach
5979	1.877067668	7.08E-07	Stomach
8839	1.878705496	4.95E-07	Stomach
7058	1.879072141	5.54E-11	Stomach
84439	1.879086557	6.89E-17	Stomach
151887	1.879134399	6.02E-09	Stomach
1380	1.879227962	0.000284275	Stomach
23416	1.879588653	1.94E-08	Stomach
5239	1.880216212	5.50E-07	Stomach
162494	1.882763987	2.63E-12	Stomach
8326	1.883200268	1.61E-09	Stomach
90113	1.885379606	2.73E-06	Stomach
3777	1.885653826	8.92E-07	Stomach
2619	1.887961117	1.27E-08	Stomach
80757	1.890093488	1.70E-18	Stomach
26220	1.891435305	3.55E-09	Stomach
126374	1.891693976	5.41E-21	Stomach
89795	1.893911612	9.71E-10	Stomach
9104	1.8941265	8.89E-07	Stomach
219348	1.89449792	4.33E-11	Stomach
64409	1.895883652	8.31E-13	Stomach
64072	1.89796267	9.14E-11	Stomach
205147	1.899455542	3.25E-07	Stomach
29106	1.899503296	1.20E-05	Stomach
57338	1.900918944	1.14E-09	Stomach
57188	1.902458665	8.90E-10	Stomach
56033	1.902929097	2.68E-08	Stomach
56111	1.903262092	6.82E-13	Stomach
5413	1.904554384	6.28E-22	Stomach
57622	1.905215082	2.88E-14	Stomach

644596	1.908860025	1.48E-14	Stomach
139728	1.910153659	7.70E-05	Stomach
2259	1.912692378	4.05E-09	Stomach
441869	1.914244095	2.73E-13	Stomach
287	1.914279337	3.93E-10	Stomach
8854	1.915302359	2.87E-06	Stomach
1583	1.916968606	1.60E-10	Stomach
85407	1.918630634	7.37E-06	Stomach
23090	1.918905329	1.06E-16	Stomach
1301	1.919793399	9.34E-05	Stomach
8722	1.920174722	4.41E-15	Stomach
4916	1.920619998	1.04E-08	Stomach
644150	1.920996951	2.77E-08	Stomach
57156	1.922425316	4.28E-08	Stomach
1740	1.922545209	1.05E-08	Stomach
64094	1.92646651	5.34E-09	Stomach
25884	1.926847142	2.86E-05	Stomach
56666	1.92793019	1.23E-08	Stomach
116931	1.931744661	3.39E-08	Stomach
30811	1.931882495	1.62E-09	Stomach
1837	1.934066311	2.10E-08	Stomach
388815	1.934681023	8.55E-09	Stomach
51339	1.936685587	8.39E-17	Stomach
4440	1.937615202	3.35E-06	Stomach
9201	1.937758404	5.13E-09	Stomach
322	1.938305763	7.51E-20	Stomach
117581	1.938517137	4.38E-13	Stomach
3625	1.939871823	9.08E-16	Stomach
9479	1.939965841	9.07E-14	Stomach
100126784	1.943243206	1.75E-11	Stomach
127435	1.943758701	3.96E-13	Stomach
388121	1.944979745	4.87E-15	Stomach
57412	1.947984883	4.28E-10	Stomach
25890	1.948271776	1.20E-08	Stomach
3908	1.949705281	5.11E-12	Stomach
56301	1.949945226	1.28E-06	Stomach
284069	1.95044984	8.01E-18	Stomach
6695	1.952549731	1.37E-08	Stomach
57216	1.953763819	3.47E-10	Stomach
63876	1.955060401	9.12E-13	Stomach
1749	1.957932375	1.46E-09	Stomach
283731	1.958396498	5.67E-14	Stomach
6663	1.959214023	2.98E-07	Stomach
26577	1.959753215	3.94E-07	Stomach
1296	1.959862077	6.28E-16	Stomach
27233	1.961055127	8.51E-15	Stomach
3757	1.96137006	3.92E-08	Stomach
57692	1.961432303	1.29E-14	Stomach
54587	1.966321292	2.01E-16	Stomach
9145	1.968679033	7.18E-12	Stomach
57526	1.97083133	7.02E-11	Stomach
6869	1.970987679	1.47E-08	Stomach
25817	1.971086069	2.71E-16	Stomach
56112	1.973647149	1.84E-13	Stomach
1141	1.974713441	7.81E-09	Stomach
255167	1.975830657	7.71E-09	Stomach
55859	1.97633433	3.68E-05	Stomach
196500	1.977290714	2.26E-13	Stomach

8532	1.978917506	8.10E-15	Stomach
54549	1.979282865	5.25E-11	Stomach
80114	1.979423384	4.67E-12	Stomach
4093	1.980797743	5.37E-11	Stomach
84258	1.980848872	1.73E-10	Stomach
1264	1.981362628	3.17E-06	Stomach
64506	1.981466882	3.75E-10	Stomach
147906	1.983085179	4.26E-13	Stomach
2850	1.983344615	1.48E-07	Stomach
729359	1.983616306	2.22E-06	Stomach
163782	1.983719003	3.19E-08	Stomach
284297	1.984524435	1.06E-11	Stomach
254050	1.985827601	1.24E-11	Stomach
126129	1.985898214	7.63E-14	Stomach
10810	1.986685178	1.75E-11	Stomach
374654	1.987571969	1.16E-20	Stomach
146	1.98894337	2.02E-09	Stomach
170692	1.990882126	5.17E-10	Stomach
54551	1.991628235	9.13E-12	Stomach
2596	1.991851942	2.36E-08	Stomach
7148	1.993961731	5.40E-11	Stomach
440073	1.997501198	5.18E-13	Stomach
2121	2.001457471	4.27E-17	Stomach
6854	2.00241413	2.13E-06	Stomach
23627	2.002458969	6.02E-10	Stomach
50651	2.002931694	3.20E-20	Stomach
5348	2.003714394	6.90E-09	Stomach
340075	2.004774063	2.12E-11	Stomach
6442	2.005335952	5.56E-07	Stomach
1735	2.005459813	3.67E-08	Stomach
115557	2.006010158	1.04E-14	Stomach
220382	2.00688444	4.91E-11	Stomach
57575	2.007790567	2.70E-06	Stomach
645369	2.012474984	1.84E-18	Stomach
85449	2.013362338	2.59E-20	Stomach
6331	2.014370755	2.64E-08	Stomach
57537	2.014431985	2.05E-12	Stomach
399474	2.015049916	1.92E-17	Stomach
64084	2.016259759	3.75E-13	Stomach
342527	2.0162813	7.32E-08	Stomach
11095	2.019048344	1.24E-07	Stomach
284612	2.019696783	2.74E-15	Stomach
576	2.019849679	2.82E-12	Stomach
579	2.019876158	5.12E-08	Stomach
7044	2.020461939	6.67E-09	Stomach
79656	2.022126421	5.06E-10	Stomach
57348	2.022289104	1.01E-07	Stomach
26108	2.023836027	9.06E-12	Stomach
4886	2.025158307	9.23E-08	Stomach
79804	2.025605559	1.77E-05	Stomach
2307	2.02644942	1.11E-20	Stomach
286133	2.026972235	8.16E-07	Stomach
152573	2.027327421	4.36E-07	Stomach
347733	2.027426402	6.29E-08	Stomach
11185	2.03081828	1.61E-13	Stomach
3768	2.030915424	2.48E-10	Stomach
7025	2.031377464	3.00E-17	Stomach
4915	2.036145563	3.85E-08	Stomach

4629	2.03877685	8.51E-06	Stomach
50804	2.039818625	7.10E-09	Stomach
51310	2.040728976	3.03E-17	Stomach
108	2.043552593	6.30E-08	Stomach
7781	2.044690257	4.15E-10	Stomach
100188953	2.045847531	3.74E-14	Stomach
23066	2.045941284	5.30E-15	Stomach
2202	2.046521105	3.57E-12	Stomach
84870	2.046530372	2.08E-08	Stomach
57030	2.046697101	2.06E-11	Stomach
24141	2.049120344	5.43E-10	Stomach
79614	2.051518935	1.26E-08	Stomach
9965	2.052768742	2.40E-06	Stomach
3036	2.054500168	2.49E-10	Stomach
575	2.055918634	1.25E-11	Stomach
1000	2.058585309	1.15E-09	Stomach
23114	2.058959759	6.20E-12	Stomach
84707	2.060148492	3.20E-06	Stomach
347252	2.060290222	1.82E-06	Stomach
340359	2.061186644	1.93E-14	Stomach
55885	2.06176799	2.33E-08	Stomach
2583	2.065830944	6.95E-15	Stomach
6327	2.067138207	1.10E-09	Stomach
166012	2.069622841	5.14E-10	Stomach
55118	2.07206054	7.56E-08	Stomach
83872	2.07236385	1.43E-12	Stomach
122618	2.072892233	5.81E-09	Stomach
256691	2.074090328	4.70E-07	Stomach
145270	2.077144592	5.01E-07	Stomach
27254	2.078142142	9.73E-12	Stomach
2556	2.078877617	3.14E-05	Stomach
10446	2.079798435	2.09E-12	Stomach
4237	2.080823691	9.38E-17	Stomach
7704	2.081638216	5.85E-08	Stomach
2894	2.081703772	7.14E-13	Stomach
56896	2.081886674	1.11E-08	Stomach
84620	2.082037884	5.72E-09	Stomach
5101	2.082794205	4.77E-08	Stomach
64344	2.08544105	5.79E-08	Stomach
7481	2.087803876	2.75E-06	Stomach
27112	2.095001311	1.89E-06	Stomach
845	2.095666436	2.49E-06	Stomach
5745	2.097753777	2.53E-12	Stomach
3798	2.099084087	4.39E-10	Stomach
389558	2.100736125	1.30E-10	Stomach
9068	2.102137856	2.40E-07	Stomach
6425	2.103622547	1.50E-06	Stomach
8153	2.106401854	1.78E-11	Stomach
5139	2.107534553	5.84E-13	Stomach
57158	2.110311896	2.13E-09	Stomach
9369	2.111193396	4.57E-09	Stomach
9468	2.11125133	5.63E-11	Stomach
222950	2.112454592	7.70E-14	Stomach
729085	2.113745979	1.67E-10	Stomach
5122	2.115362279	1.64E-07	Stomach
285313	2.116768357	1.81E-09	Stomach
460	2.117432627	2.82E-09	Stomach
3671	2.117958276	3.47E-14	Stomach

7345	2.121766564	4.75E-10	Stomach
132884	2.122918671	3.59E-17	Stomach
128854	2.123272398	2.80E-20	Stomach
3745	2.124041161	9.99E-08	Stomach
5733	2.130639036	6.07E-10	Stomach
4326	2.133482295	8.41E-16	Stomach
84553	2.133964827	8.82E-08	Stomach
23768	2.134261048	7.16E-14	Stomach
4920	2.134652302	3.49E-13	Stomach
84968	2.137345867	1.74E-08	Stomach
59353	2.139198028	2.62E-09	Stomach
3780	2.140998974	2.55E-14	Stomach
284904	2.14247055	6.26E-07	Stomach
154790	2.142741269	6.49E-09	Stomach
5063	2.143191759	3.33E-10	Stomach
26167	2.146748991	1.05E-12	Stomach
139735	2.150427094	3.46E-16	Stomach
140597	2.15323052	1.21E-06	Stomach
8745	2.15463822	2.48E-11	Stomach
9427	2.155519081	5.27E-07	Stomach
6608	2.155573725	5.96E-19	Stomach
11341	2.156107406	1.38E-05	Stomach
55323	2.157194414	5.99E-18	Stomach
375057	2.157420318	3.00E-09	Stomach
2159	2.158681705	2.35E-10	Stomach
2318	2.159106074	5.38E-08	Stomach
79776	2.161922879	5.00E-13	Stomach
4223	2.165085352	1.82E-08	Stomach
252995	2.167518102	4.64E-14	Stomach
91608	2.168266795	2.69E-12	Stomach
27022	2.168876855	5.42E-11	Stomach
392255	2.180291938	1.84E-11	Stomach
1917	2.185217757	3.28E-05	Stomach
119	2.186817061	9.80E-10	Stomach
26050	2.187826796	1.68E-09	Stomach
5731	2.188093387	2.02E-12	Stomach
2901	2.188258131	3.87E-10	Stomach
91653	2.192819896	9.33E-14	Stomach
3249	2.195631887	1.50E-05	Stomach
64399	2.195854035	2.73E-08	Stomach
64220	2.196565205	6.83E-10	Stomach
4883	2.196929832	8.86E-10	Stomach
163933	2.197416076	8.40E-15	Stomach
6545	2.197945328	1.20E-07	Stomach
50944	2.199048008	1.22E-16	Stomach
57469	2.199612704	8.91E-21	Stomach
2006	2.202432647	8.87E-15	Stomach
26011	2.204241573	2.86E-13	Stomach
222663	2.21385626	1.33E-16	Stomach
9241	2.217121956	2.17E-14	Stomach
57484	2.217973304	4.72E-11	Stomach
140862	2.218575682	4.60E-10	Stomach
126433	2.220104386	1.86E-13	Stomach
8076	2.230430723	6.80E-09	Stomach
27445	2.235826209	5.29E-06	Stomach
128853	2.238404264	1.46E-14	Stomach
51308	2.241784488	1.34E-12	Stomach
57167	2.243600221	2.12E-10	Stomach

54753	2.245095412	2.76E-22	Stomach
285498	2.245575509	4.94E-11	Stomach
3755	2.249578366	8.90E-12	Stomach
477	2.253367458	2.43E-06	Stomach
25789	2.255487346	2.57E-11	Stomach
1381	2.255495795	3.26E-07	Stomach
10900	2.257065506	7.59E-10	Stomach
1545	2.262105522	3.66E-09	Stomach
84952	2.263672584	5.55E-15	Stomach
55714	2.265153804	4.51E-09	Stomach
84417	2.270762181	7.16E-06	Stomach
23148	2.273497959	2.19E-18	Stomach
1300	2.274496692	5.06E-08	Stomach
7018	2.278440442	9.02E-07	Stomach
5596	2.278936071	1.19E-07	Stomach
6261	2.282382566	1.49E-13	Stomach
78986	2.285295089	4.60E-12	Stomach
148398	2.295305514	5.66E-16	Stomach
728464	2.296093309	1.90E-10	Stomach
192668	2.296380183	5.81E-14	Stomach
23363	2.297961075	6.92E-18	Stomach
1674	2.298205903	0.000163546	Stomach
223117	2.298874498	2.32E-10	Stomach
10351	2.301958459	1.27E-07	Stomach
1470	2.303420992	3.13E-09	Stomach
10570	2.316766343	3.18E-15	Stomach
148252	2.319395965	1.18E-11	Stomach
222223	2.320668743	3.17E-09	Stomach
727936	2.321633365	3.96E-14	Stomach
7275	2.321734416	9.95E-14	Stomach
8646	2.322088574	1.46E-19	Stomach
777	2.326129683	5.48E-09	Stomach
63923	2.327851569	1.08E-11	Stomach
6422	2.327860129	8.91E-08	Stomach
2199	2.32922507	2.29E-15	Stomach
158326	2.329286295	1.24E-08	Stomach
10715	2.331514007	3.21E-14	Stomach
115290	2.333490169	3.55E-17	Stomach
79611	2.333576591	2.15E-13	Stomach
4958	2.342993458	2.09E-09	Stomach
367	2.343622536	1.79E-13	Stomach
5563	2.346421457	1.53E-07	Stomach
222962	2.348726806	7.23E-11	Stomach
55698	2.352793964	2.70E-17	Stomach
5950	2.376564094	6.80E-08	Stomach
57633	2.377358849	1.12E-07	Stomach
4330	2.377848886	1.03E-18	Stomach
91977	2.378580879	6.12E-18	Stomach
152	2.385389231	1.83E-11	Stomach
3897	2.387537977	2.53E-08	Stomach
22943	2.387645069	4.05E-05	Stomach
55224	2.395224135	6.35E-17	Stomach
1800	2.406917669	6.37E-06	Stomach
1295	2.409115759	2.84E-15	Stomach
90485	2.417385356	3.46E-18	Stomach
2719	2.423126131	2.15E-09	Stomach
222183	2.426028981	1.34E-21	Stomach
2775	2.433922943	1.44E-12	Stomach

10736	2.445777007	1.08E-09	Stomach
126393	2.45932533	1.67E-08	Stomach
27129	2.467034971	1.22E-08	Stomach
84624	2.467665334	4.34E-11	Stomach
316	2.46797837	9.29E-10	Stomach
91584	2.475792007	7.11E-14	Stomach
56963	2.480465064	3.34E-12	Stomach
3699	2.487191753	3.66E-13	Stomach
6297	2.488607736	5.94E-18	Stomach
5502	2.49342137	3.31E-08	Stomach
2303	2.496867031	8.88E-21	Stomach
6363	2.497649001	2.80E-08	Stomach
1949	2.499465498	2.36E-20	Stomach
80332	2.505291692	7.87E-11	Stomach
57863	2.506216316	3.12E-09	Stomach
2662	2.506658991	5.69E-14	Stomach
55753	2.50744418	2.49E-07	Stomach
55228	2.507495561	6.91E-14	Stomach
56776	2.511244931	1.42E-12	Stomach
93986	2.511616761	6.70E-10	Stomach
23089	2.520267727	2.48E-08	Stomach
23532	2.523067434	1.09E-05	Stomach
2735	2.527305998	4.35E-23	Stomach
84457	2.535875739	1.51E-10	Stomach
221476	2.538433305	4.50E-08	Stomach
222865	2.538514346	7.64E-14	Stomach
3481	2.543445319	1.78E-10	Stomach
4148	2.544454476	2.12E-14	Stomach
5010	2.551768451	2.79E-15	Stomach
64388	2.556168361	9.77E-10	Stomach
2657	2.562210718	3.24E-18	Stomach
9464	2.584669258	1.98E-07	Stomach
57611	2.584826819	2.98E-21	Stomach
57595	2.594764718	7.05E-16	Stomach
388135	2.599089946	3.06E-17	Stomach
1036	2.603822407	3.26E-13	Stomach
83987	2.620461717	2.46E-20	Stomach
9244	2.624441347	2.42E-11	Stomach
5064	2.632797031	2.01E-25	Stomach
2676	2.635137356	2.66E-10	Stomach
9358	2.649667643	7.05E-13	Stomach
8483	2.665238558	4.27E-09	Stomach
23467	2.667482539	7.38E-18	Stomach
10683	2.681308428	2.59E-12	Stomach
2192	2.685005996	7.68E-17	Stomach
6508	2.689154873	3.69E-12	Stomach
10290	2.691402698	6.23E-13	Stomach
203447	2.710610393	2.55E-10	Stomach
146664	2.711620096	5.13E-13	Stomach
5549	2.715038735	7.60E-13	Stomach
1272	2.718421025	1.44E-09	Stomach
259232	2.727543024	5.07E-15	Stomach
4969	2.742543123	5.32E-09	Stomach
10642	2.746832901	1.51E-06	Stomach
111	2.749818498	4.23E-11	Stomach
4884	2.766137766	2.44E-09	Stomach
2786	2.772233106	8.11E-10	Stomach
283383	2.773120449	5.11E-17	Stomach

339983	2.793407458	1.74E-15	Stomach
1114	2.794125203	2.62E-08	Stomach
7434	2.796086851	3.45E-11	Stomach
80731	2.807522675	9.86E-14	Stomach
57452	2.819213515	3.81E-16	Stomach
8120	2.824216975	5.16E-12	Stomach
6423	2.825886165	2.49E-08	Stomach
283120	2.828028293	1.82E-13	Stomach
8643	2.875887789	1.42E-19	Stomach
128434	2.882348586	1.77E-12	Stomach
148113	2.900033843	2.09E-15	Stomach
347	2.901257836	5.56E-12	Stomach
11248	2.940160527	1.06E-16	Stomach
9353	2.981069024	3.18E-12	Stomach
27092	3.028835281	9.01E-11	Stomach
5740	3.083673604	3.41E-14	Stomach
547	3.115796567	1.20E-08	Stomach
730	3.17965264	4.76E-09	Stomach
1066	3.182894147	4.55E-13	Stomach
6424	3.193445096	9.05E-12	Stomach
9379	3.200587549	3.61E-22	Stomach
168002	3.410961059	7.73E-13	Stomach
91851	3.412231493	4.85E-12	Stomach
4804	3.421495748	2.44E-15	Stomach
27344	3.642394863	2.77E-11	Stomach
1311	3.858507362	2.04E-13	Stomach
7060	3.911091067	1.63E-12	Stomach
